# Supplementary material for: Pentacyclic Triterpenoids-Based Ionic Compounds: Synthesis, Study of Structure–Antitumor Activity Relationship, Effects on Mitochondria and Activation of Signaling Pathways of Proliferation, Genome Reparation and Early Apoptosis
Source: Cancers (Basel). 2023 Jan 26;15(3):756. doi: 10.3390/cancers15030756 (PMC9913425; doi:10.3390/cancers15030756)

Supplementary Material for

Pentacyclic Triterpenoid's Based Ionic Compounds: Synthesis, Study of Structure-Antitumor Activity Relationship, Effects on Mitochondria and Activation of Signaling Pathways of Prolife-ration, Genome Reparation and Early Apoptosis

Lilya U. Dzhemileva *, Regina A. Tuktarova, Usein M. Dzhemilev, and Vladimir A. D'yakonov *

N.D. Zelinsky Institute of Organic Chemistry, Russian Academy of Sciences, Leninsky Prospect 47, Moscow, 119991, Russia.

Table of contents

1. ^{1}H and ^{13}C NMR Spectra of the Products S3

^{1}H and ^{13}C NMR Spectra of all Products

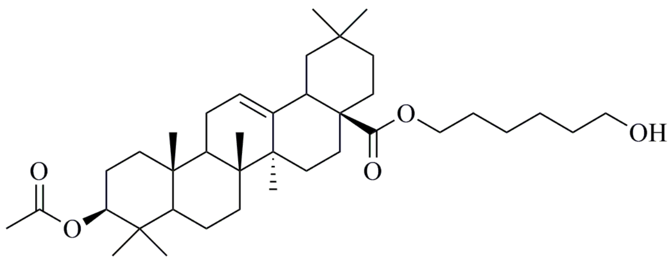

Figure S1. ^{13}C NMR Spectrum of compound **2** (125 MHz, $CDCl_3$)

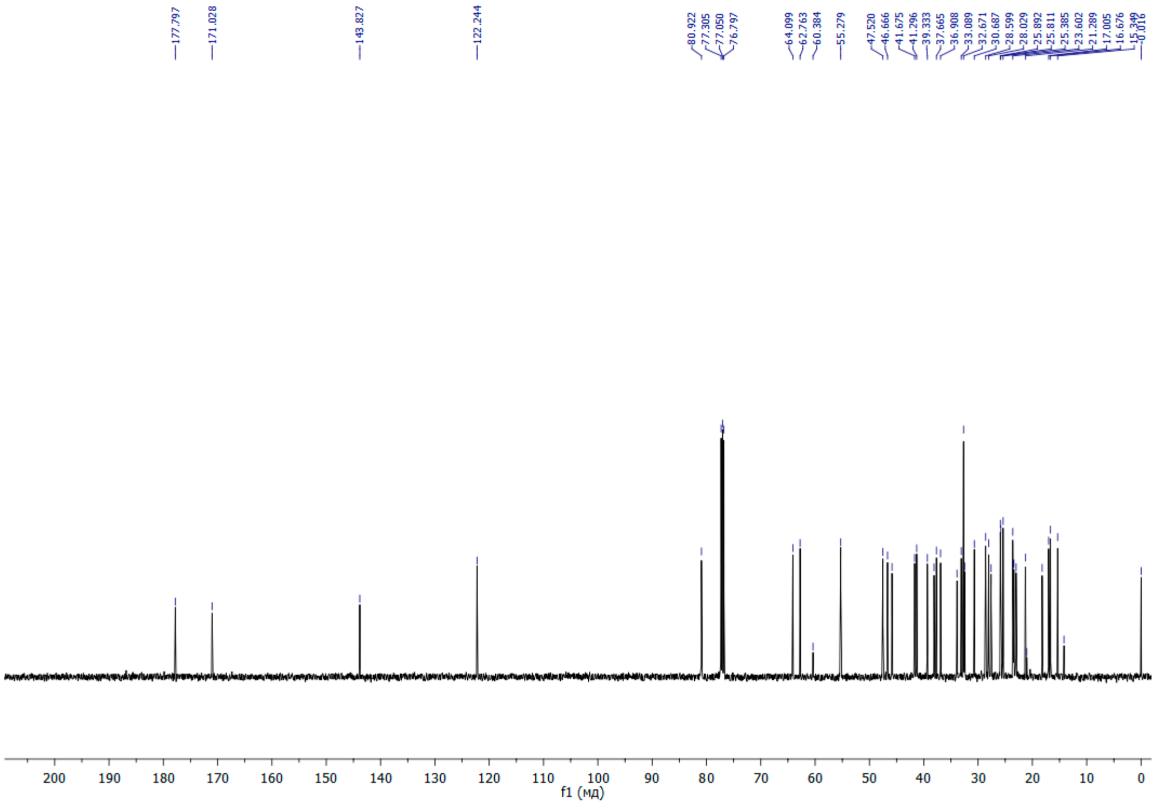

Figure S2. ^{1}H NMR Spectrum of compound **2** (500 MHz, $CDCl_3$)

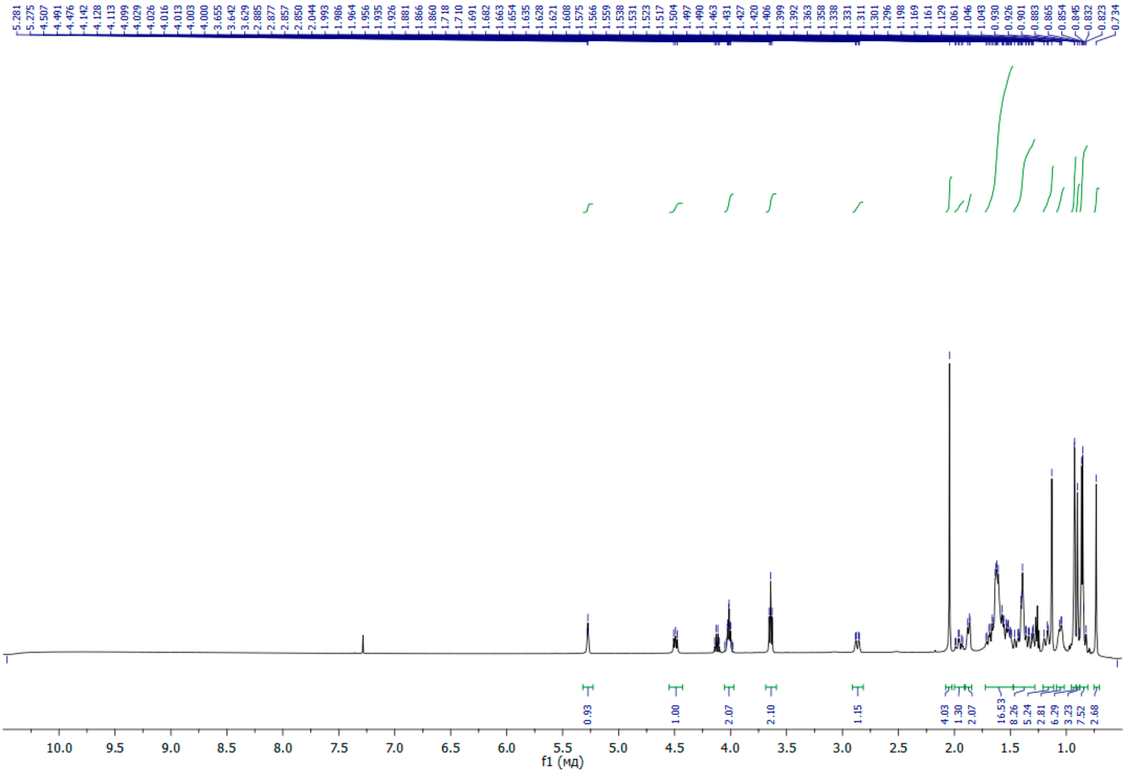

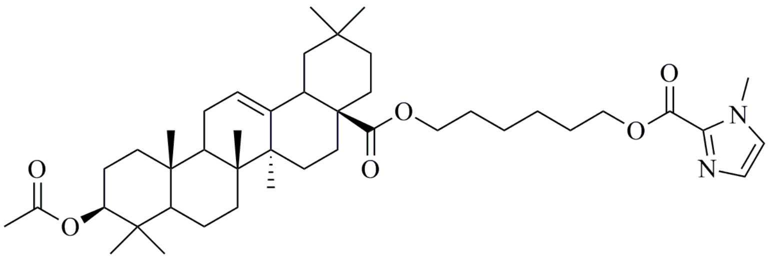

Figure S3. ^{13}C NMR Spectrum of compound **3** (100 MHz, $CDCl_3$)

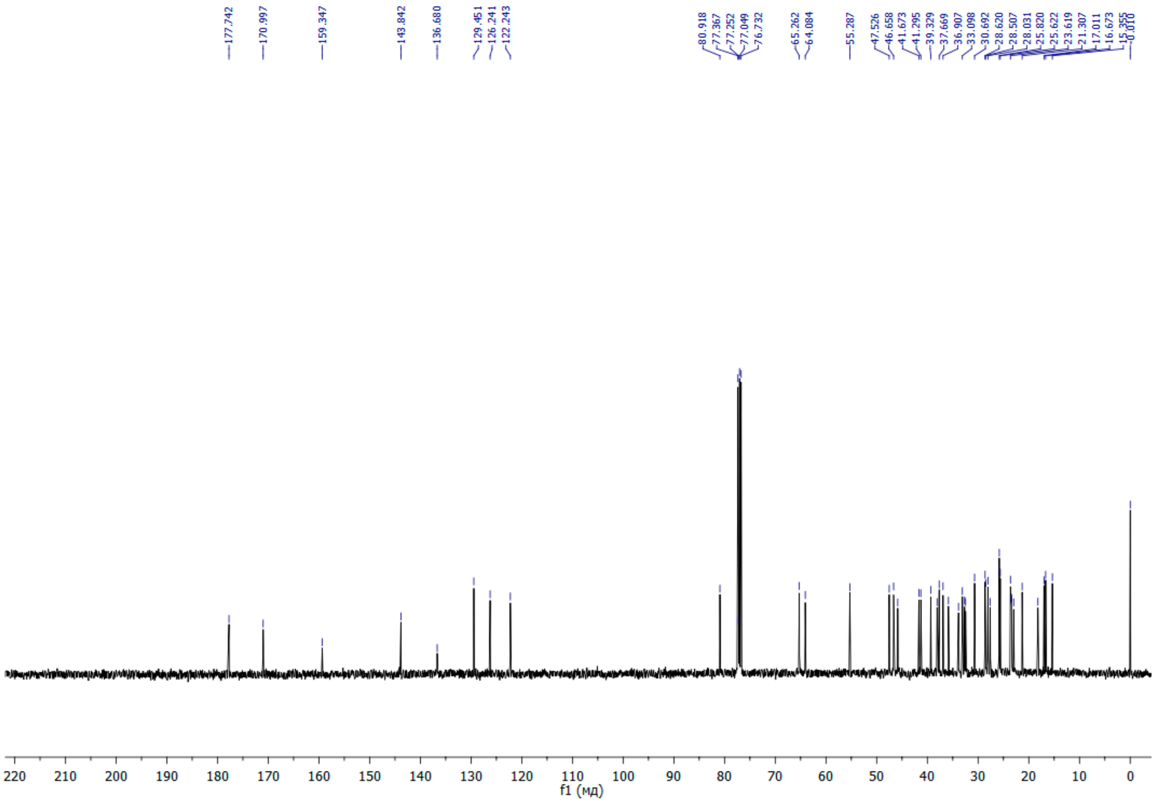

Figure S4. ^{1}H NMR Spectrum of compound **3** (400 MHz, $CDCl_3$)

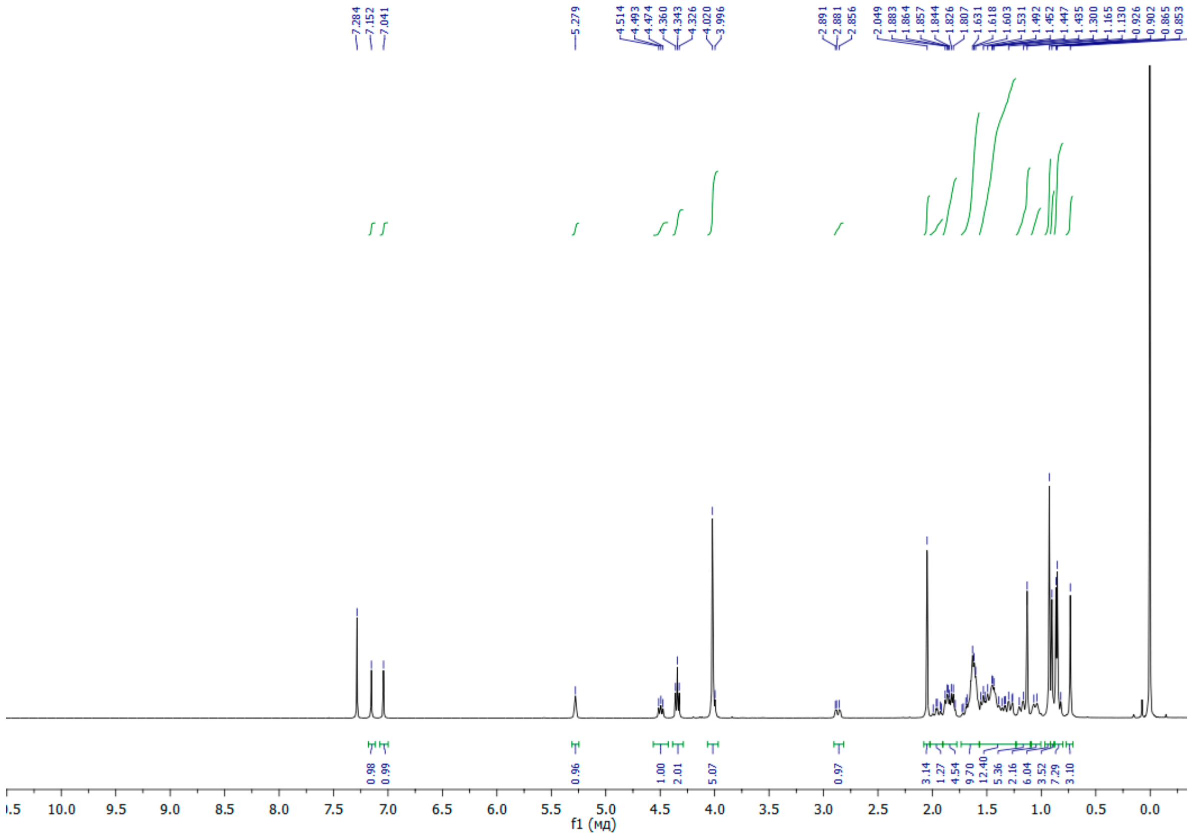

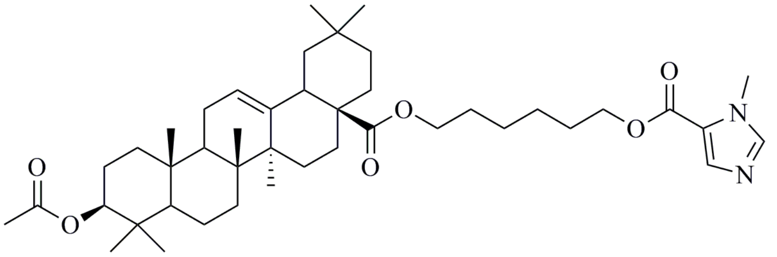

Figure S5. ^{13}C NMR Spectrum of compound **4** (125 MHz, $CDCl_3$)

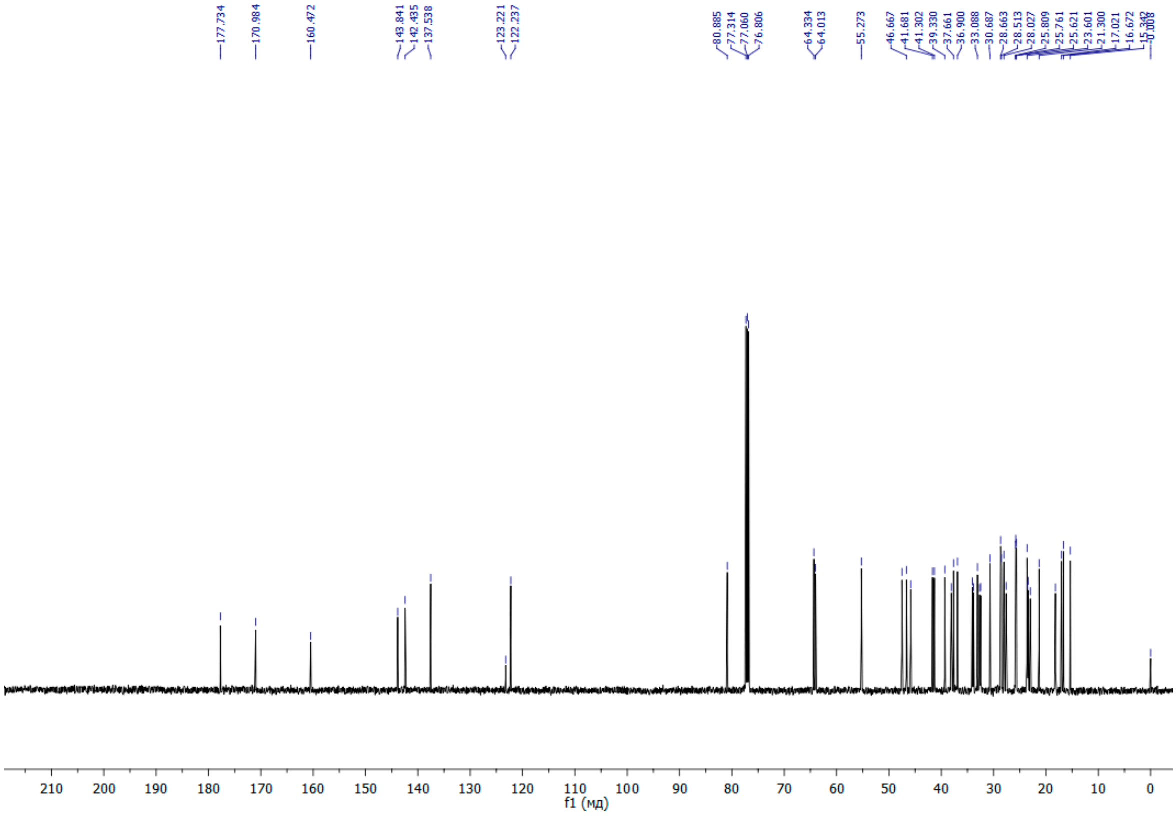

Figure S6. ^{1}H NMR Spectrum of compound **4** (500 MHz, $CDCl_3$)

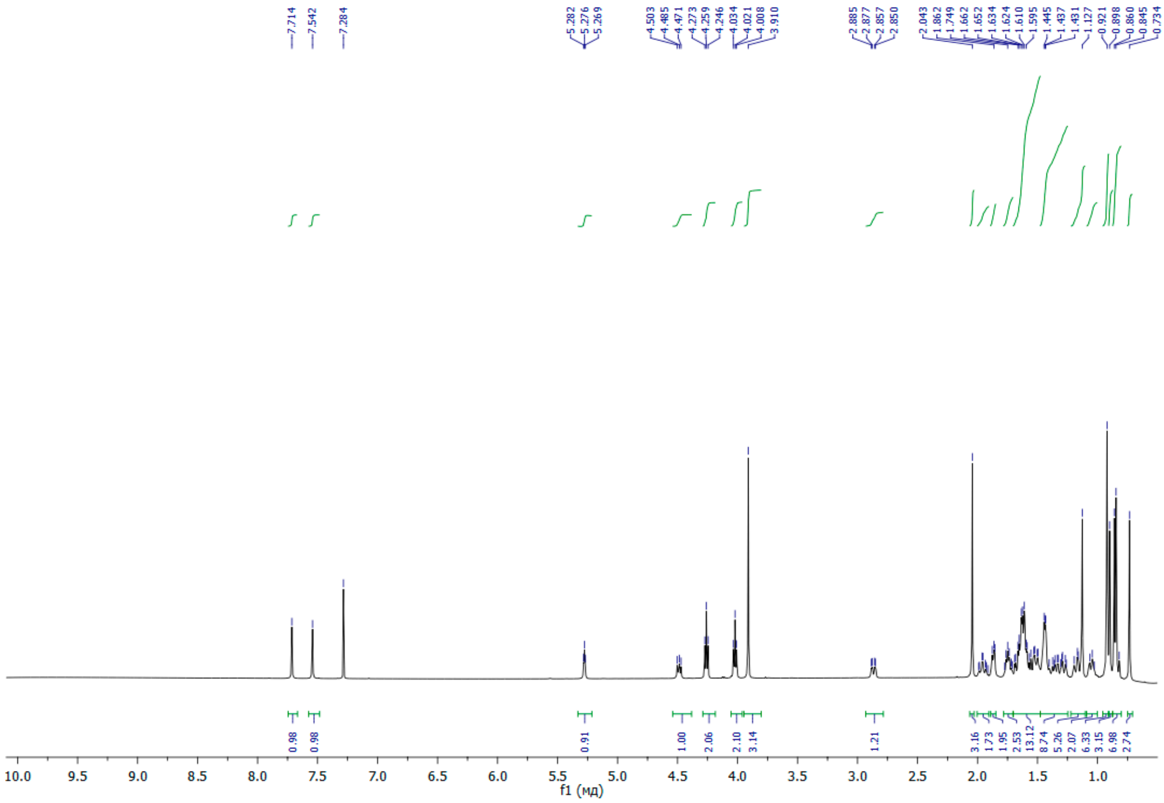

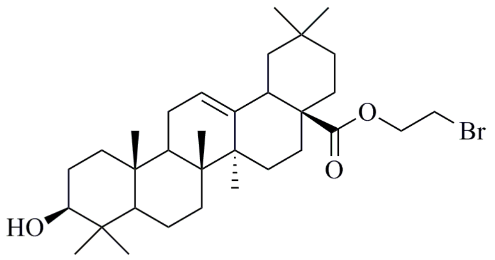

Figure S7. ^{13}C NMR Spectrum of compound **6a** (100 MHz, $CDCl_3$)

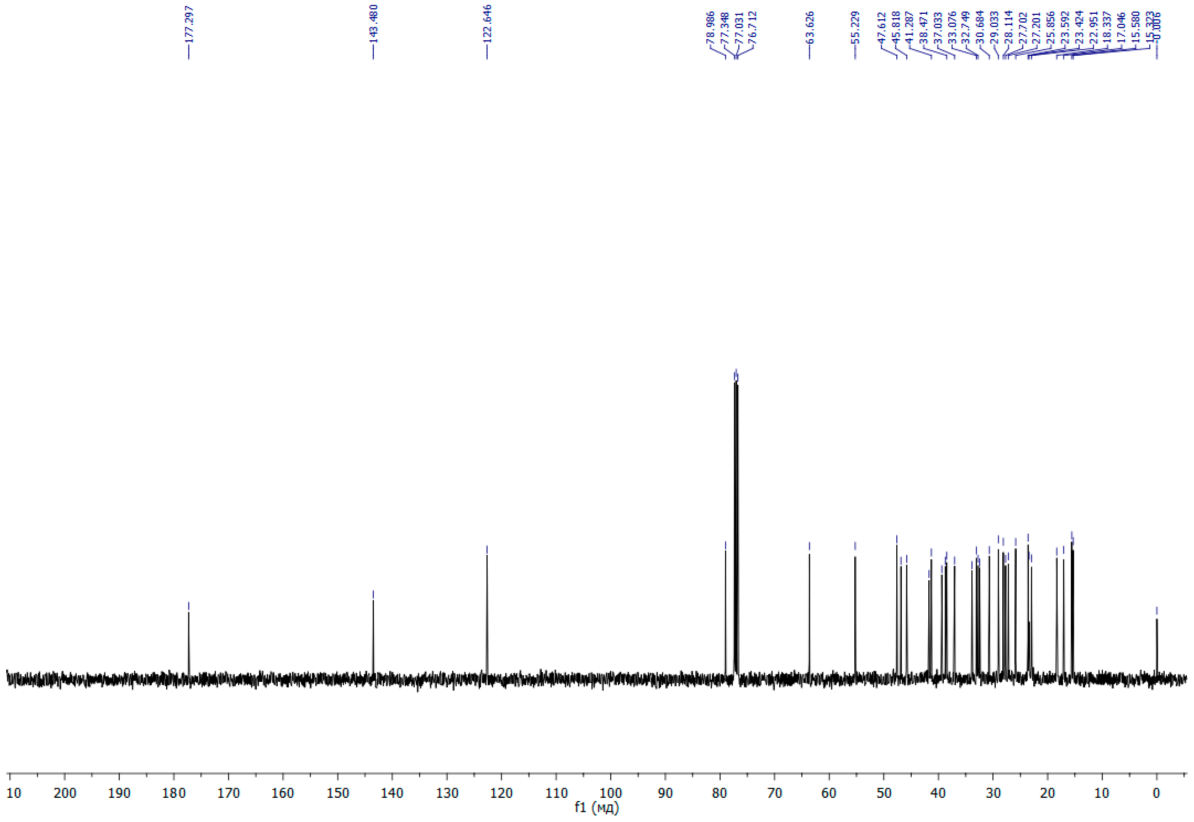

Figure S8. ^{1}H NMR Spectrum of compound **6a** (400 MHz, $CDCl_3$)

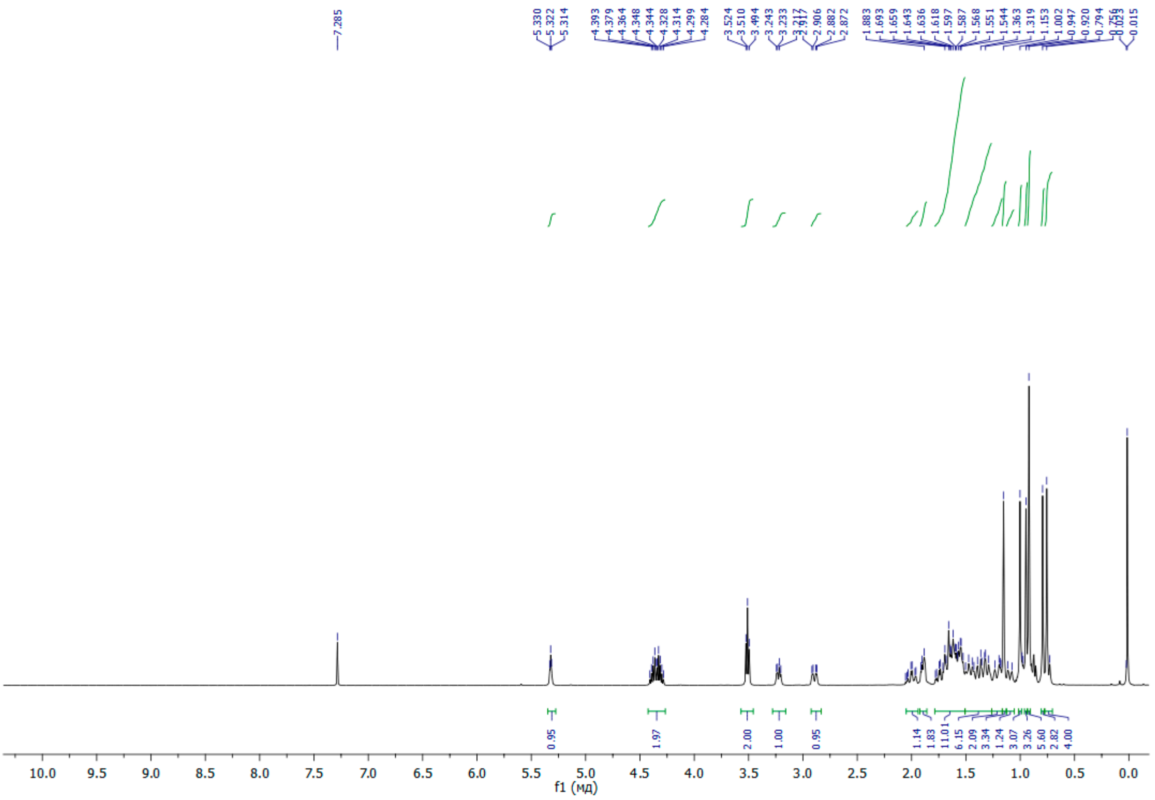

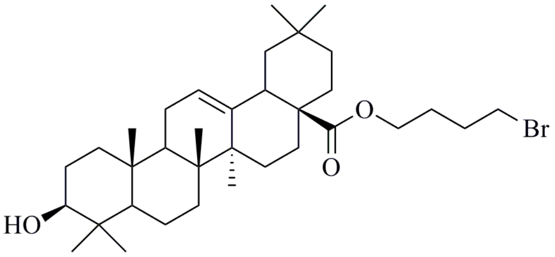

Figure S9. ^{13}C NMR Spectrum of compound **6b** (125 MHz, $CDCl_3$)

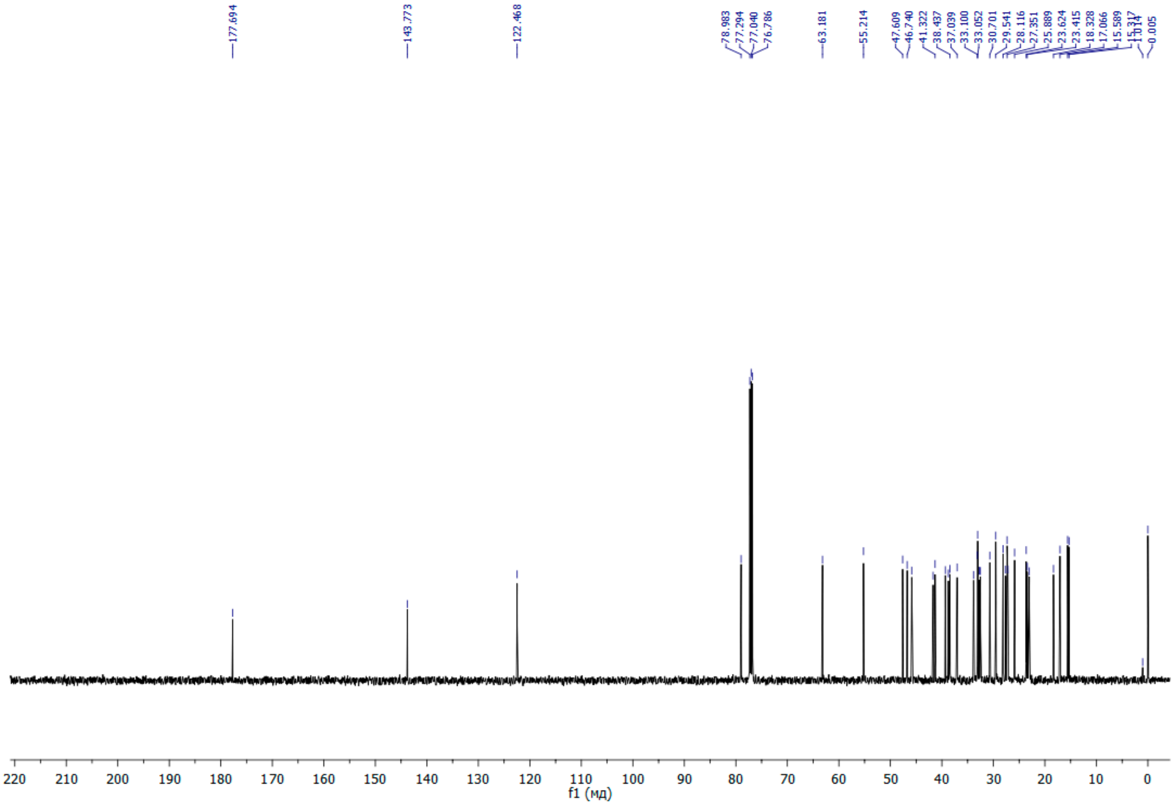

Figure S10. ^{1}H NMR Spectrum of compound **6b** (500 MHz, $CDCl_3$)

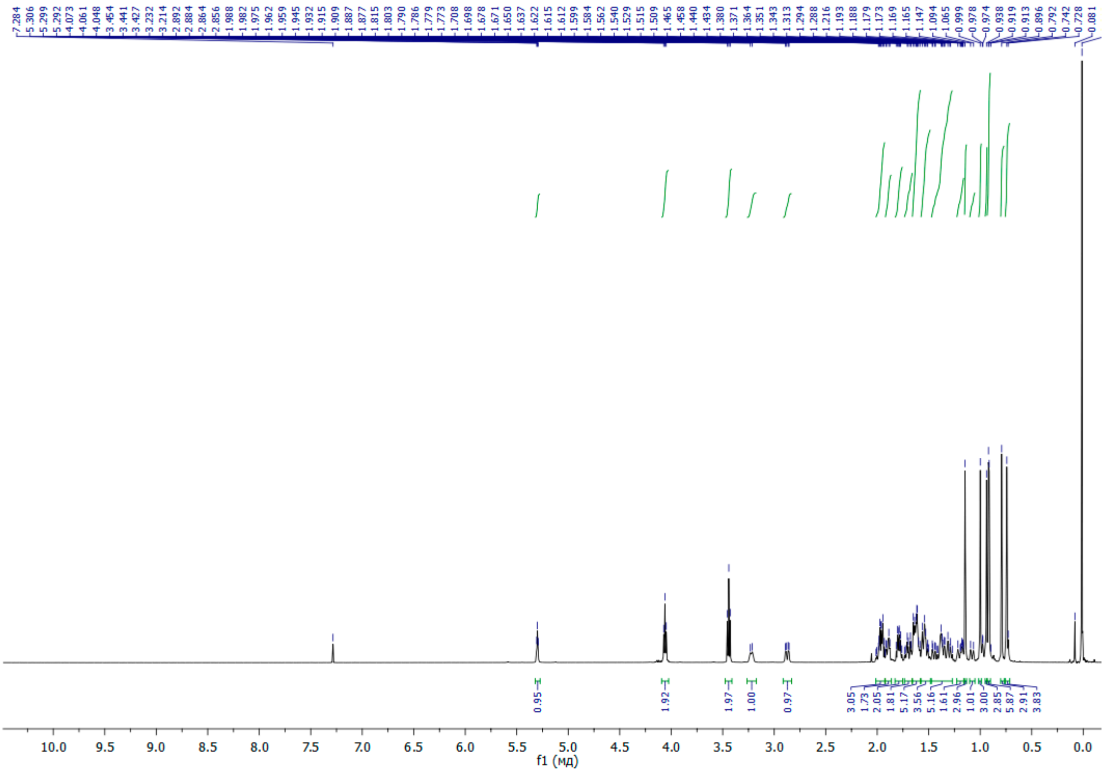

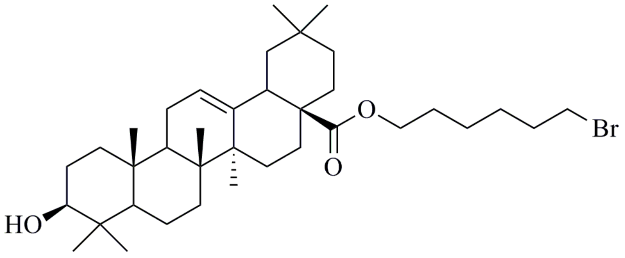

Figure S11. ^{13}C NMR Spectrum of compound **6c** (125 MHz, $CDCl_3$)

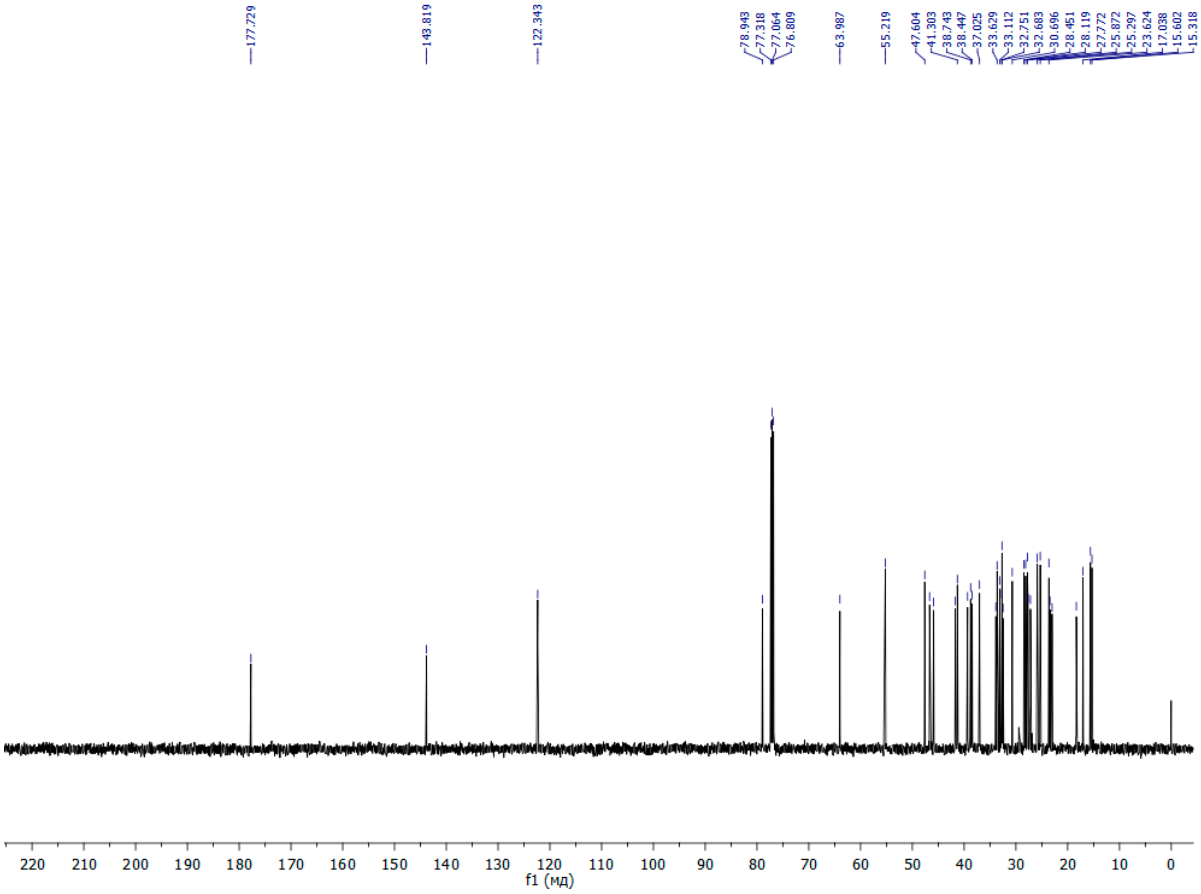

Figure S12. ^{1}H NMR Spectrum of compound **6c** (500 MHz, $CDCl_3$)

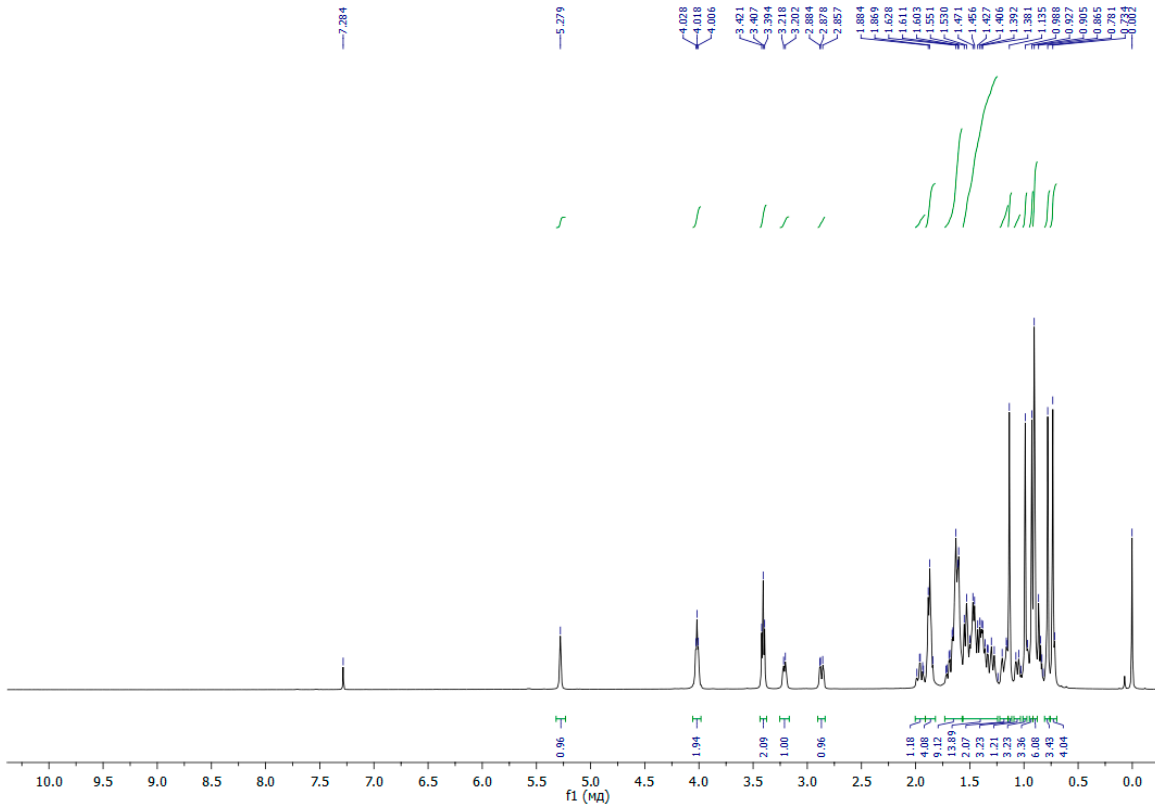

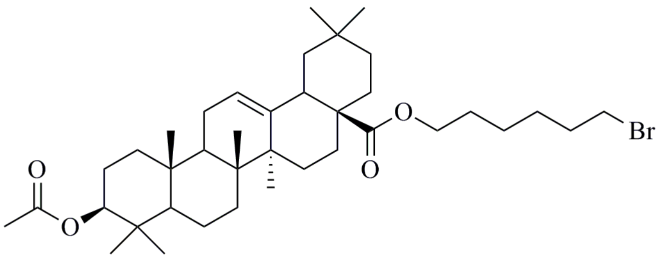

Figure S13. ^{13}C NMR Spectrum of compound **6d** (125 MHz, $CDCl_3$)

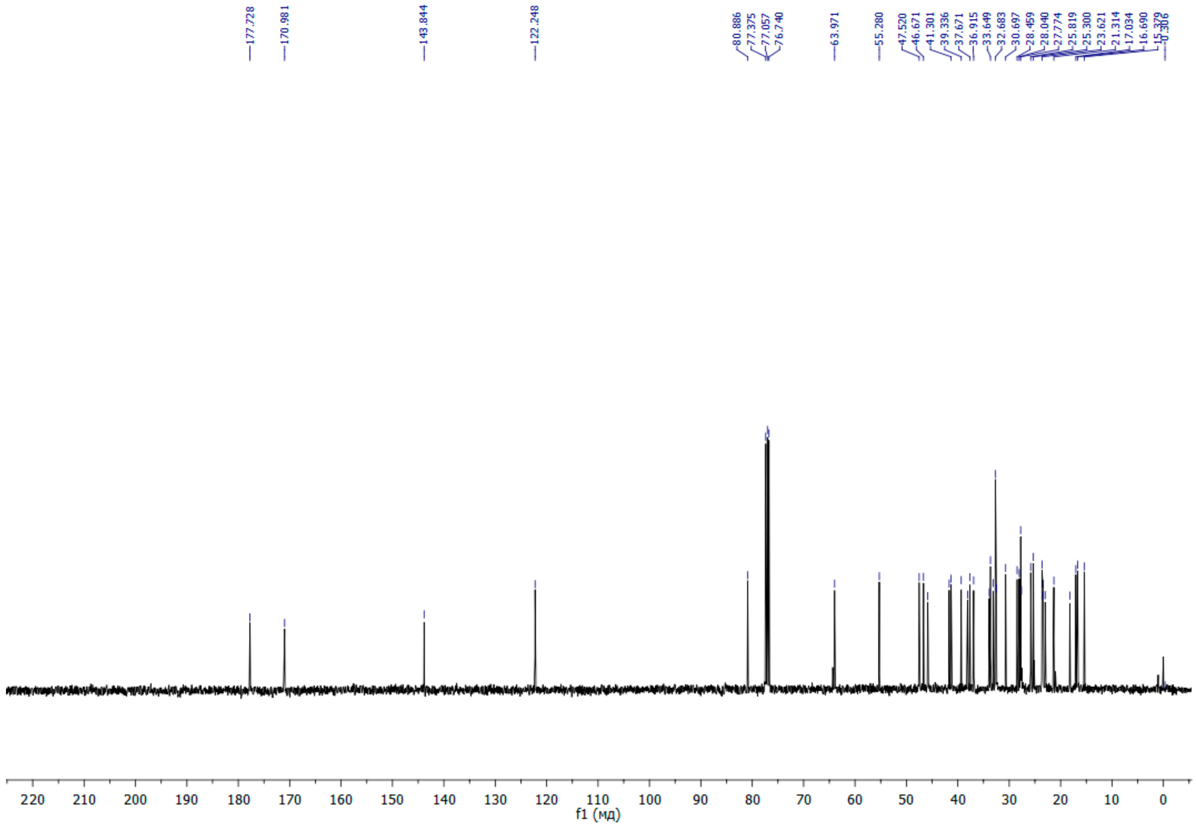

Figure S14. ^{1}H NMR Spectrum of compound **6d** (500 MHz, $CDCl_3$)

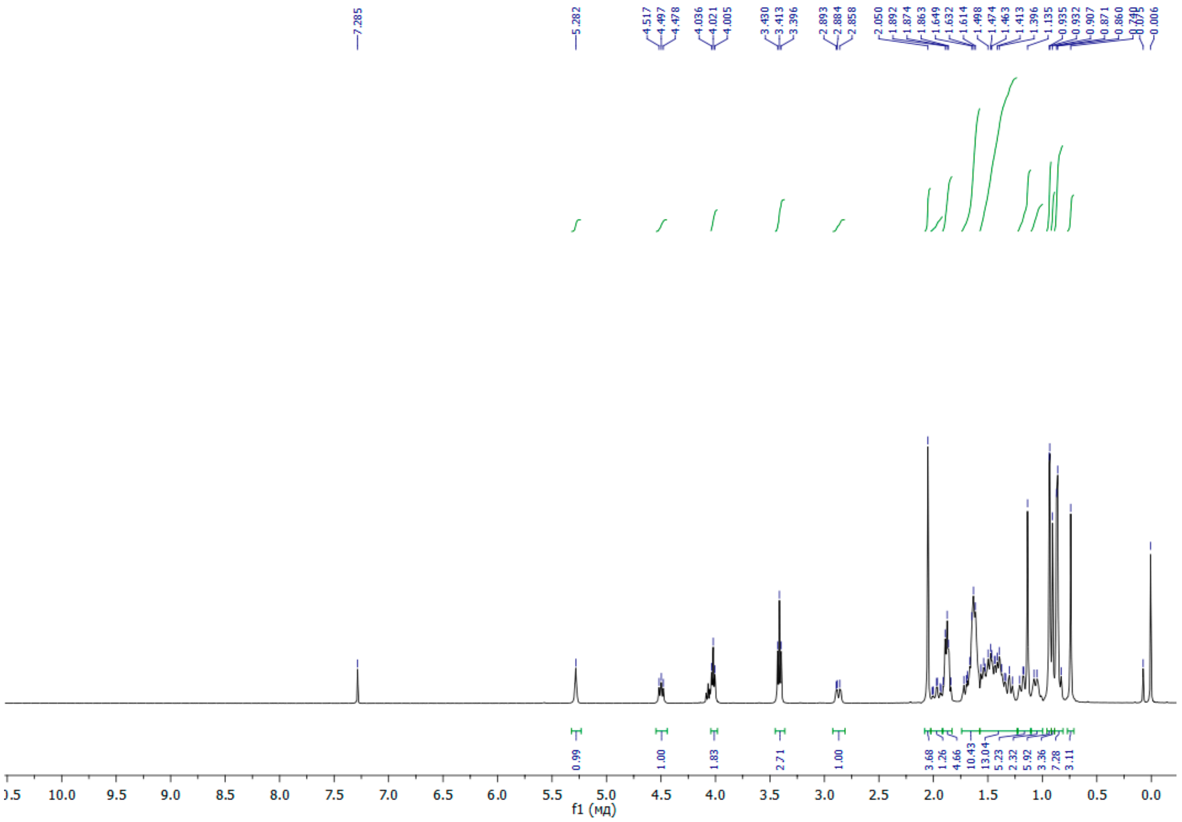

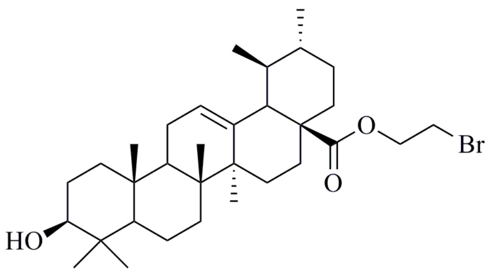

Figure S15. ^{13}C NMR Spectrum of compound **7a** (100 MHz, $CDCl_3$)

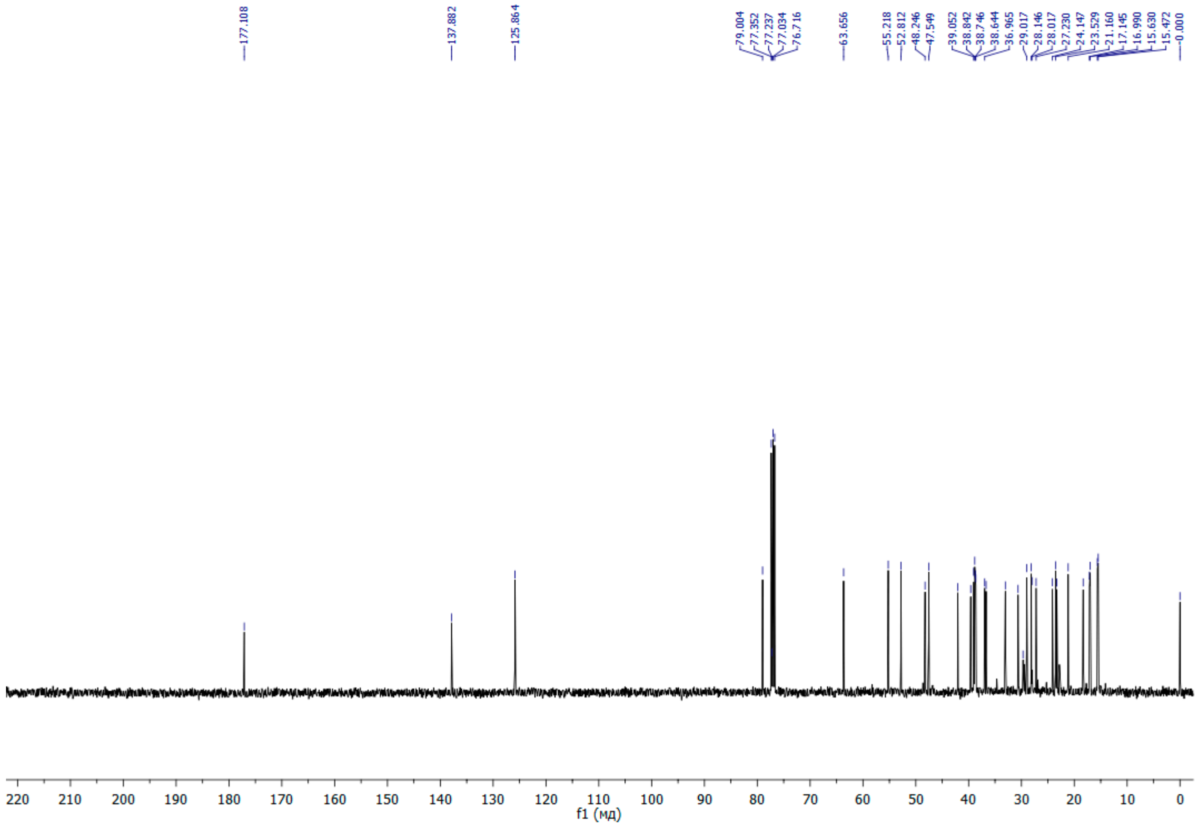

Figure S16. ^{1}H NMR Spectrum of compound **7a** (400 MHz, $CDCl_3$)

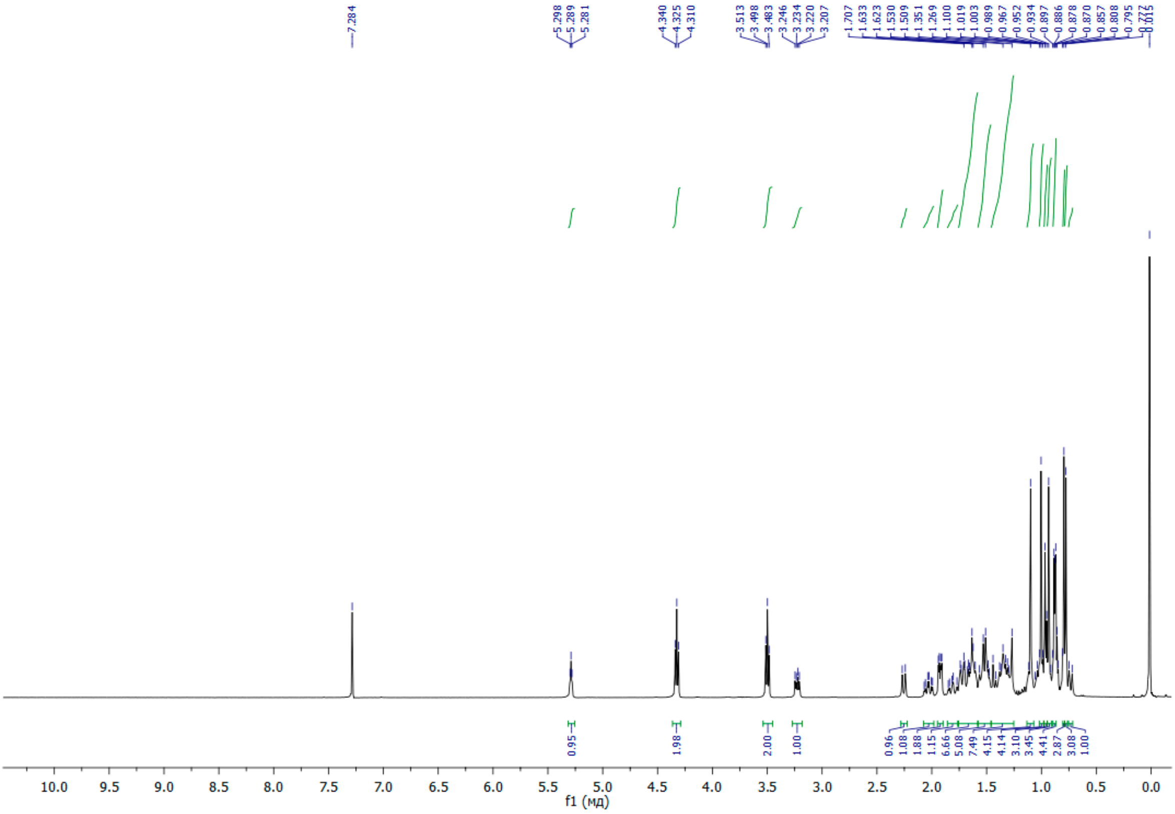

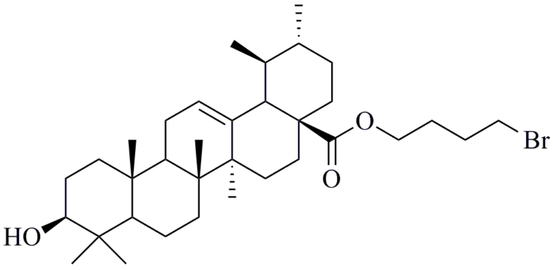

Figure S17. ^{13}C NMR Spectrum of compound **7b** (125 MHz, $CDCl_3$)

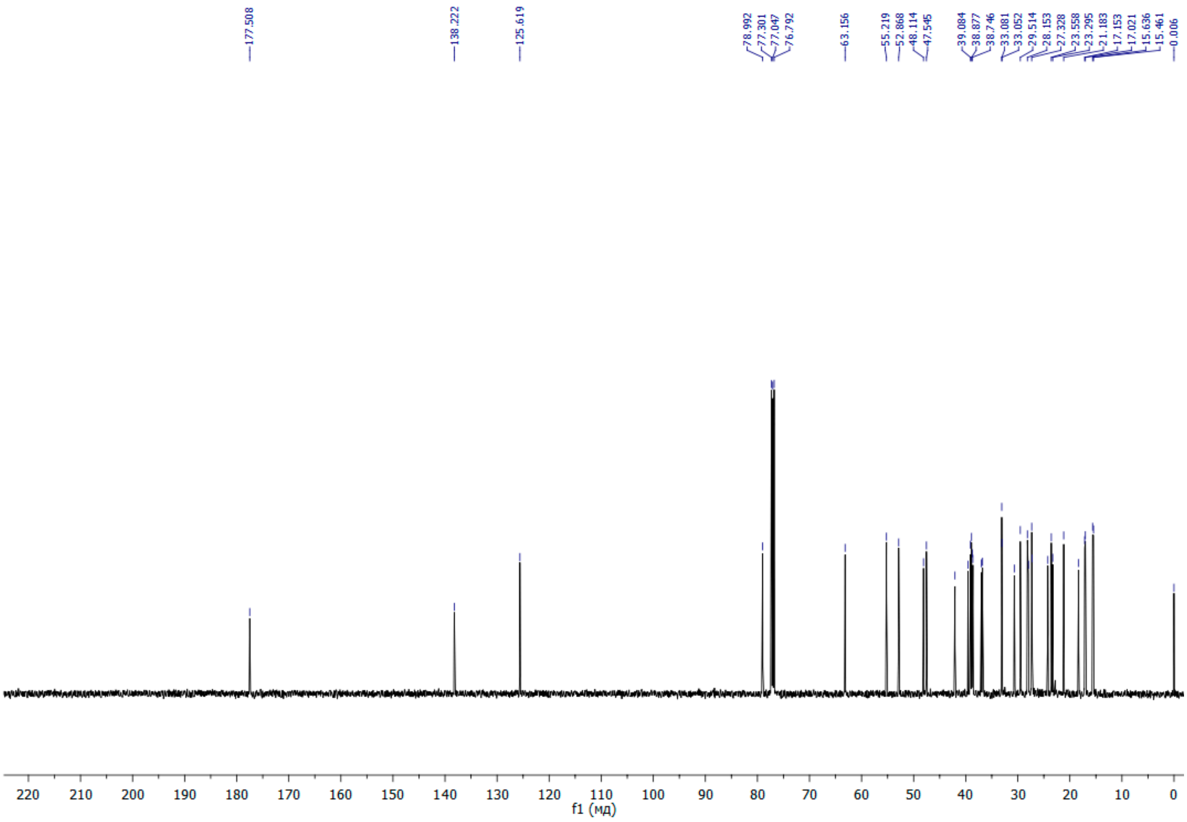

Figure S18. ^1H NMR Spectrum of compound **7b** (500 MHz, $CDCl_3$)

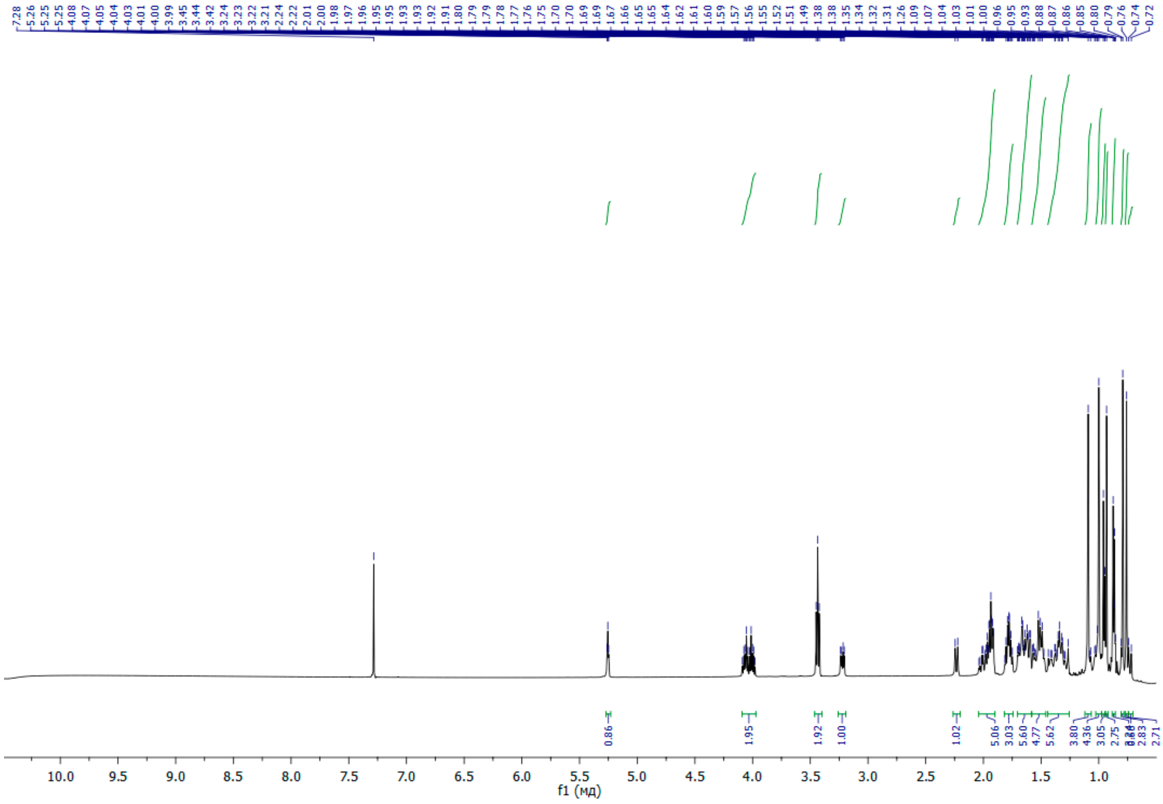

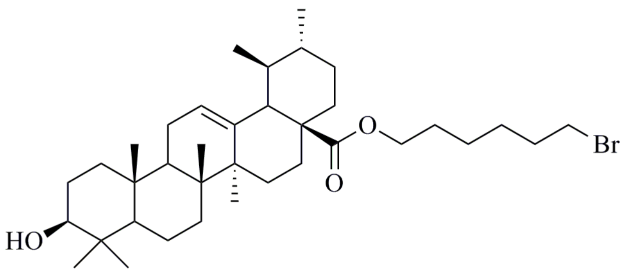

Figure S19. ^{13}C NMR Spectrum of compound **7c** (125 MHz, $CDCl_3$)

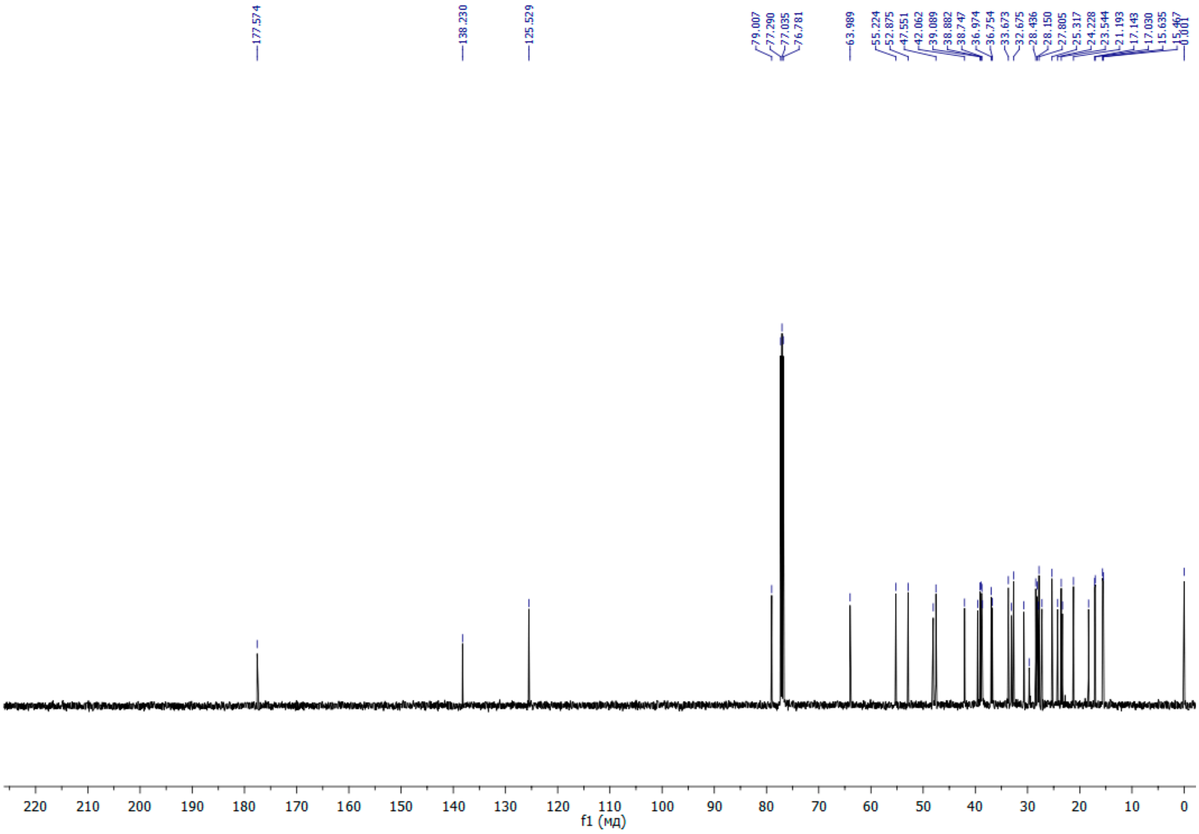

Figure S20. ^{1}H NMR Spectrum of compound **7c** (500 MHz, $CDCl_3$)

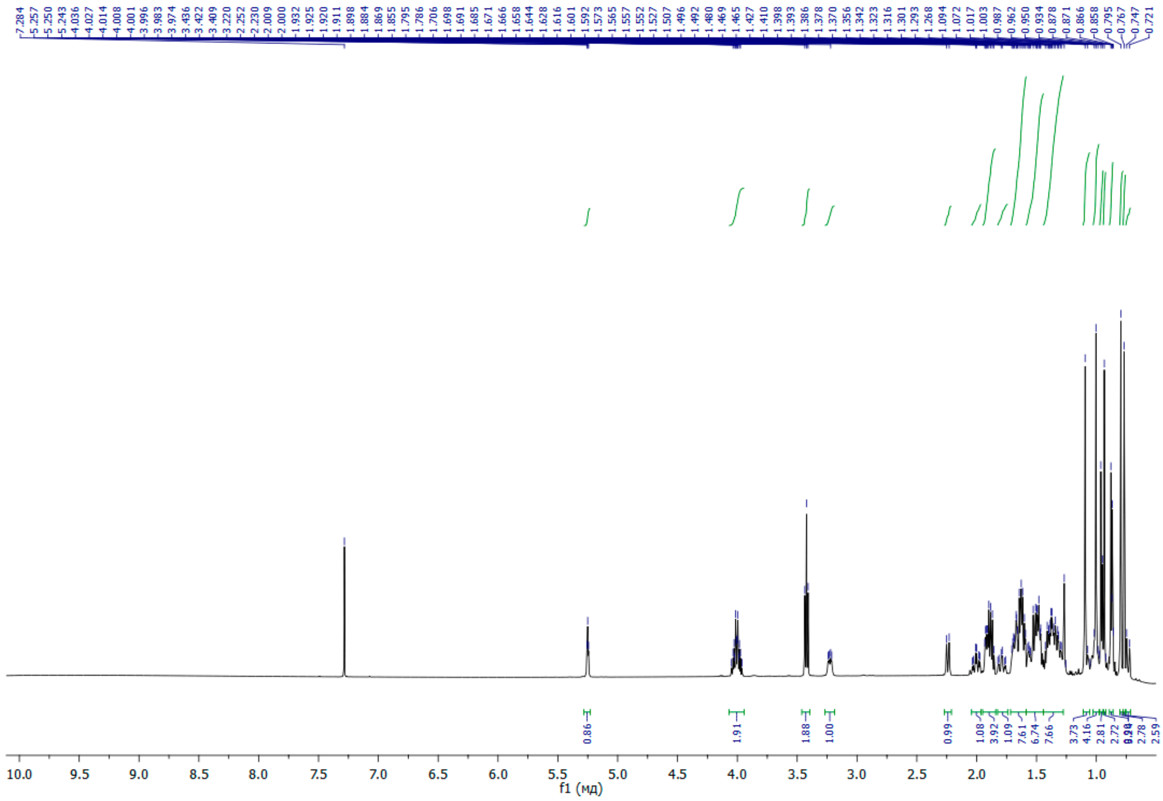

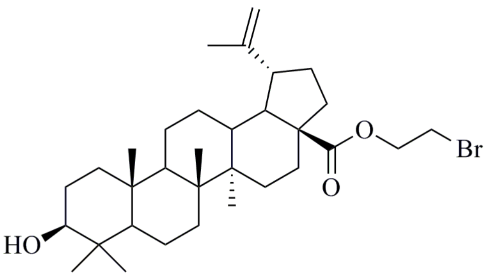

Figure S21. ^{13}C NMR Spectrum of compound **8a** (125 MHz, $CDCl_3$)

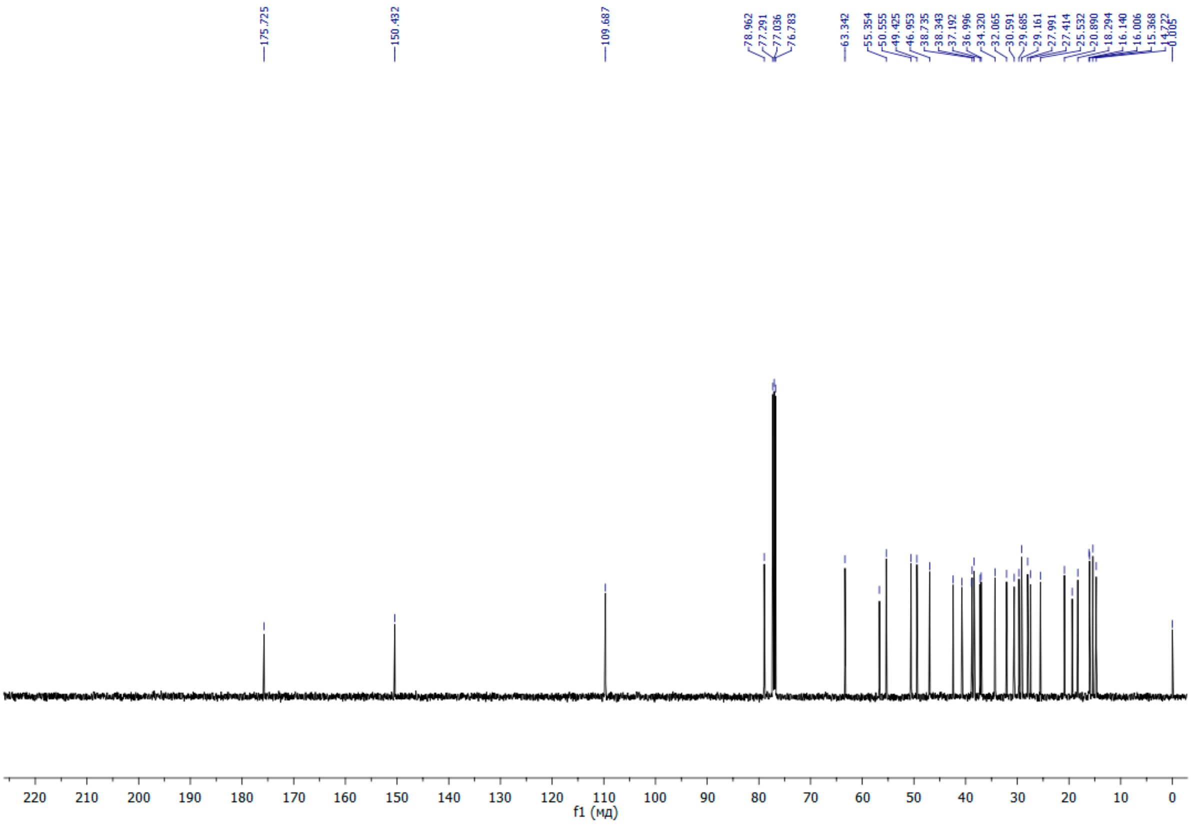

Figure S22. ^{1}H NMR Spectrum of compound **8a** (500 MHz, $CDCl_3$)

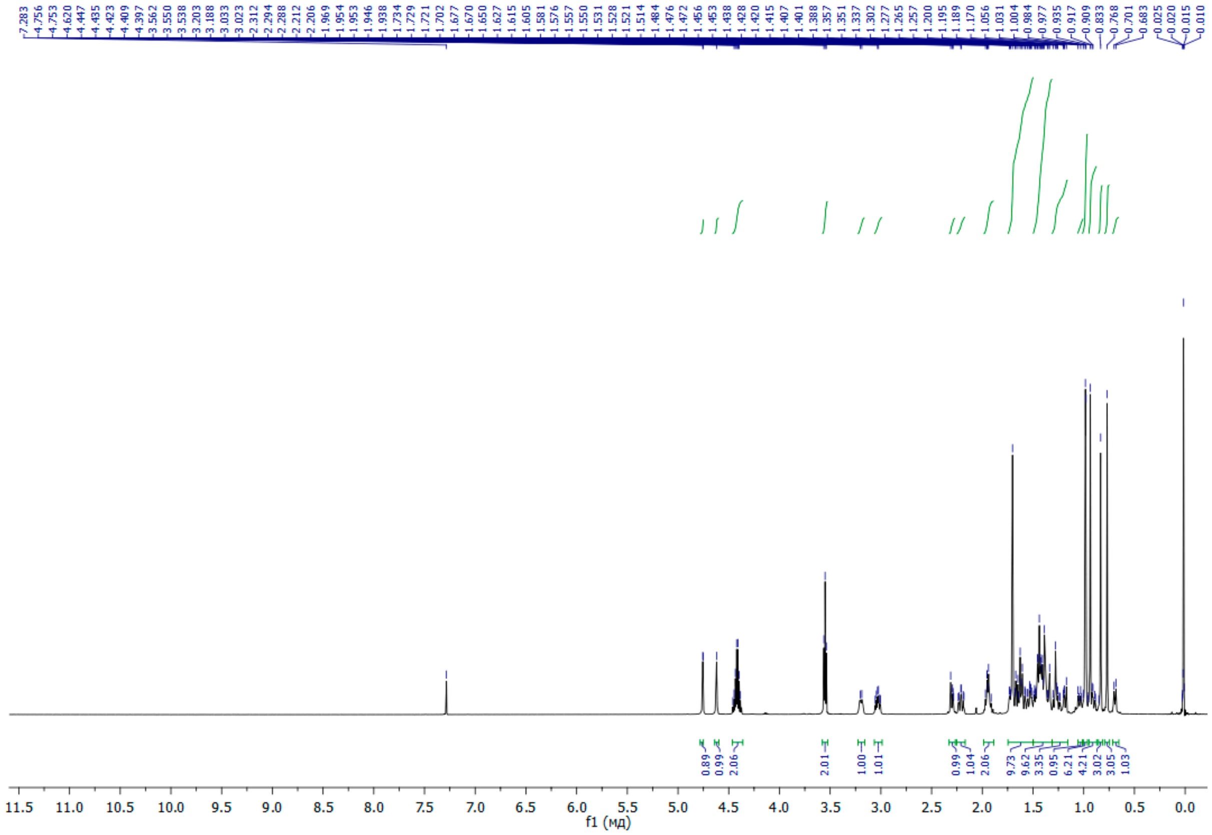

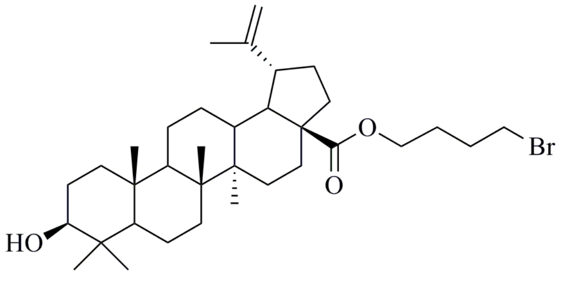

Figure S23. ^{13}C NMR Spectrum of compound **8b** (100 MHz, $CDCl_3$)

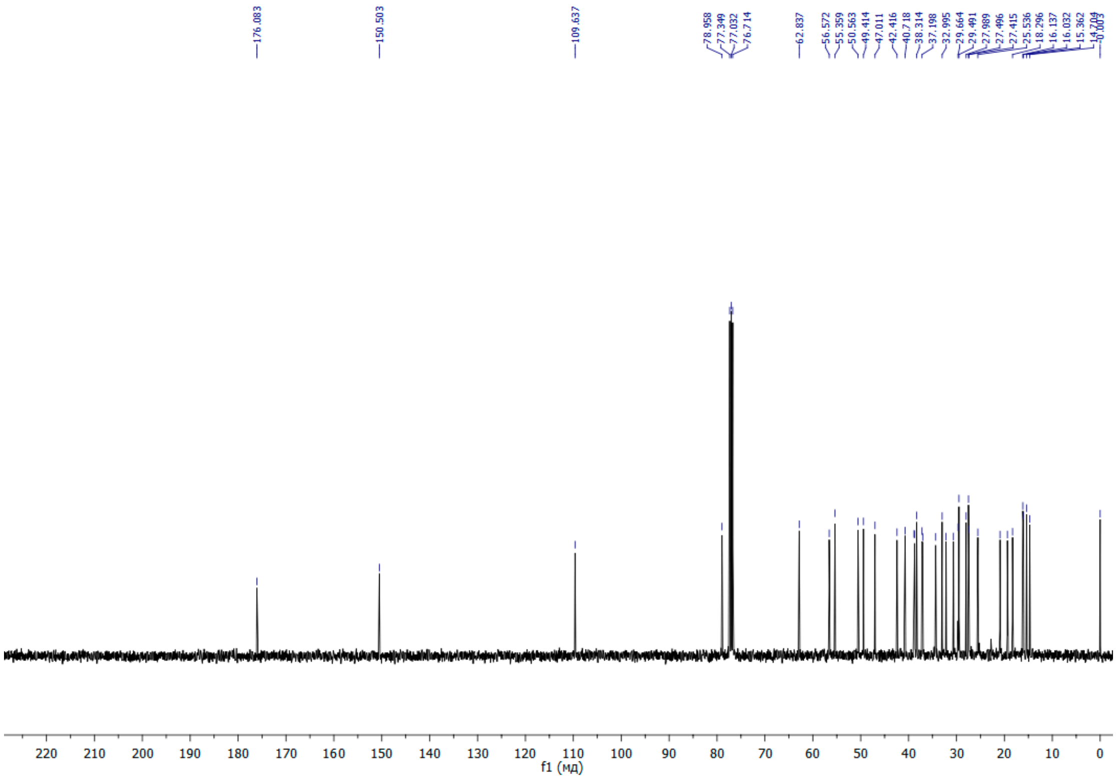

Figure S24. ^{1}H NMR Spectrum of compound **8b** (400 MHz, $CDCl_3$)

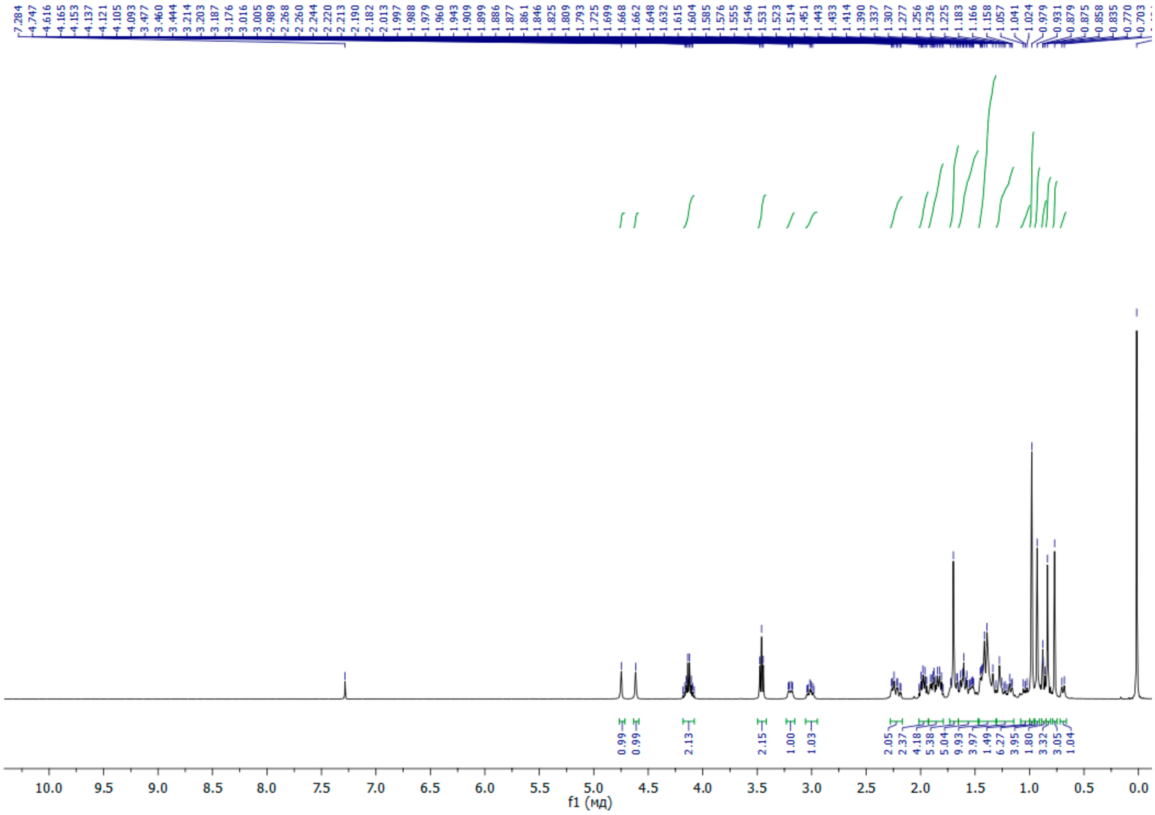

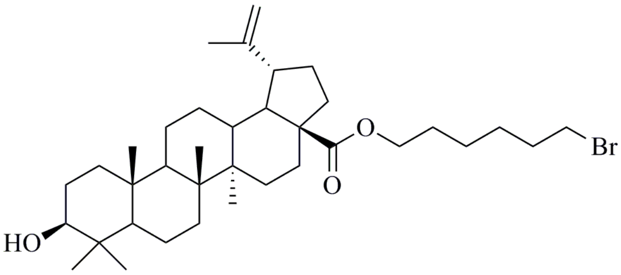

Figure S25. ^{13}C NMR Spectrum of compound **8c** (125 MHz, $CDCl_3$)

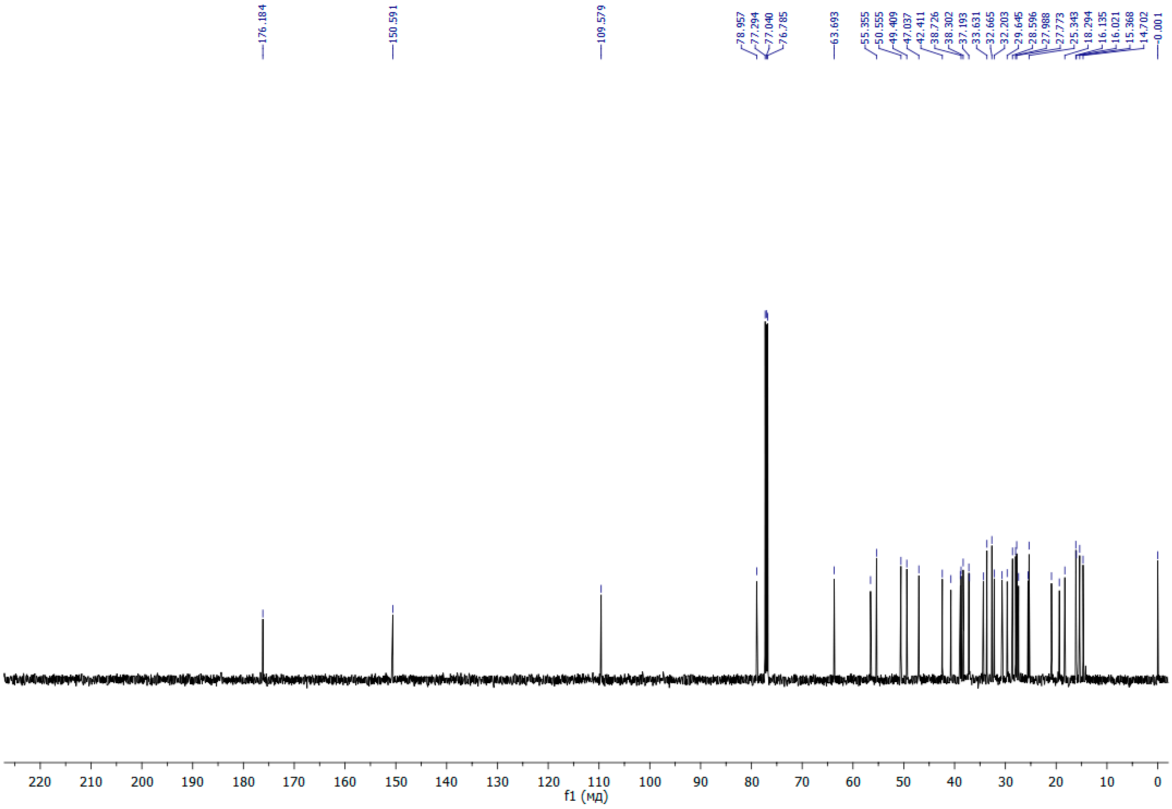

Figure S26. ^{1}H NMR Spectrum of compound **8c** (500 MHz, $CDCl_3$)

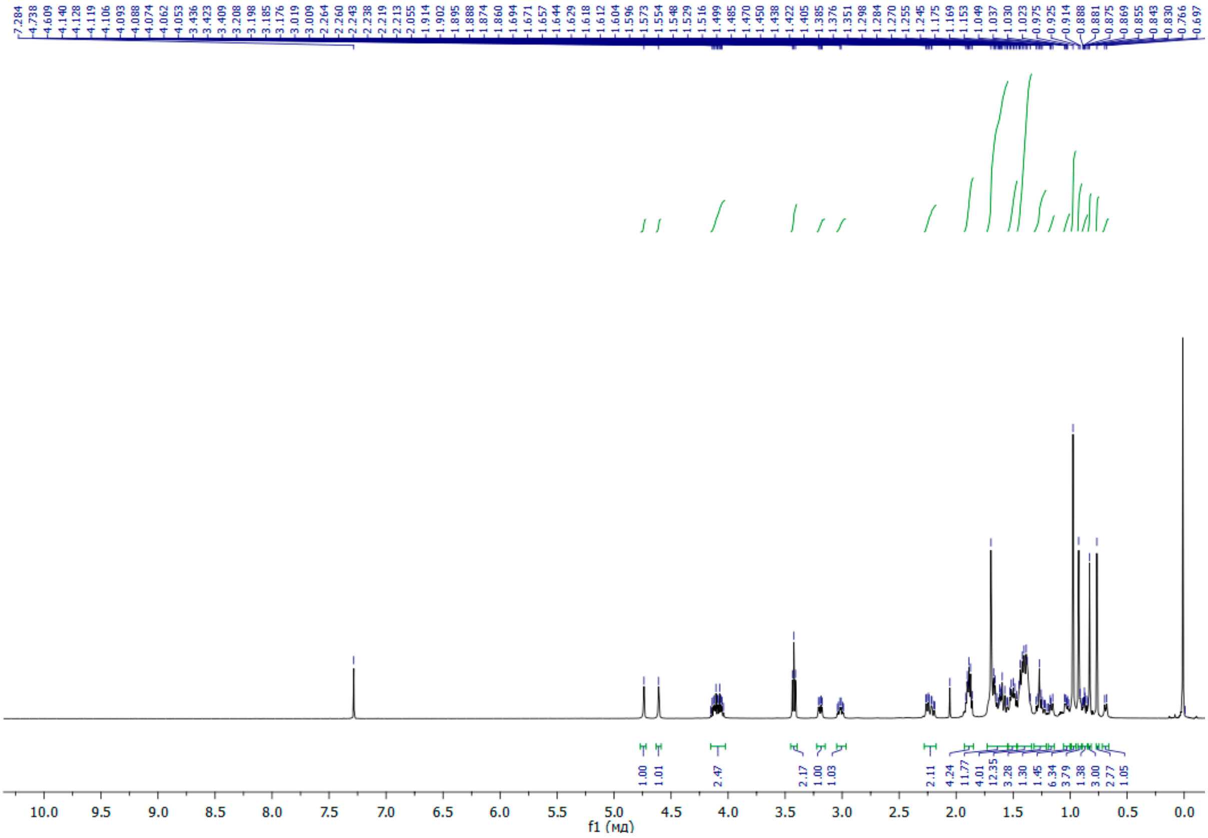

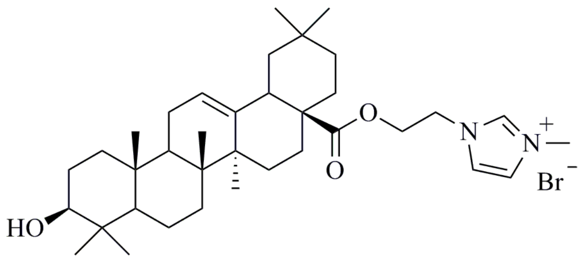

Figure S27. ^{13}C NMR Spectrum of compound **9a** (100 MHz, DMSO-d$_6$)

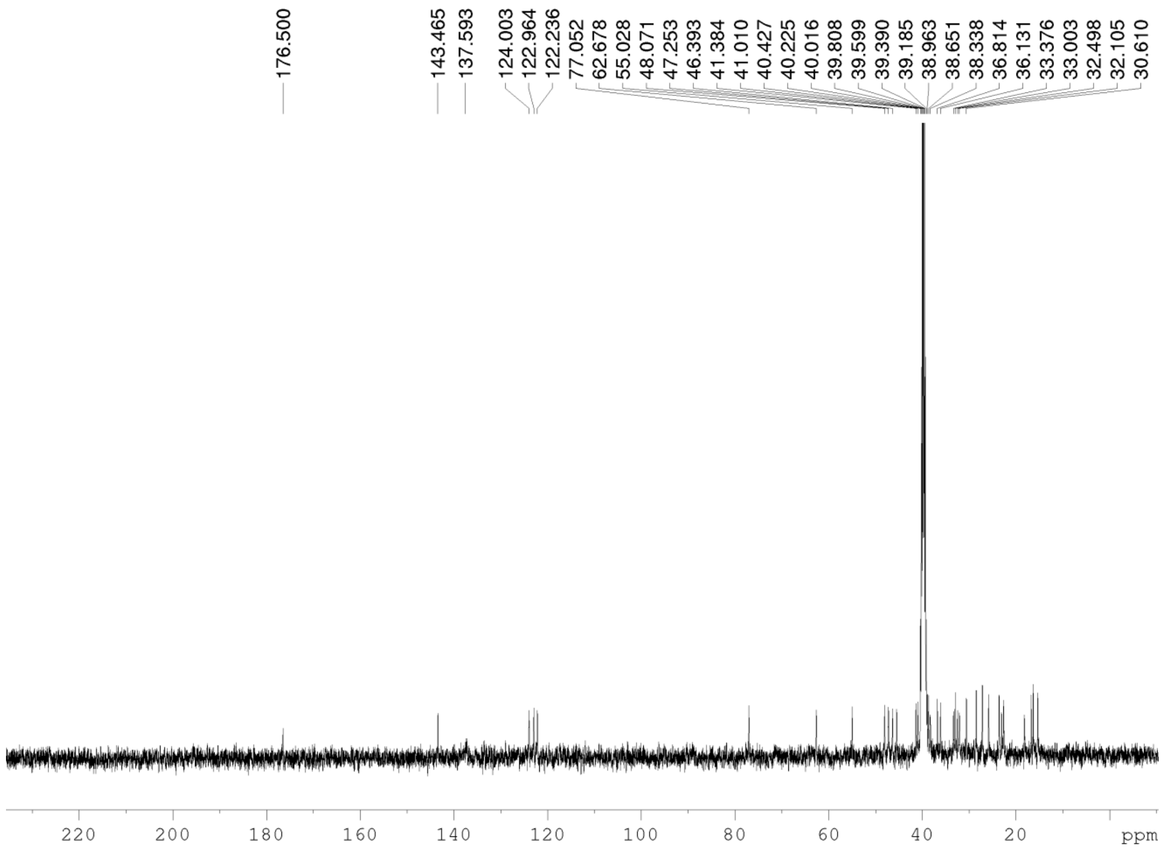

Figure S28. ^{1}H NMR Spectrum of compound **9a** (400 MHz, DMSO-d$_6$)

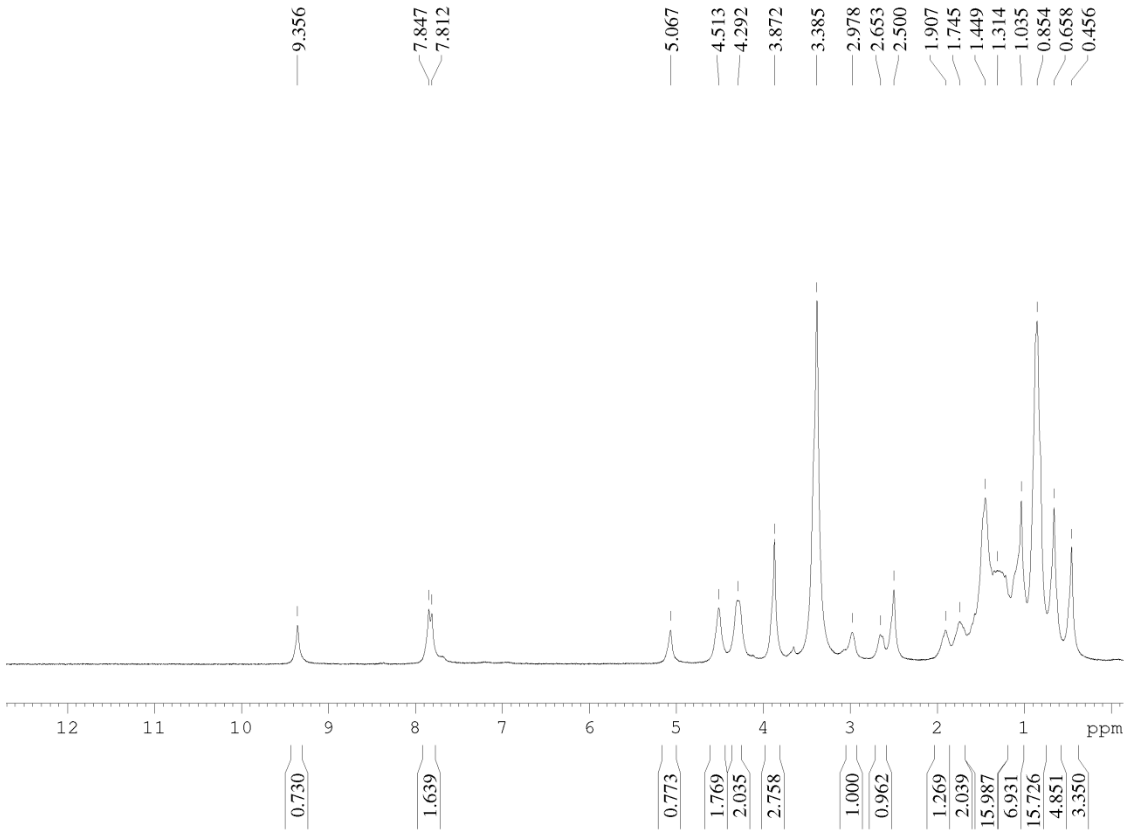

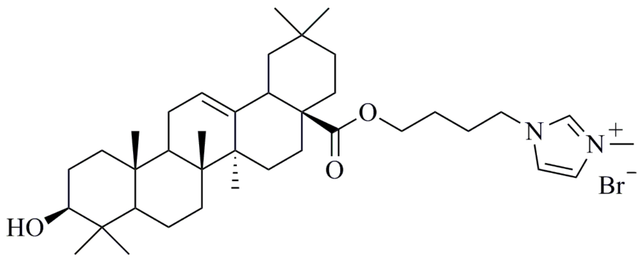

Figure S29. ^{13}C NMR Spectrum of compound **9b** (125 MHz, DMSO-d$_6$)

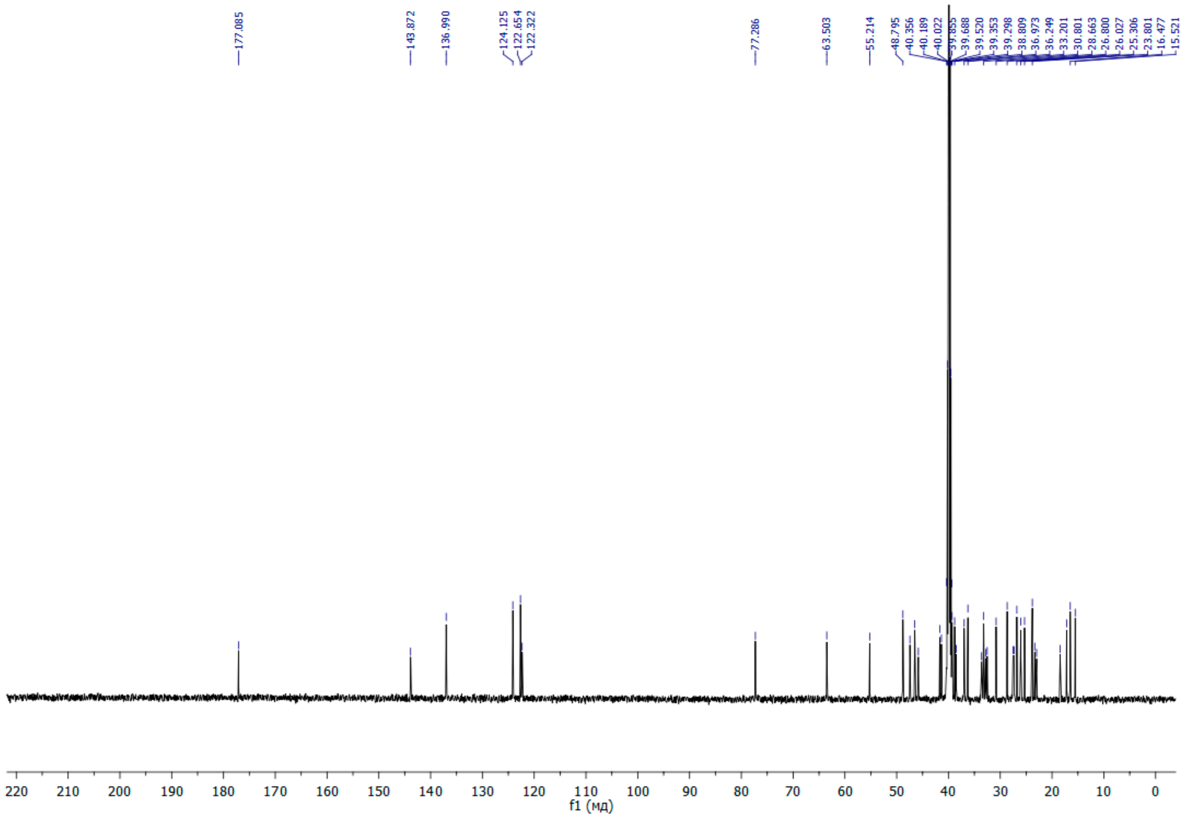

Figure S30. ^{1}H NMR Spectrum of compound **9b** (500 MHz, DMSO-d$_6$)

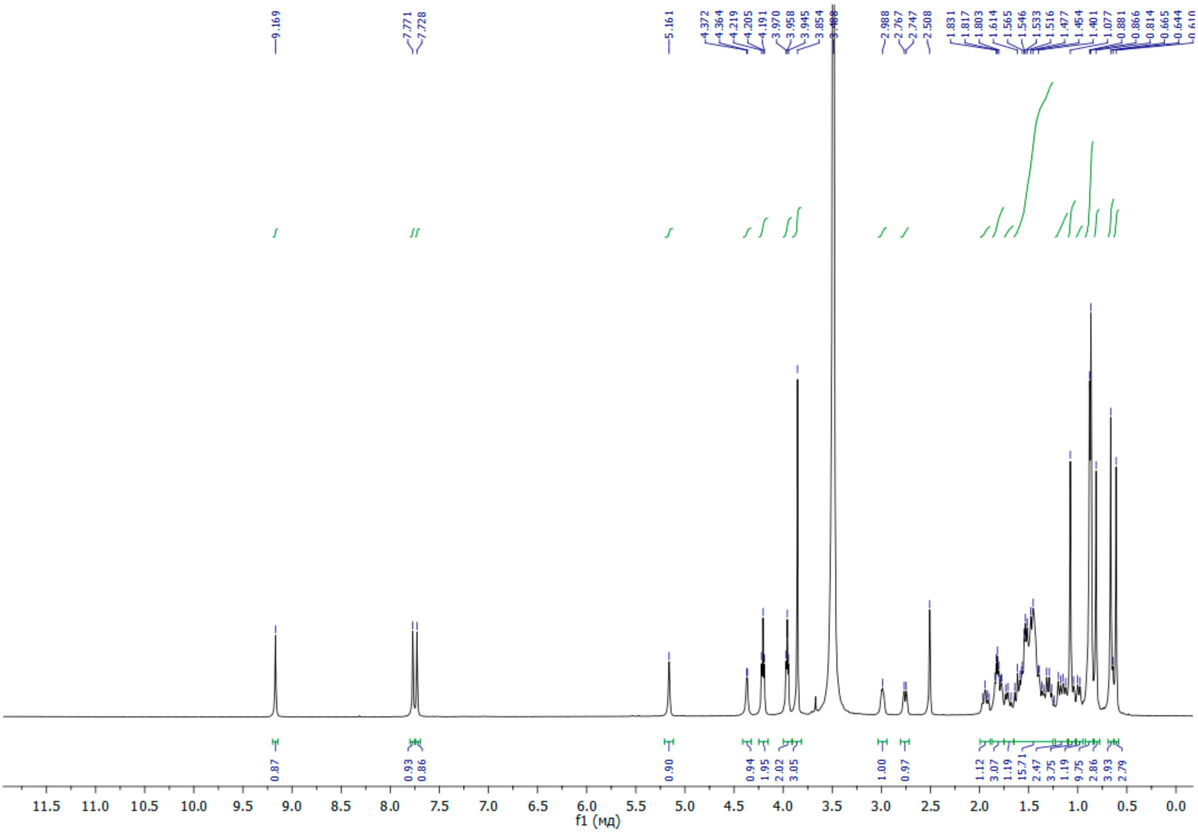

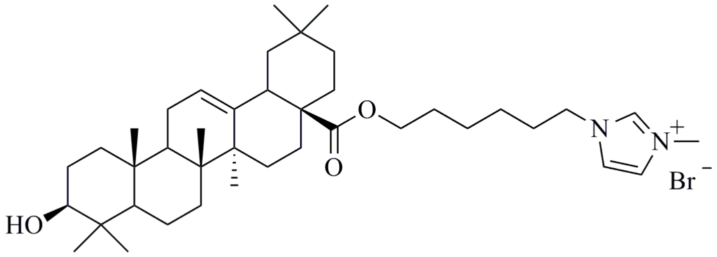

Figure S31. ^{13}C NMR Spectrum of compound **9c** (125 MHz, DMSO-d_6)

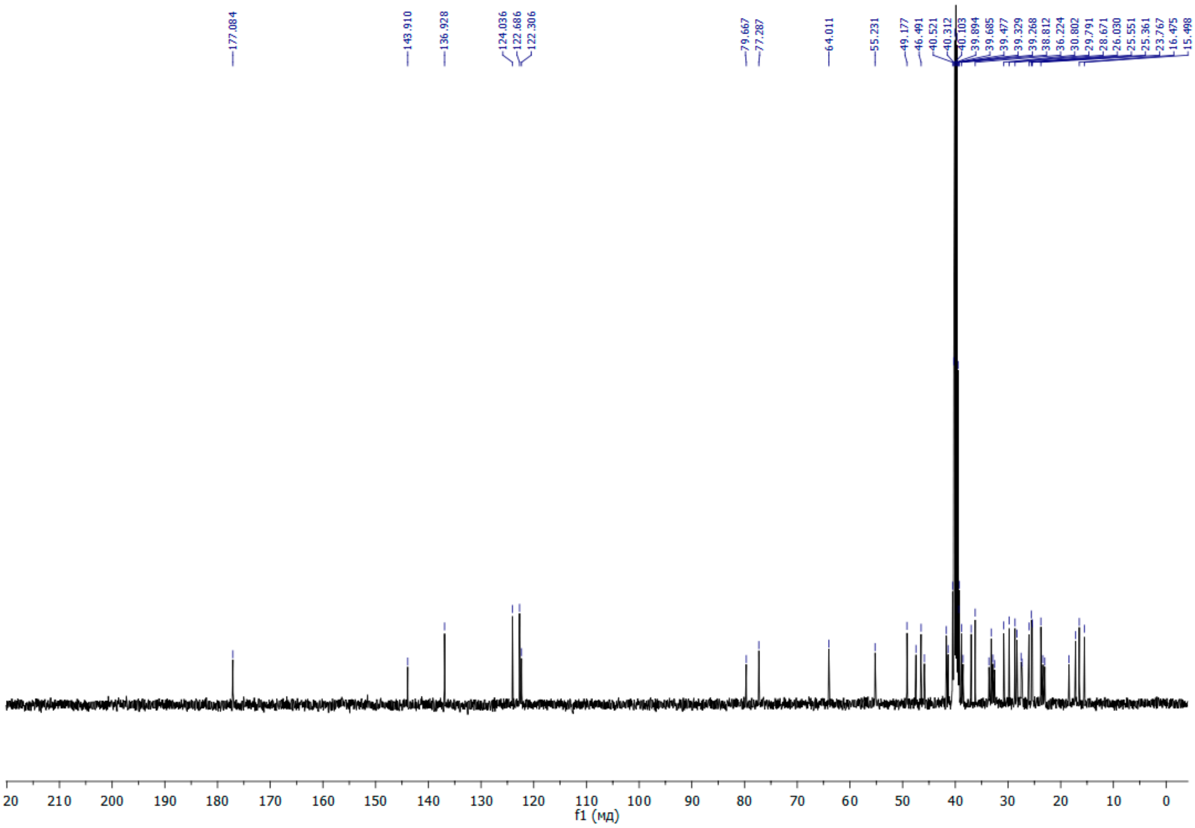

Figure S32. ^{1}H NMR Spectrum of compound **9c** (500 MHz, DMSO-d_6)

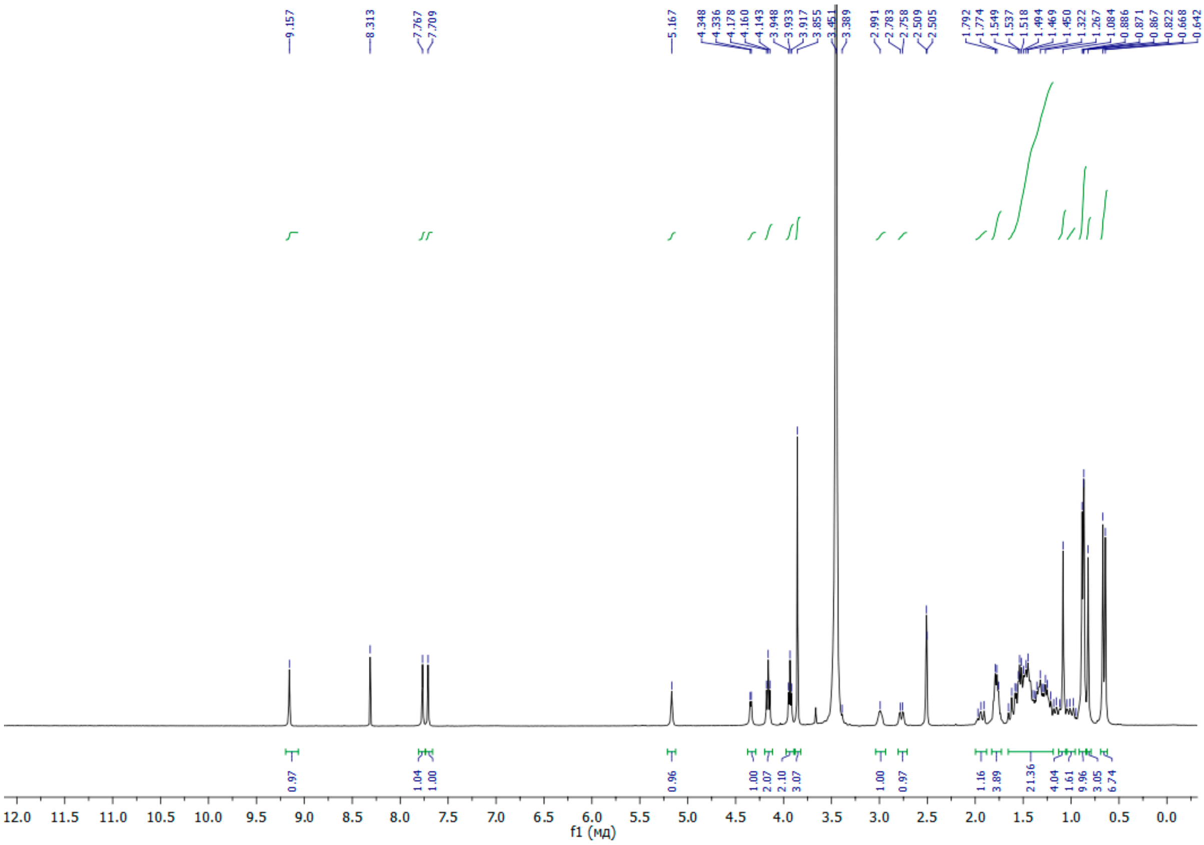

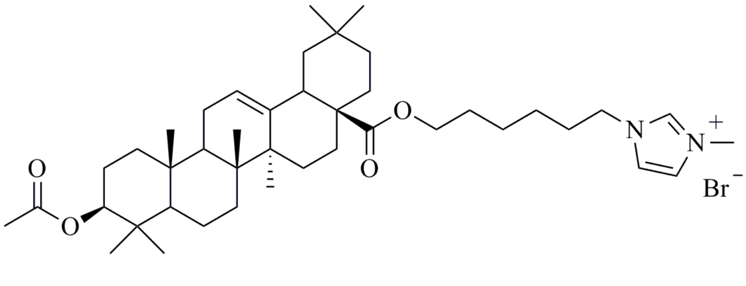

Figure S33. ^{13}C NMR Spectrum of compound **9d** (100 MHz, DMSO-d_6)

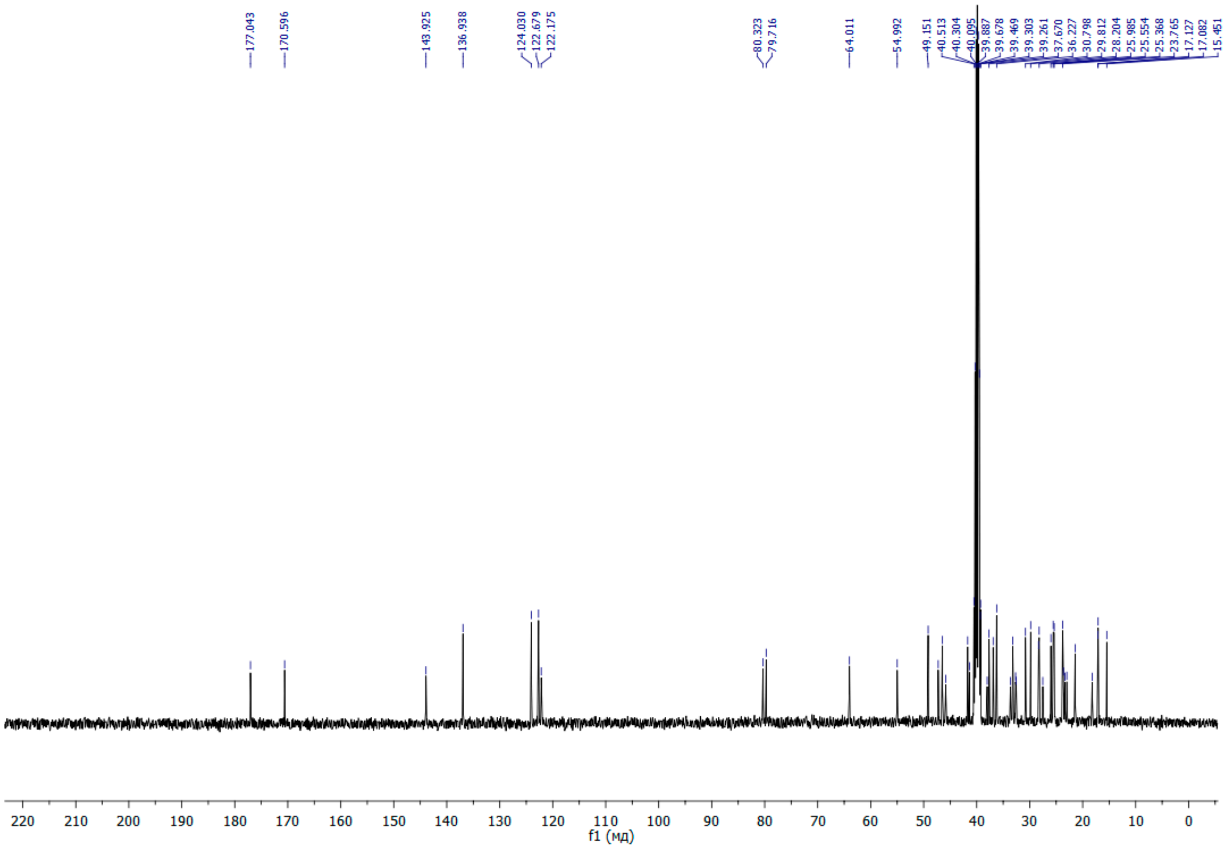

Figure S34. ^{1}H NMR Spectrum of compound **9d** (400 MHz, DMSO-d_6)

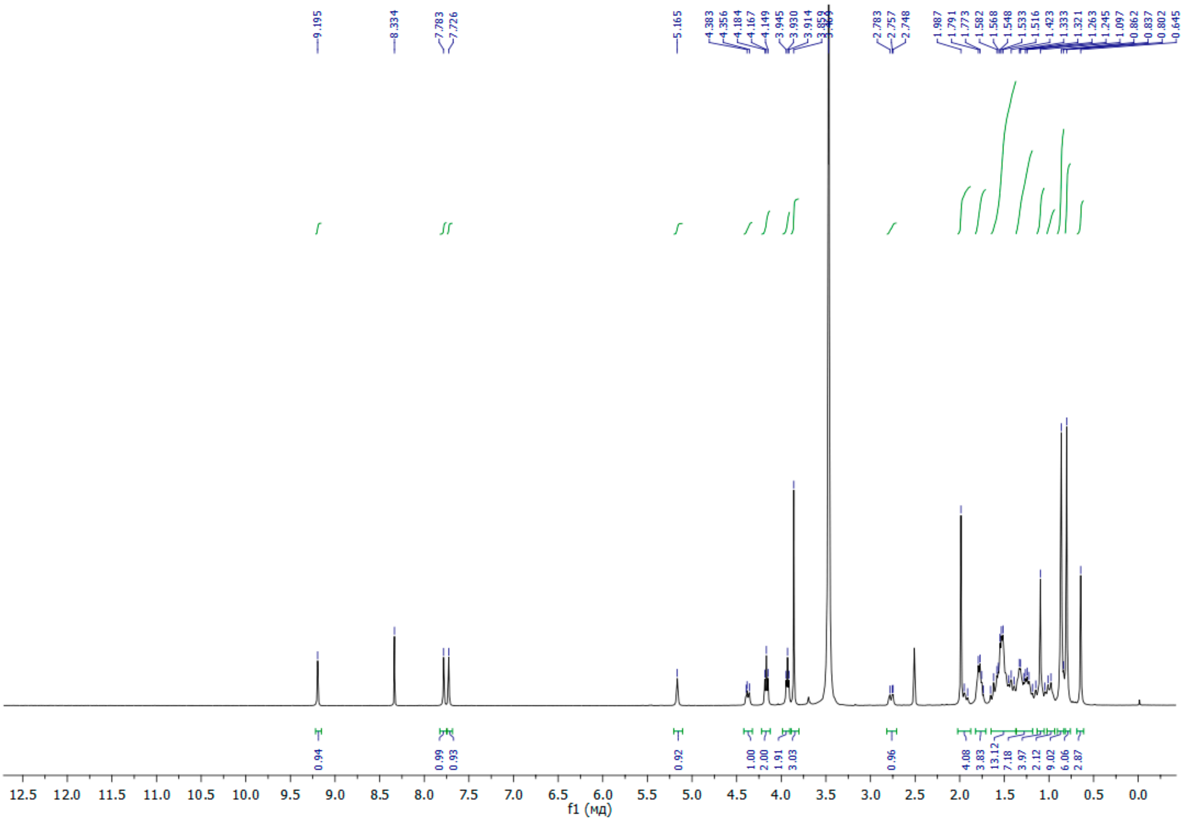

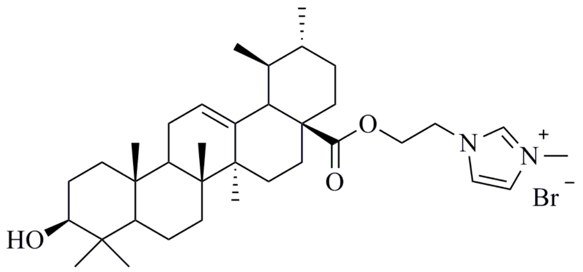

Figure S35. ^{13}C NMR Spectrum of compound **10a** (100 MHz, DMSO-d$_6$)

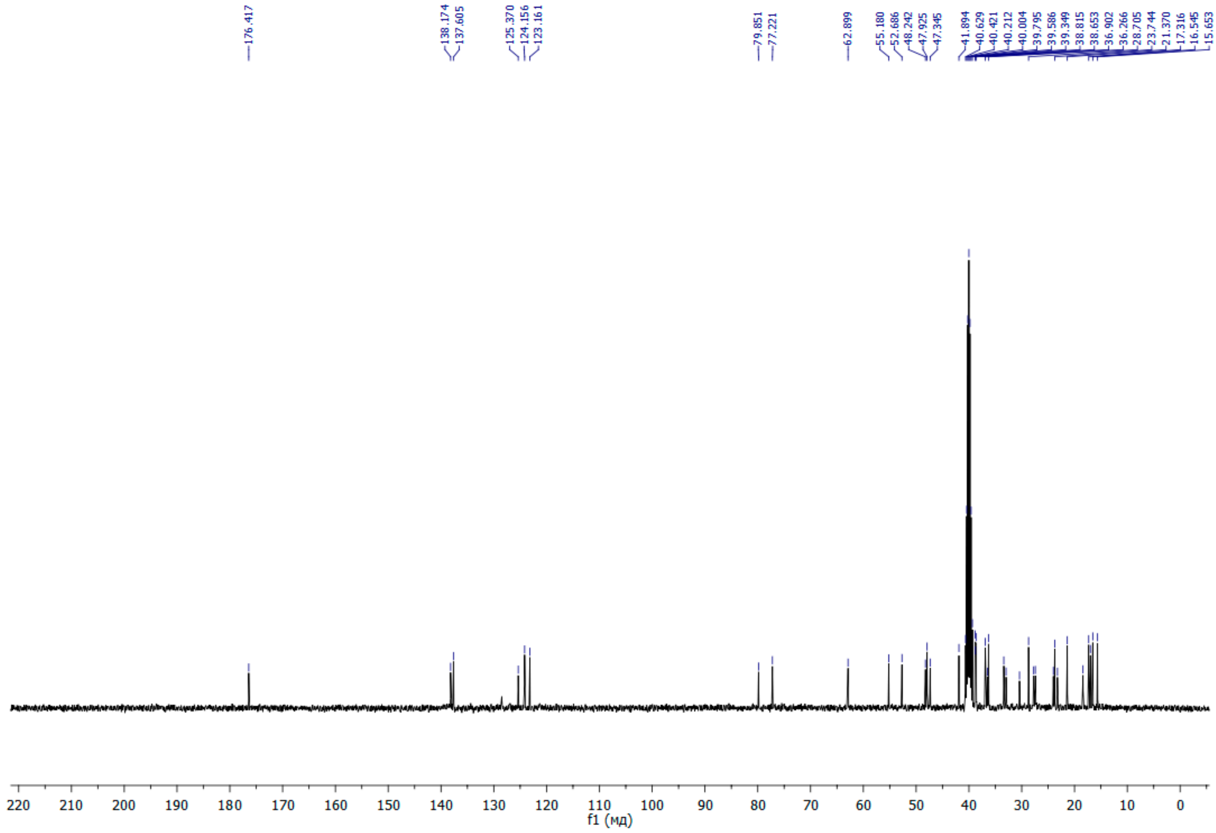

Figure S36. ^{1}H NMR Spectrum of compound **10a** (400 MHz, DMSO-d_6)

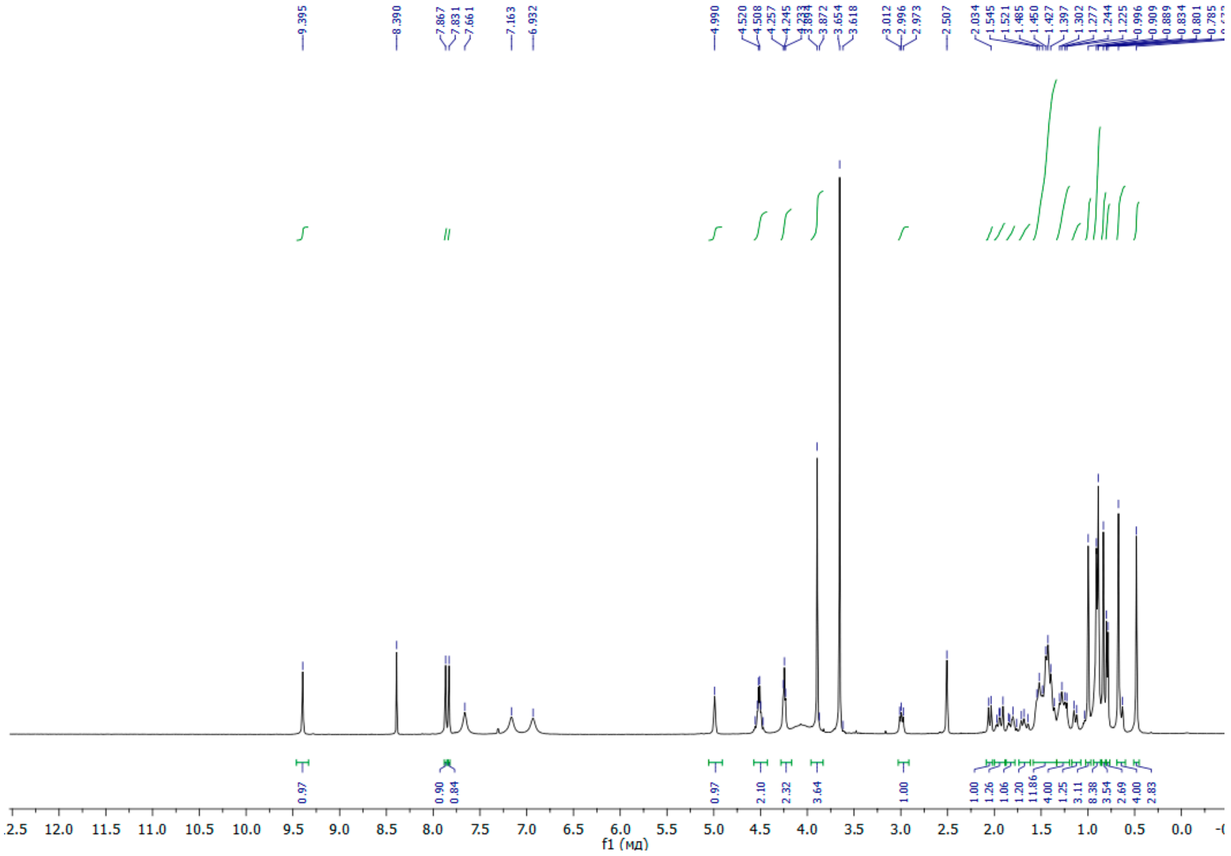

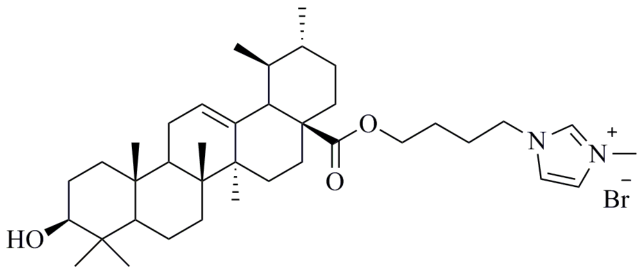

Figure S37. ^{13}C NMR Spectrum of compound **10b** (100 MHz, DMSO-d_6)

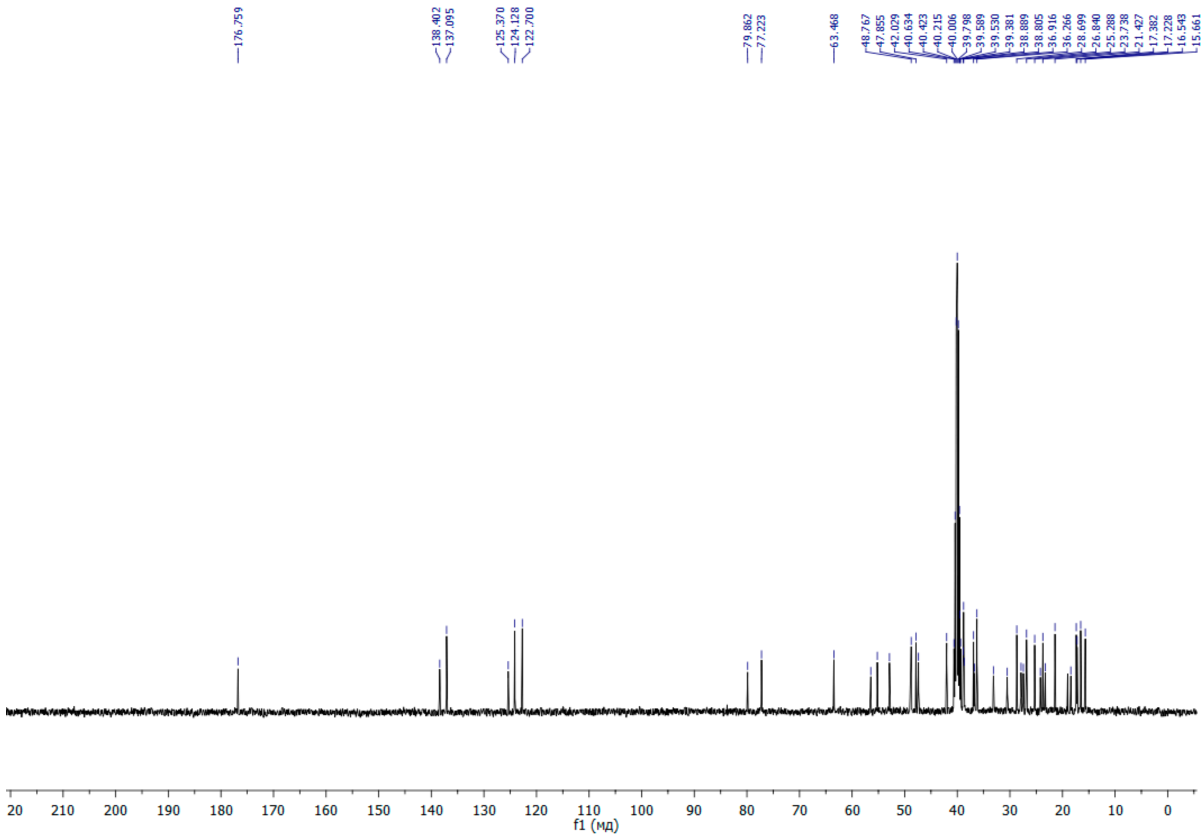

Figure S38. ^{1}H NMR Spectrum of compound **10b** (400 MHz, DMSO-d_6)

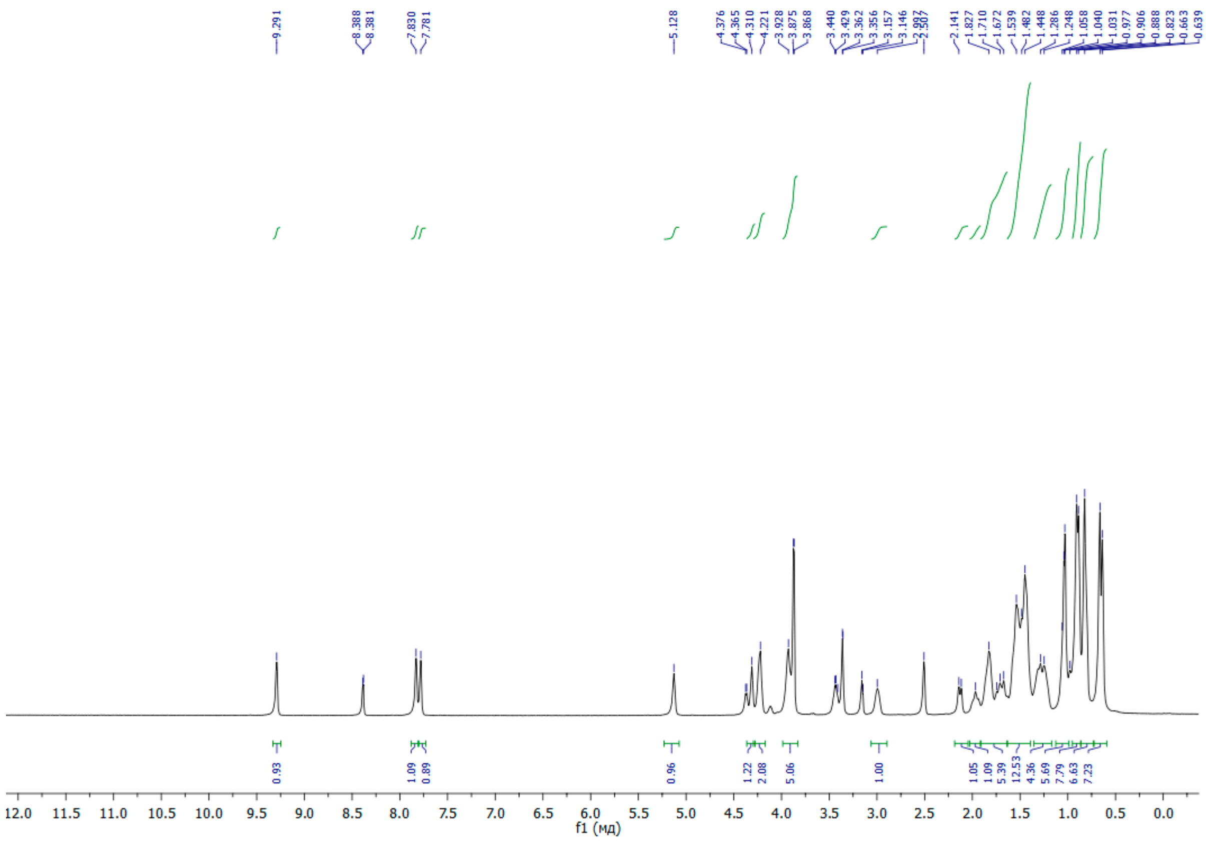

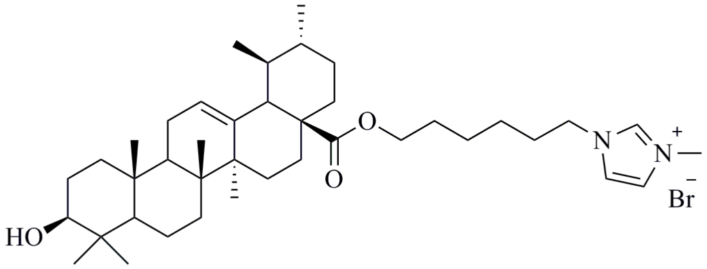

Figure S39. ^{13}C NMR Spectrum of compound **10c** (100 MHz, DMSO-d$_6$)

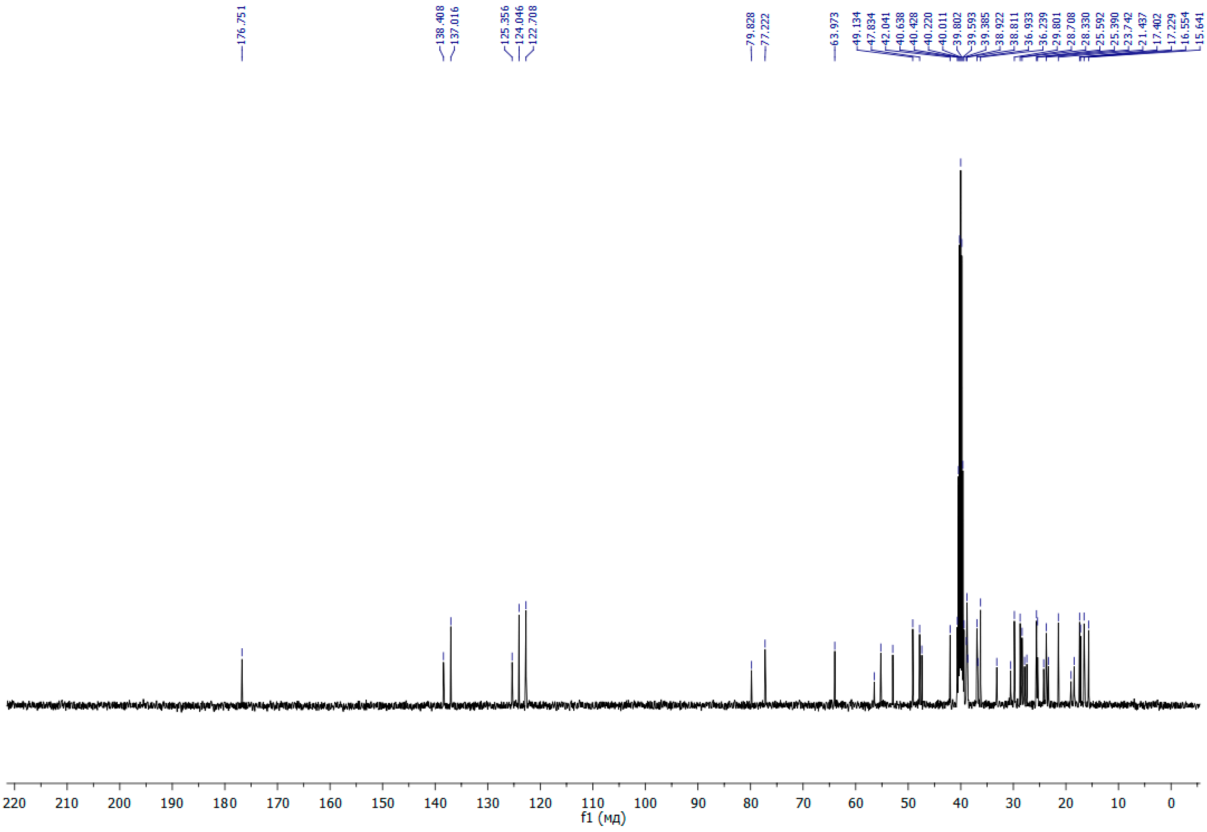

Figure S40. ^{1}H NMR Spectrum of compound **10c** (400 MHz, DMSO-d$_6$)

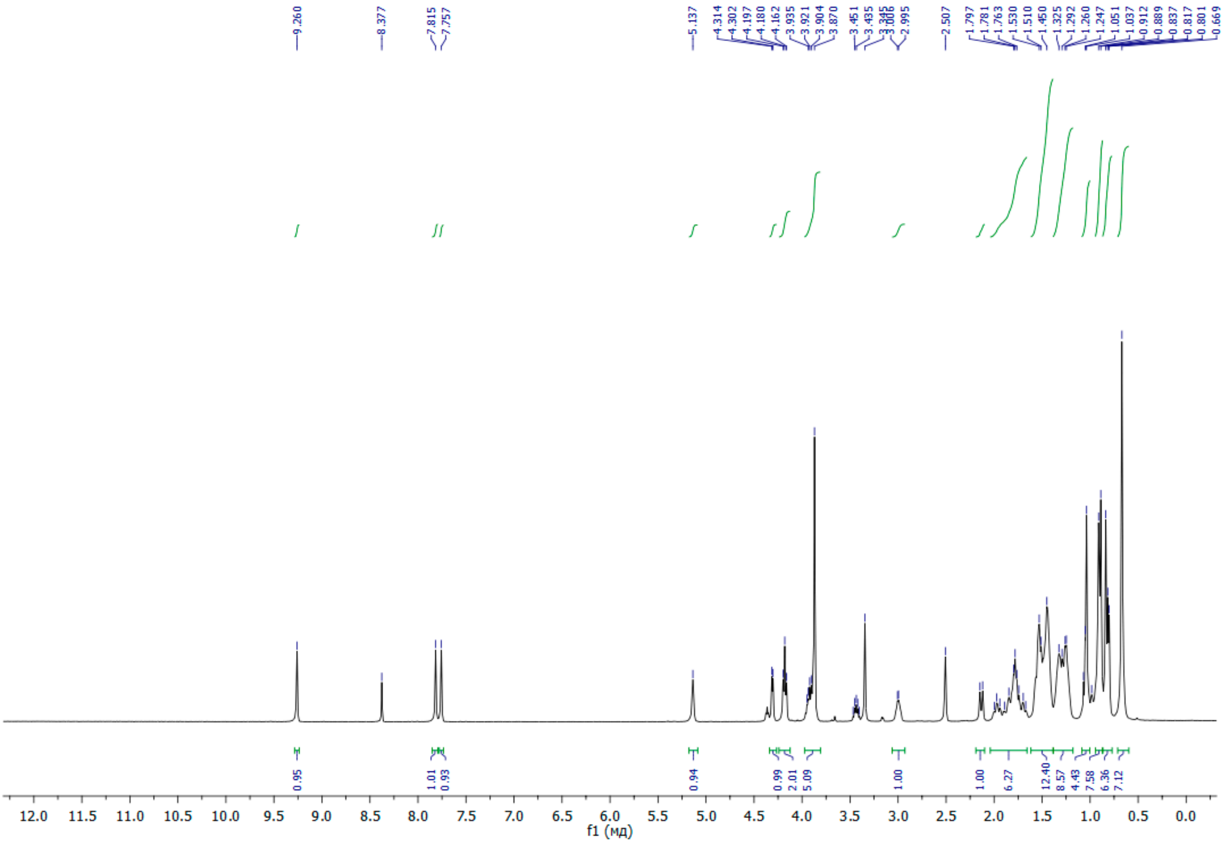

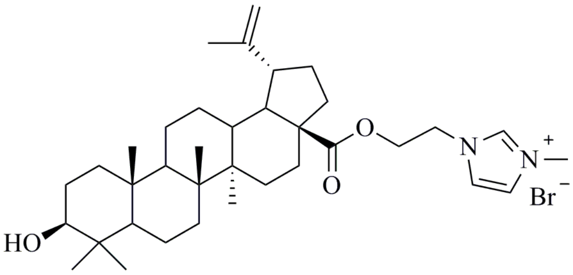

Figure S41. ^{13}C NMR Spectrum of compound **11a** (100 MHz, DMSO-d$_6$)

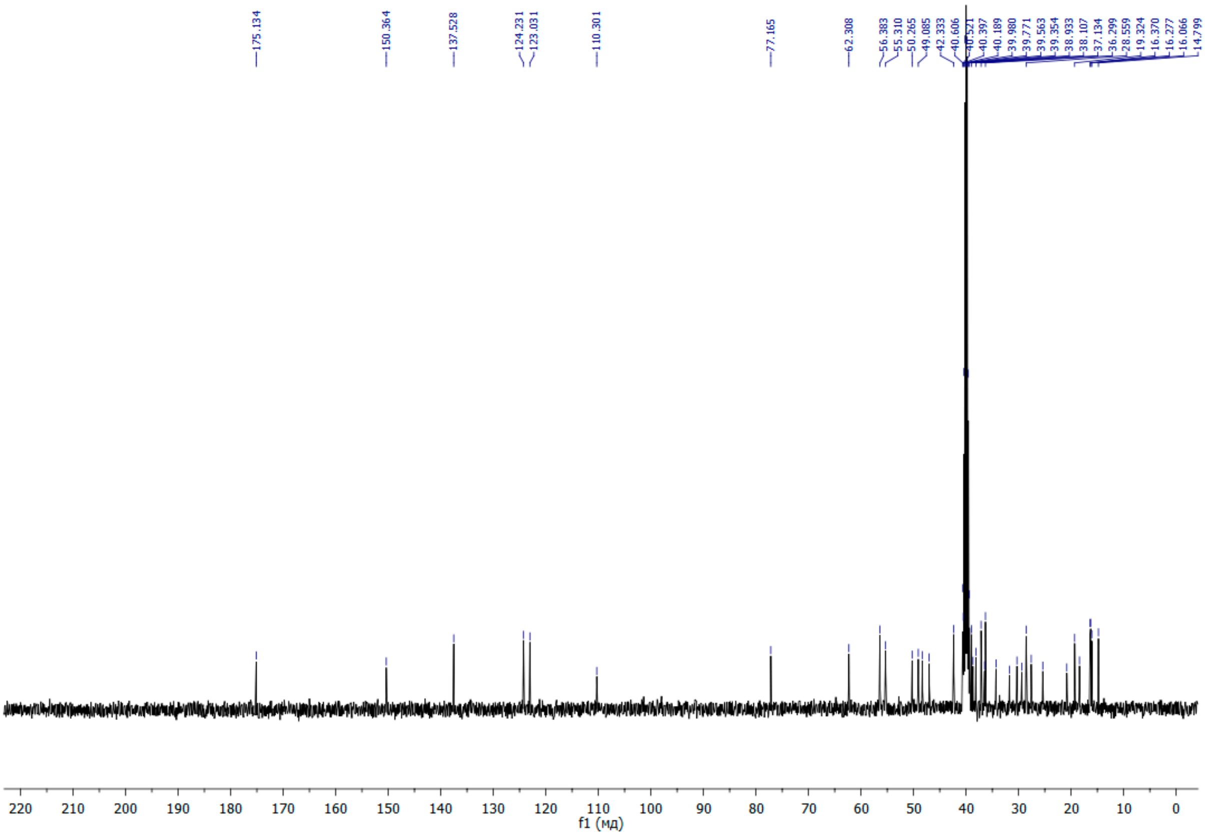

Figure S42. ^{1}H NMR Spectrum of compound **11a** (400 MHz, DMSO-d$_6$)

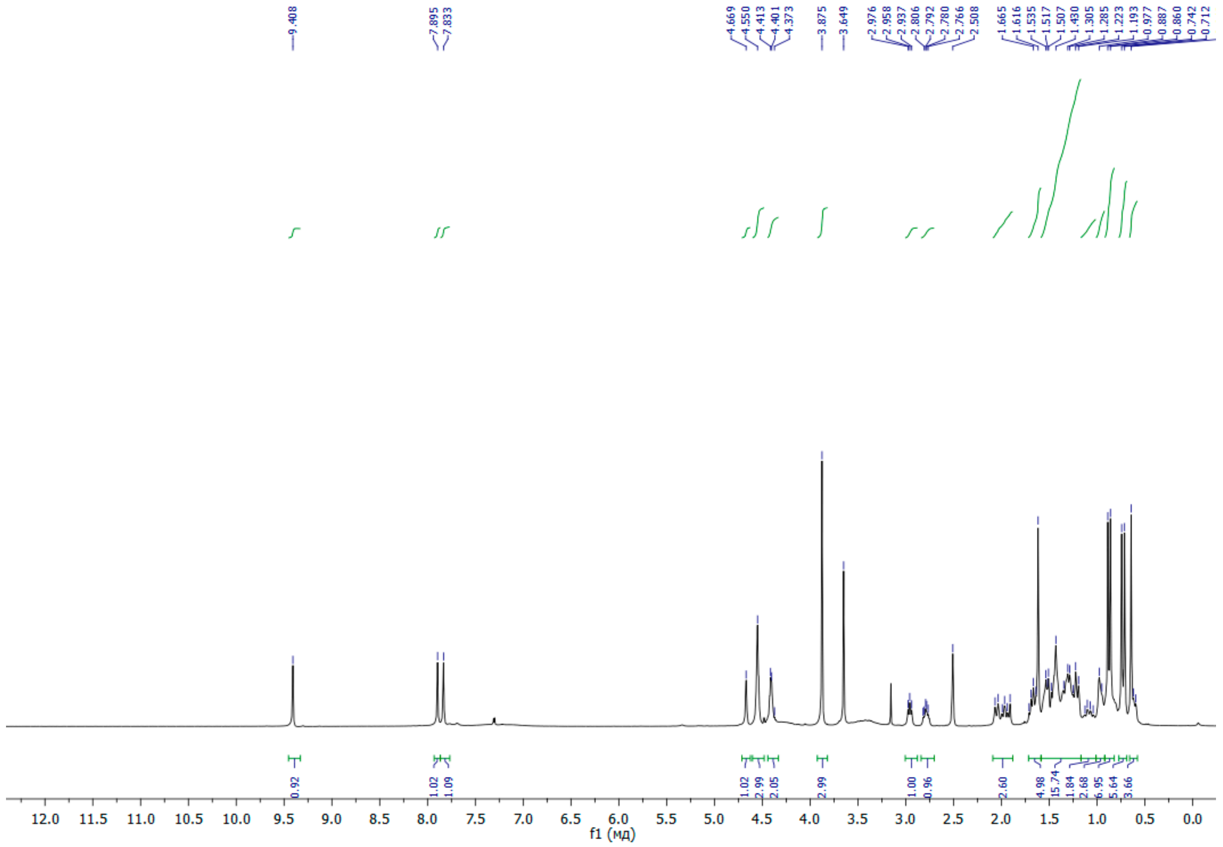

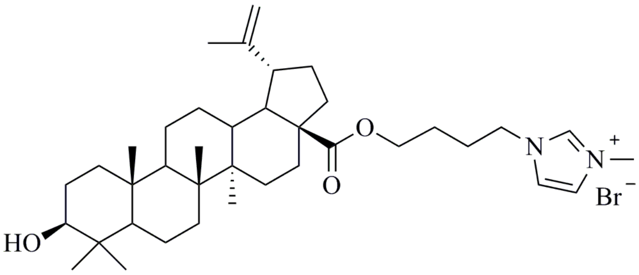

Figure S43. ^{13}C NMR Spectrum of compound **11b** (100 MHz, DMSO-d_6)

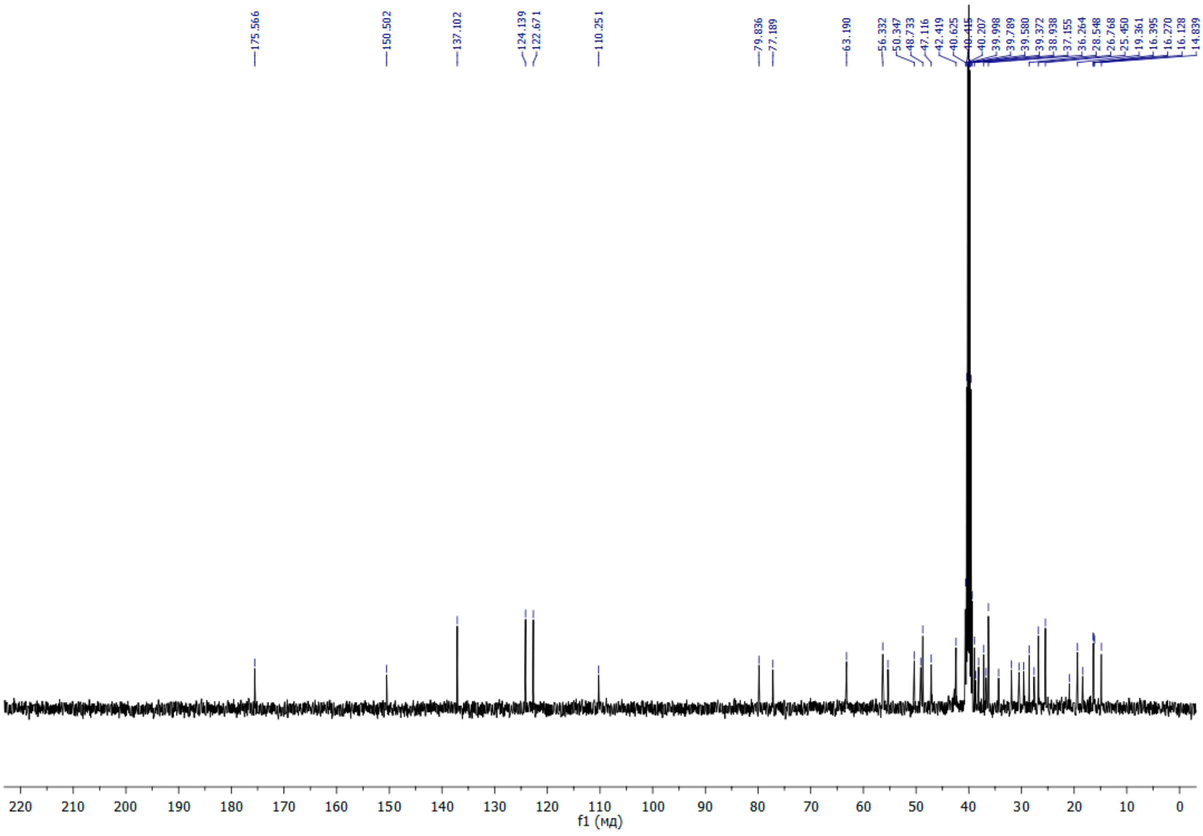

Figure S44. ^{1}H NMR Spectrum of compound **11b** (400 MHz, DMSO-d6)

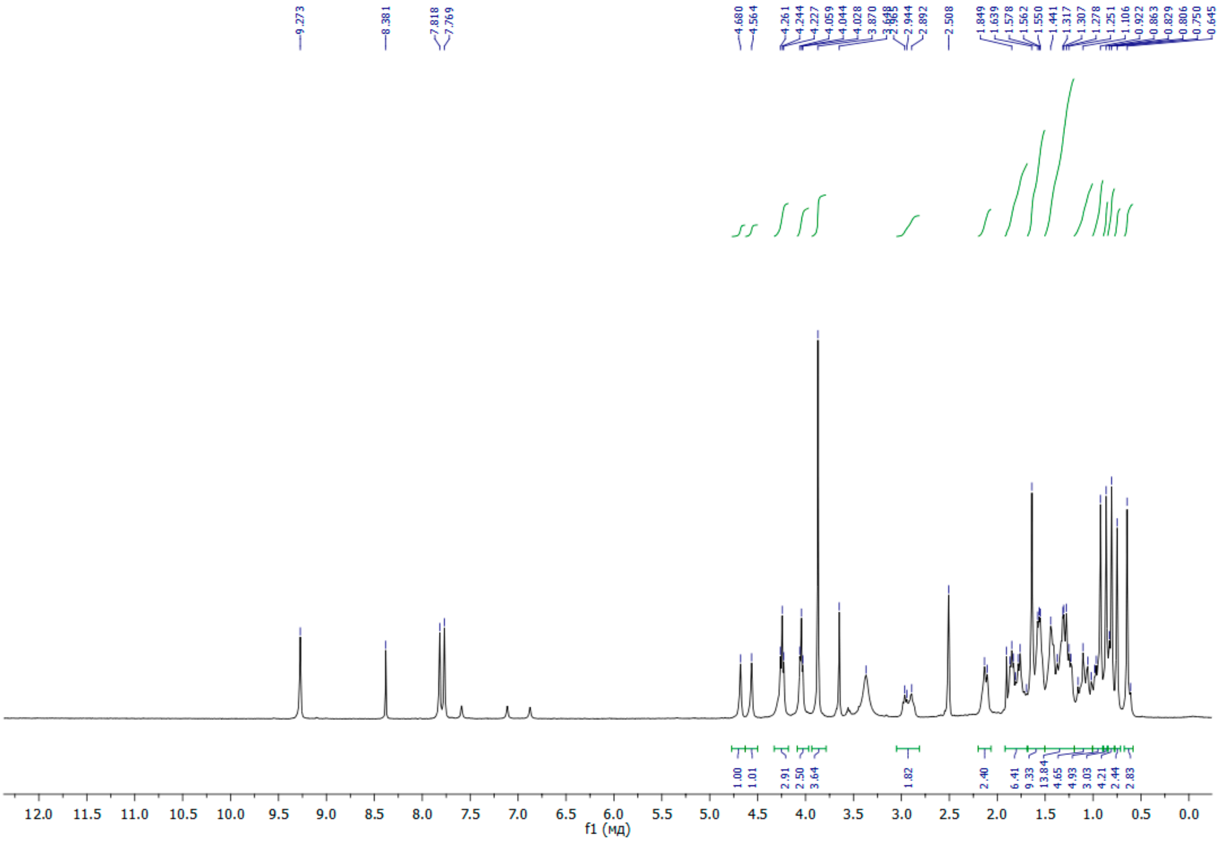

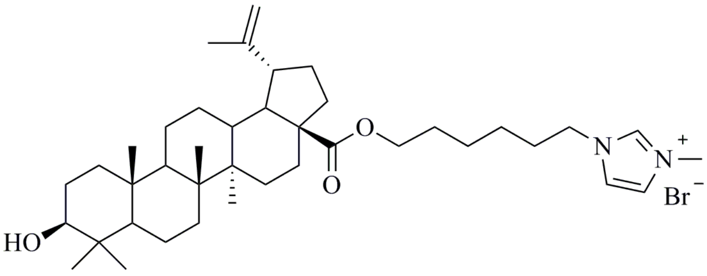

Figure S45. ^{13}C NMR Spectrum of compound **11c** (125 MHz, DMSO-d_6)

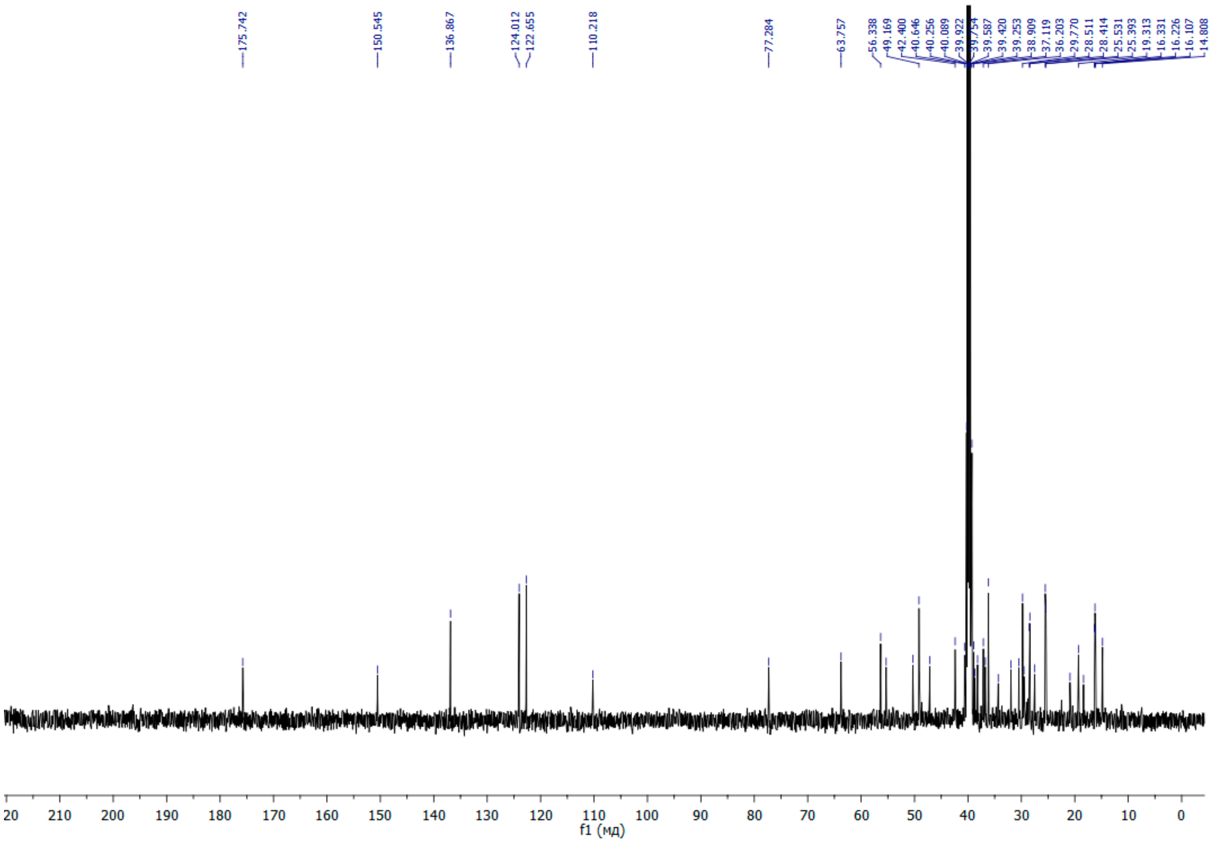

Figure S46. ^{1}H NMR Spectrum of compound **11c** (500 MHz, DMSO-d$_6$)

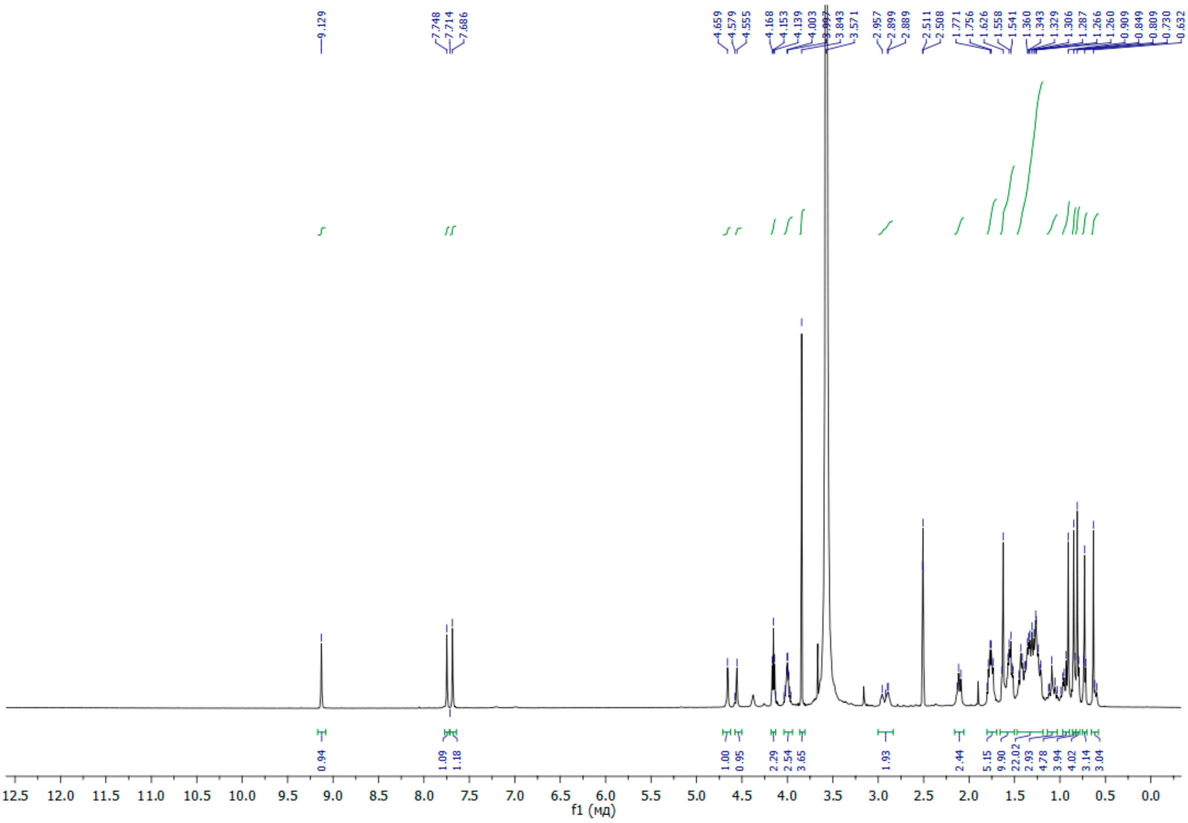

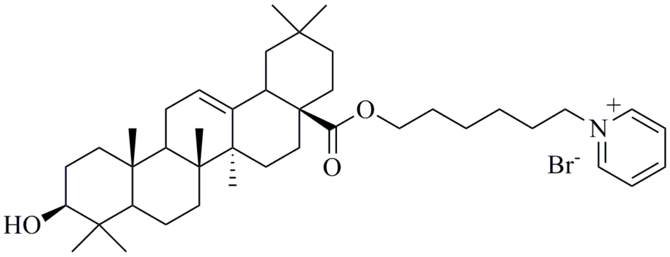

Figure S47. ^{13}C NMR Spectrum of compound **12a** (125 MHz, DMSO-d$_6$)

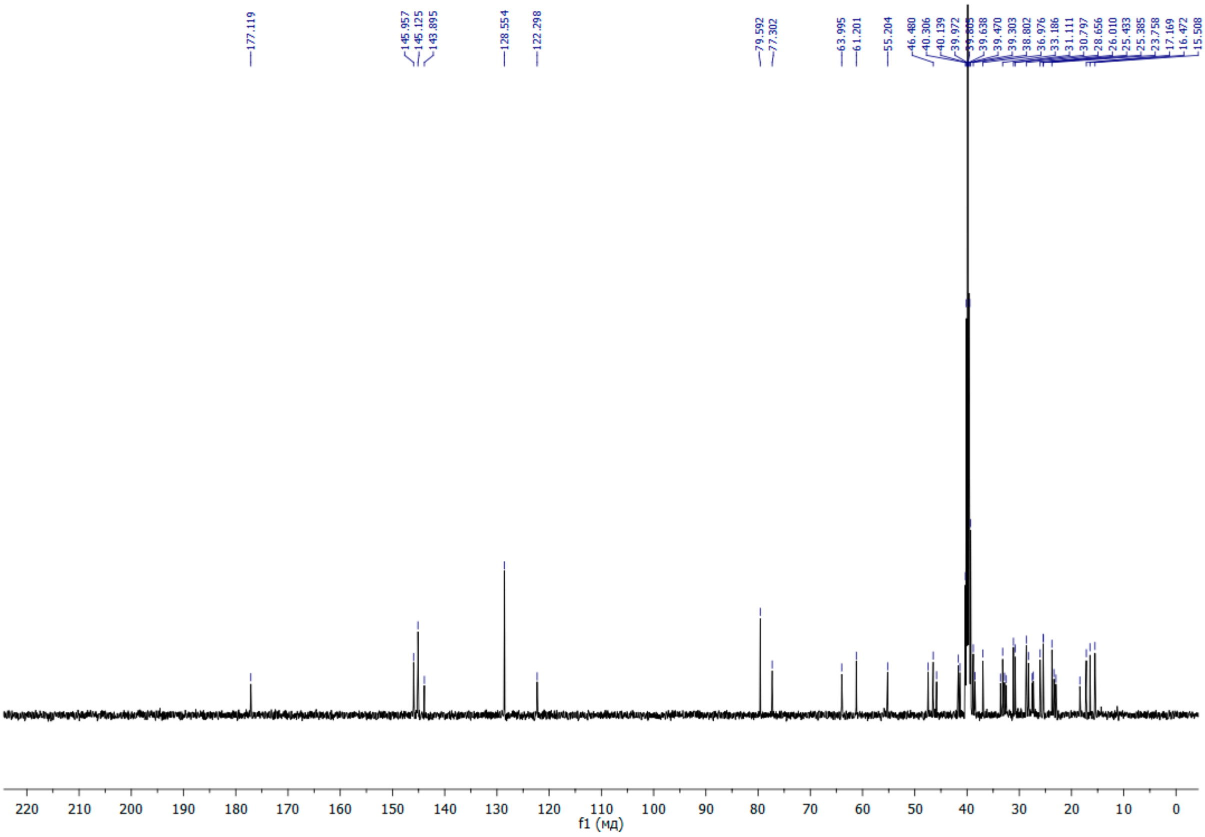

Figure S48. ^{1}H NMR Spectrum of compound **12a** (500 MHz, DMSO-d6)

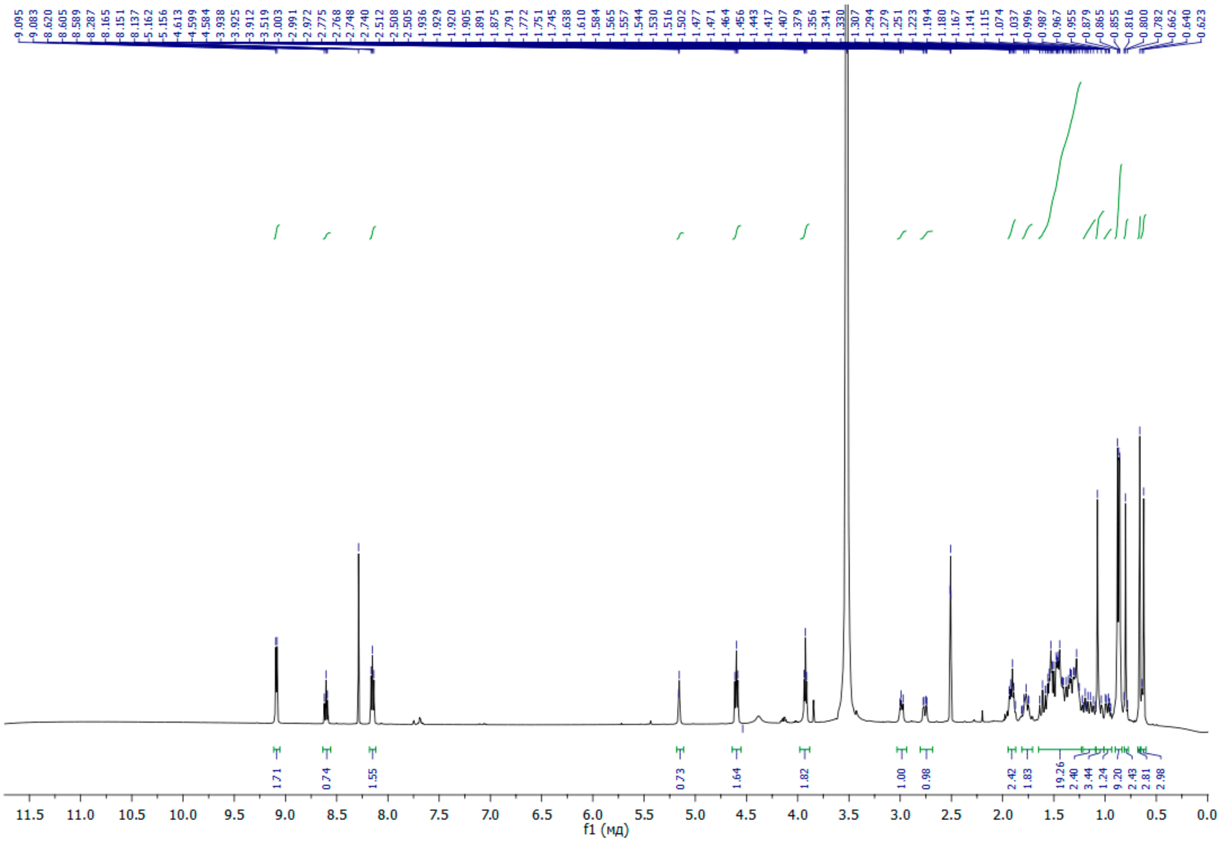

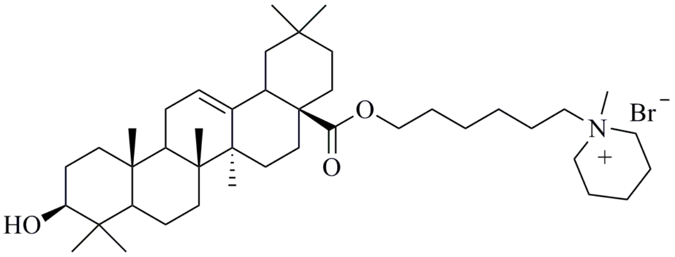

Figure S49. ^{13}C NMR Spectrum of compound **12b** (100 MHz, DMSO-d_6)

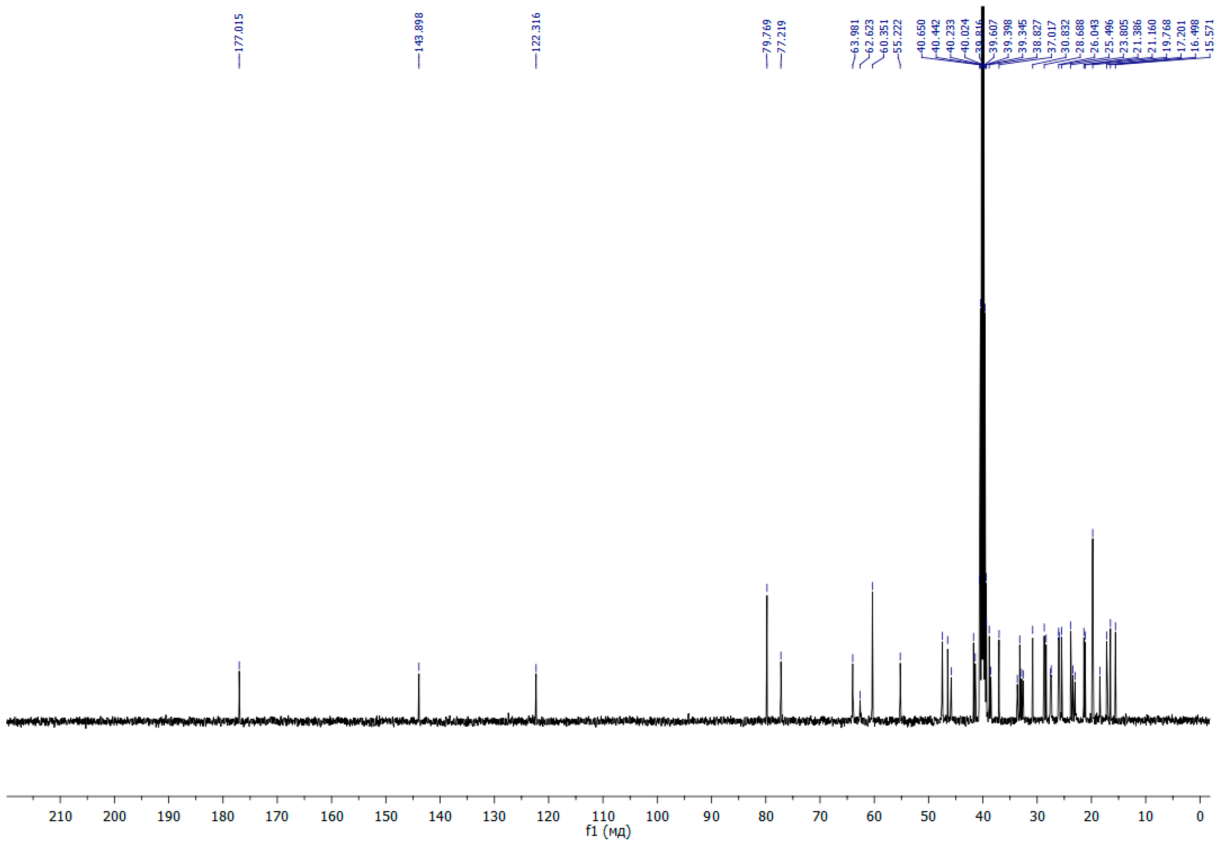

Figure S50. ^{1}H NMR Spectrum of compound **12b** (500 MHz, DMSO-d_6)

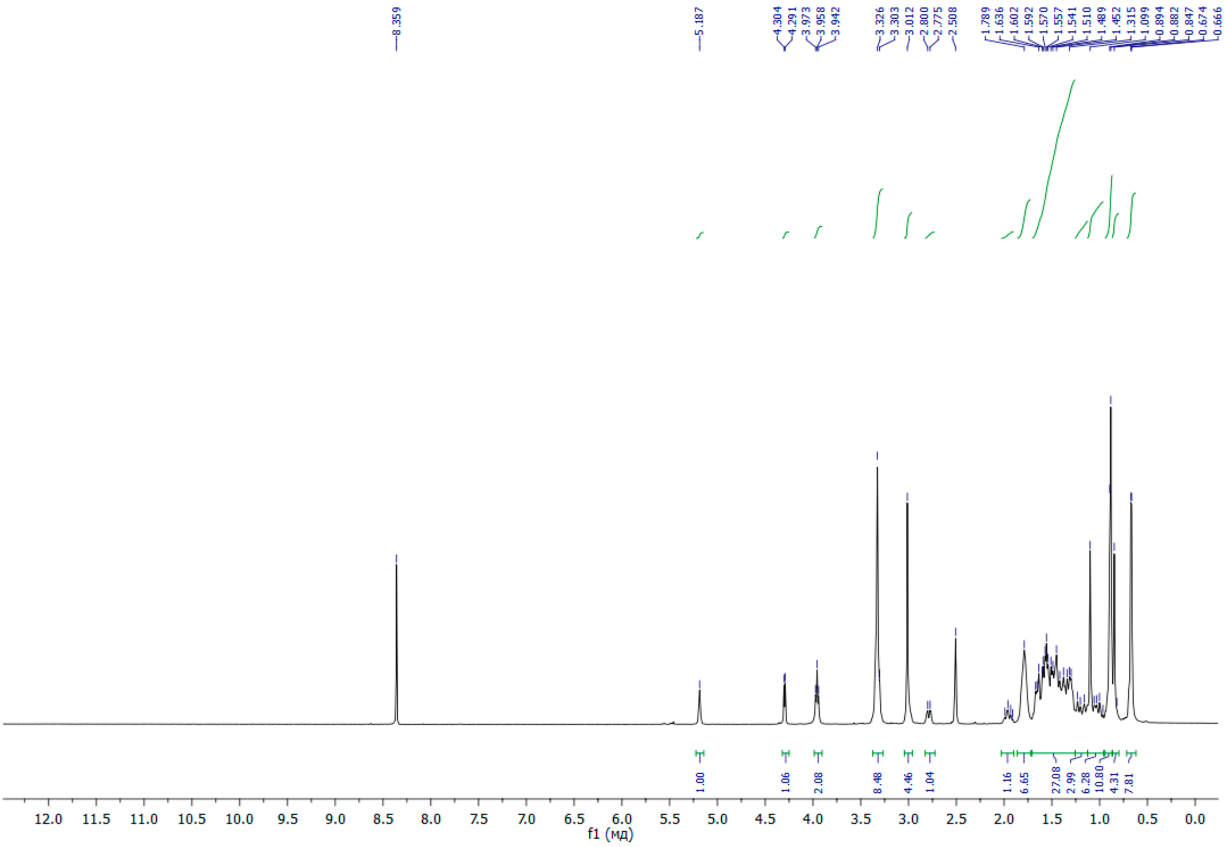

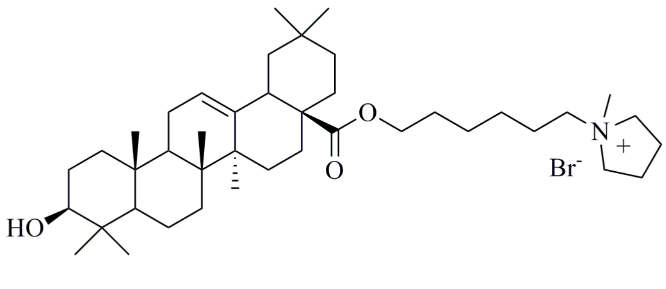

Figure S51. ^{13}C NMR Spectrum of compound **12c** (125 MHz, DMSO-d$_6$)

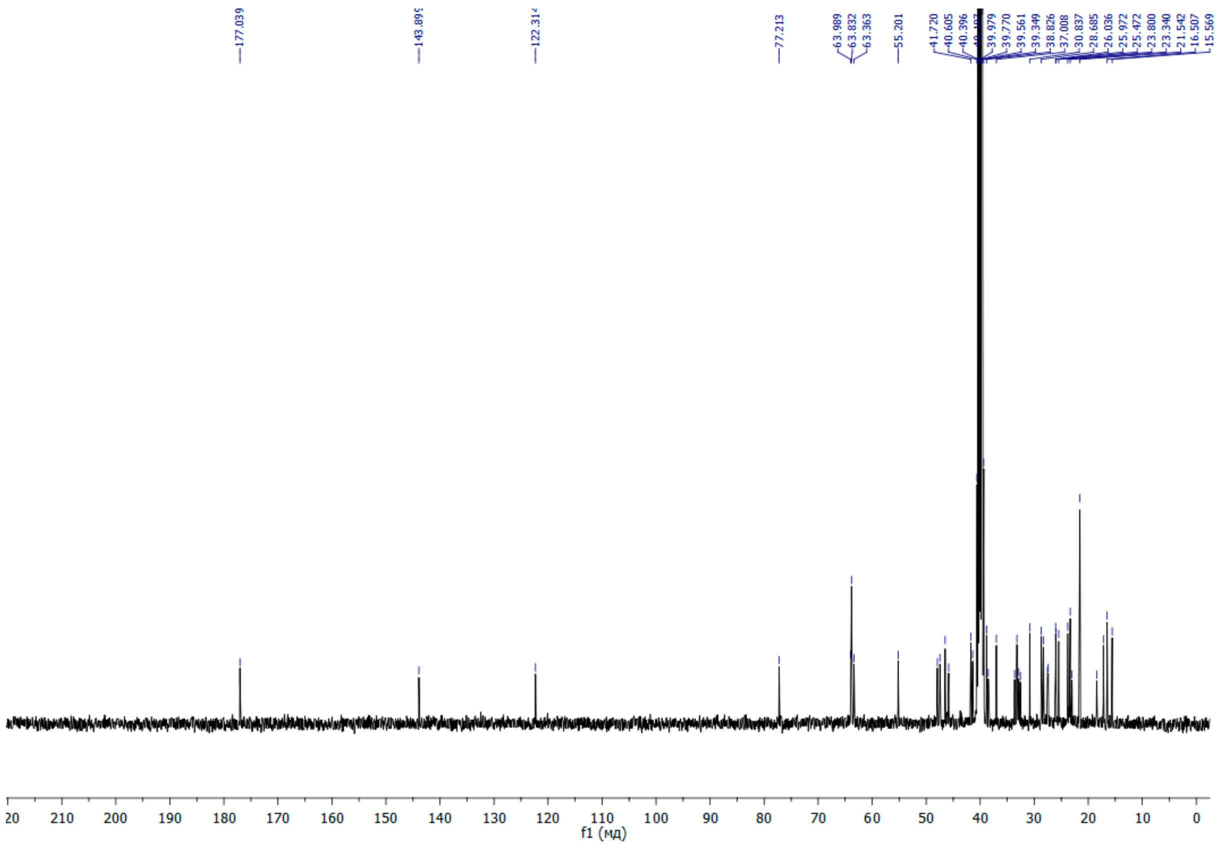

Figure S52. ¹H NMR Spectrum of compound **12c** (500 MHz, DMSO-d₆)

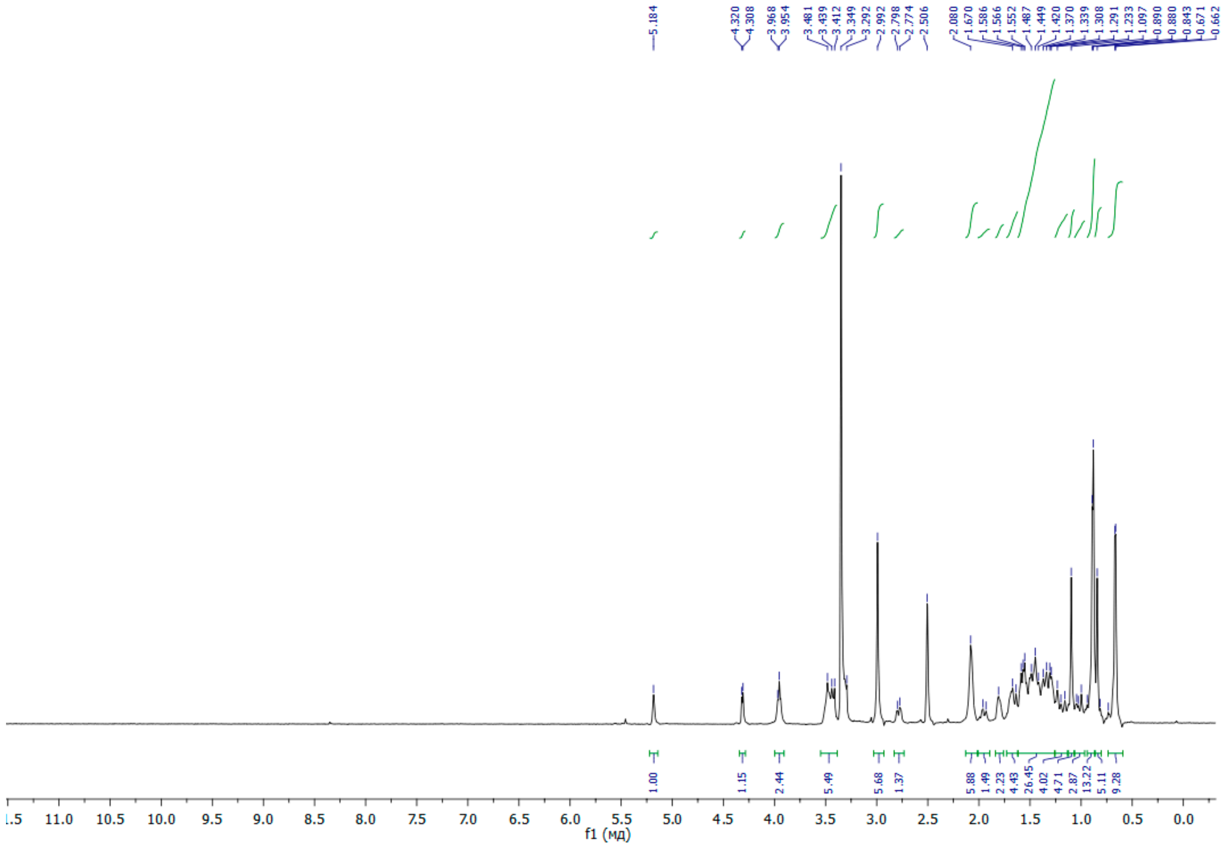

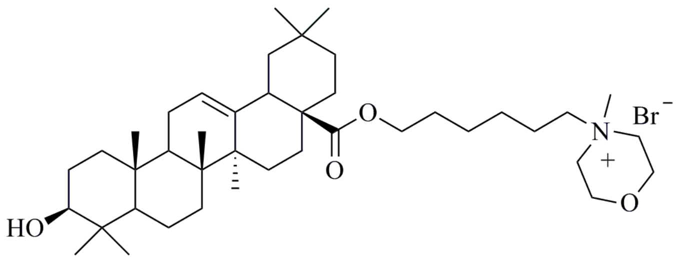

Figure S53. ^{13}C NMR Spectrum of compound **12d** (100 MHz, DMSO-d_6)

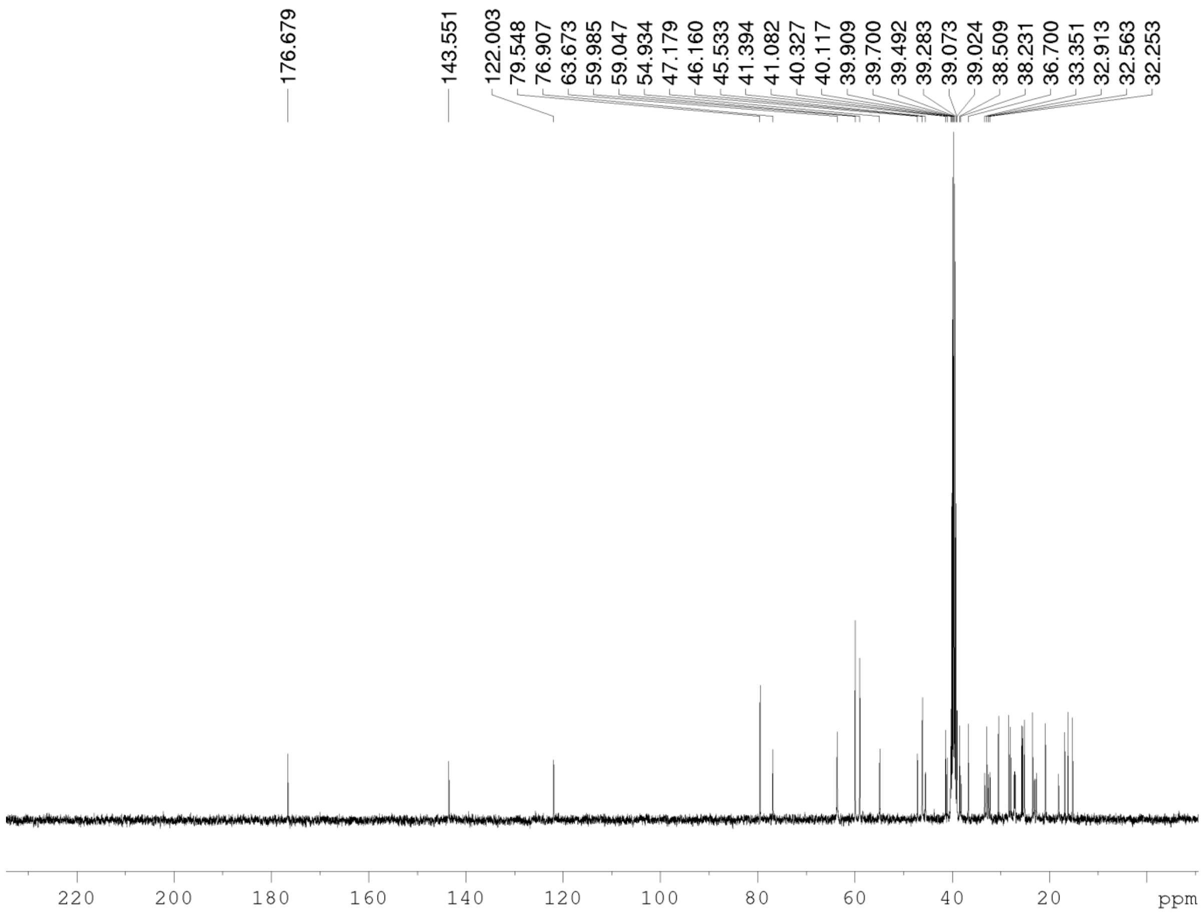

Figure S54. ^{1}H NMR Spectrum of compound **12d** (400 MHz, DMSO-d6)

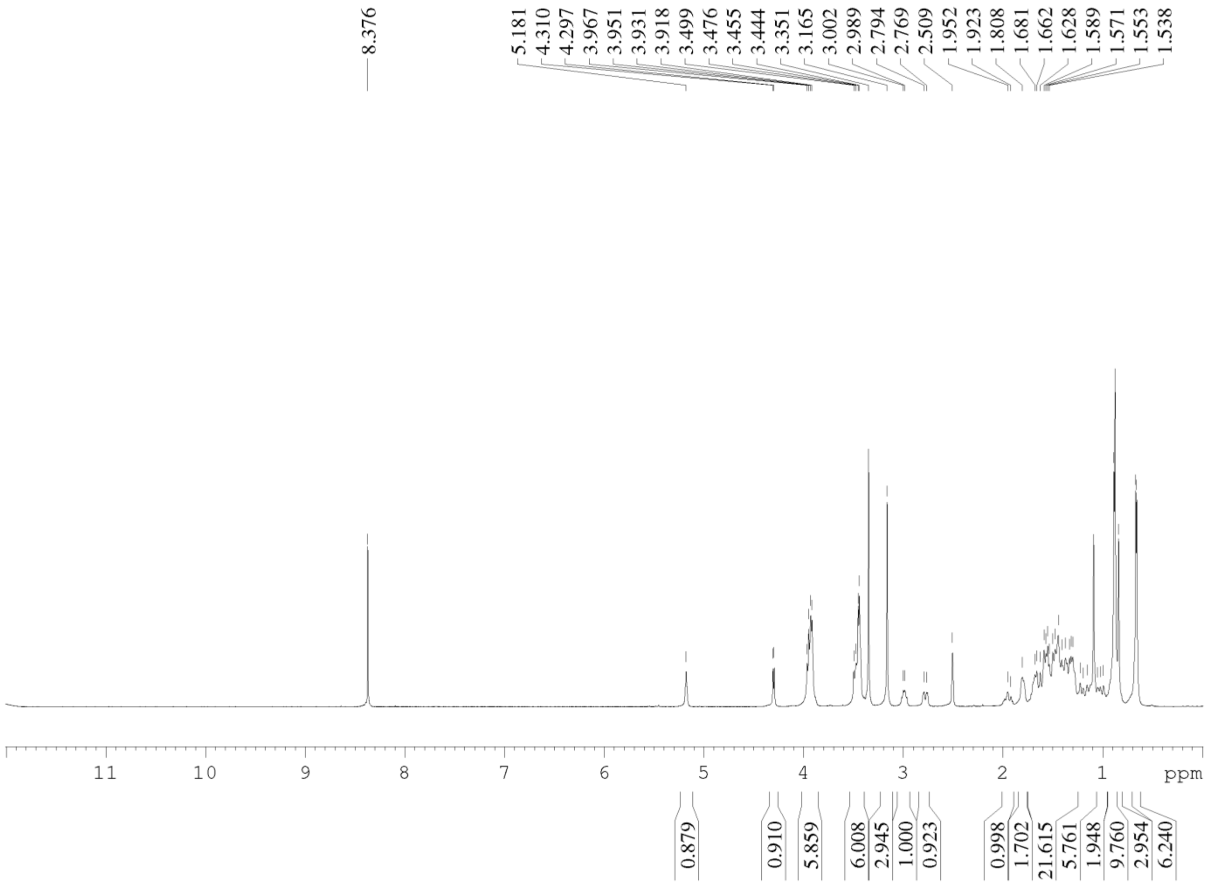

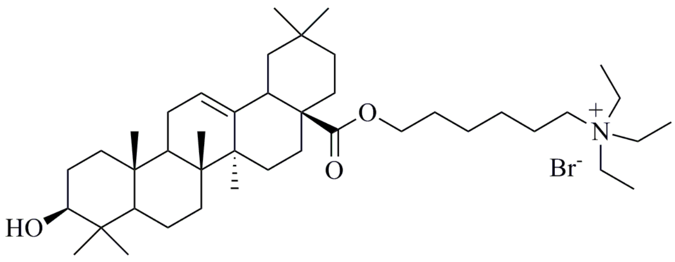

Figure S55. ^{13}C NMR Spectrum of compound **12e** (125 MHz, DMSO-d$_6$)

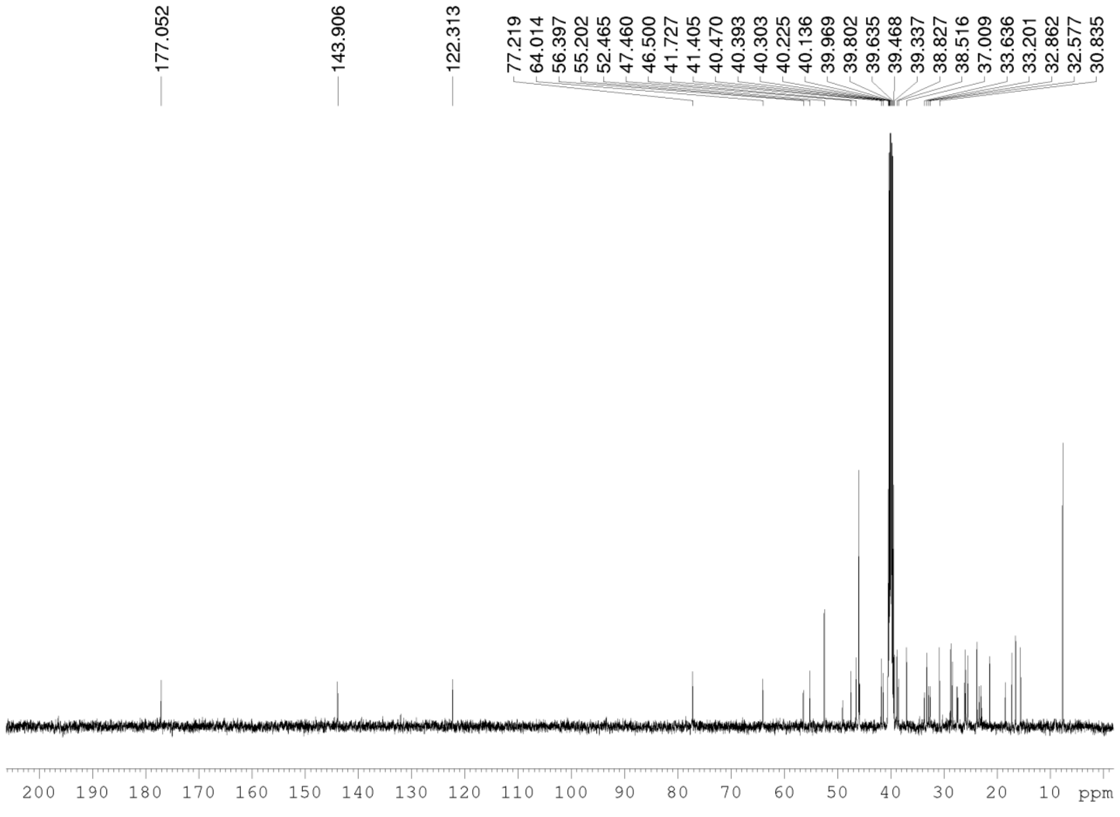

Figure S56. ^{1}H NMR Spectrum of compound **12e** (500 MHz, DMSO-d_6)

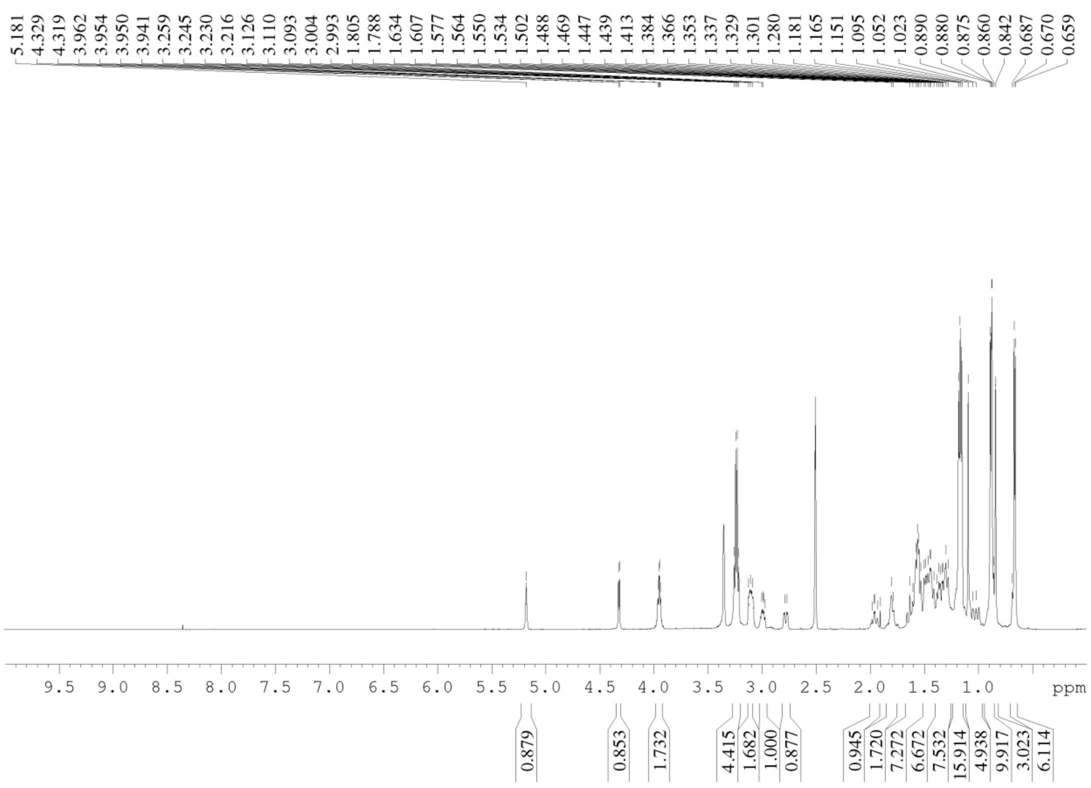

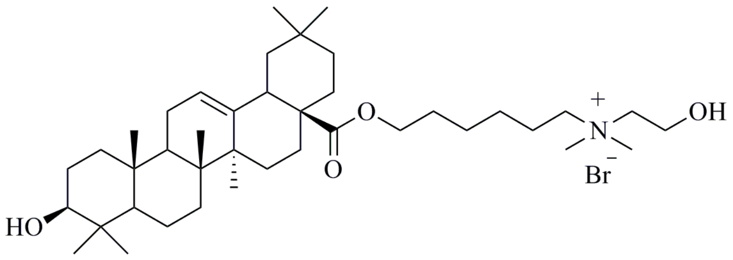

Figure S57. ^{13}C NMR Spectrum of compound **12f** (100 MHz, DMSO-d6)

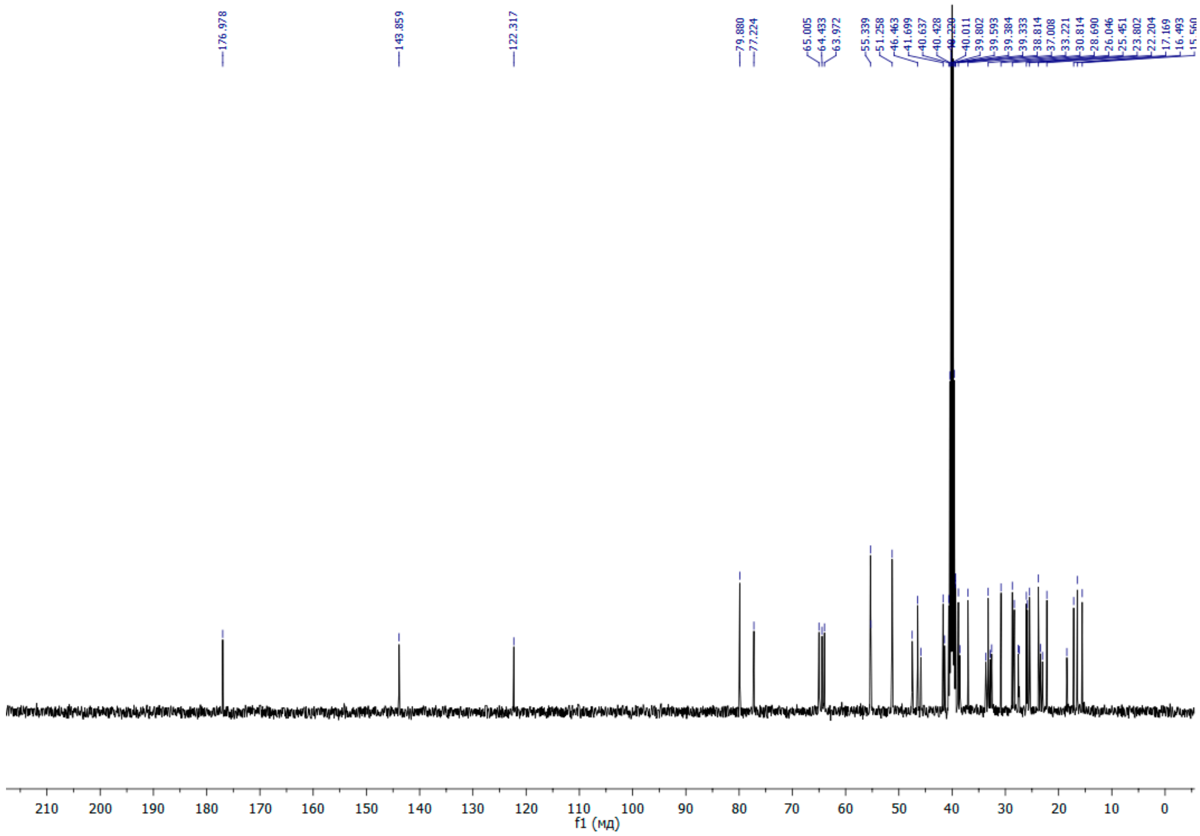

Figure S58. ^{1}H NMR Spectrum of compound **12f** (400 MHz, DMSO-d$_6$)

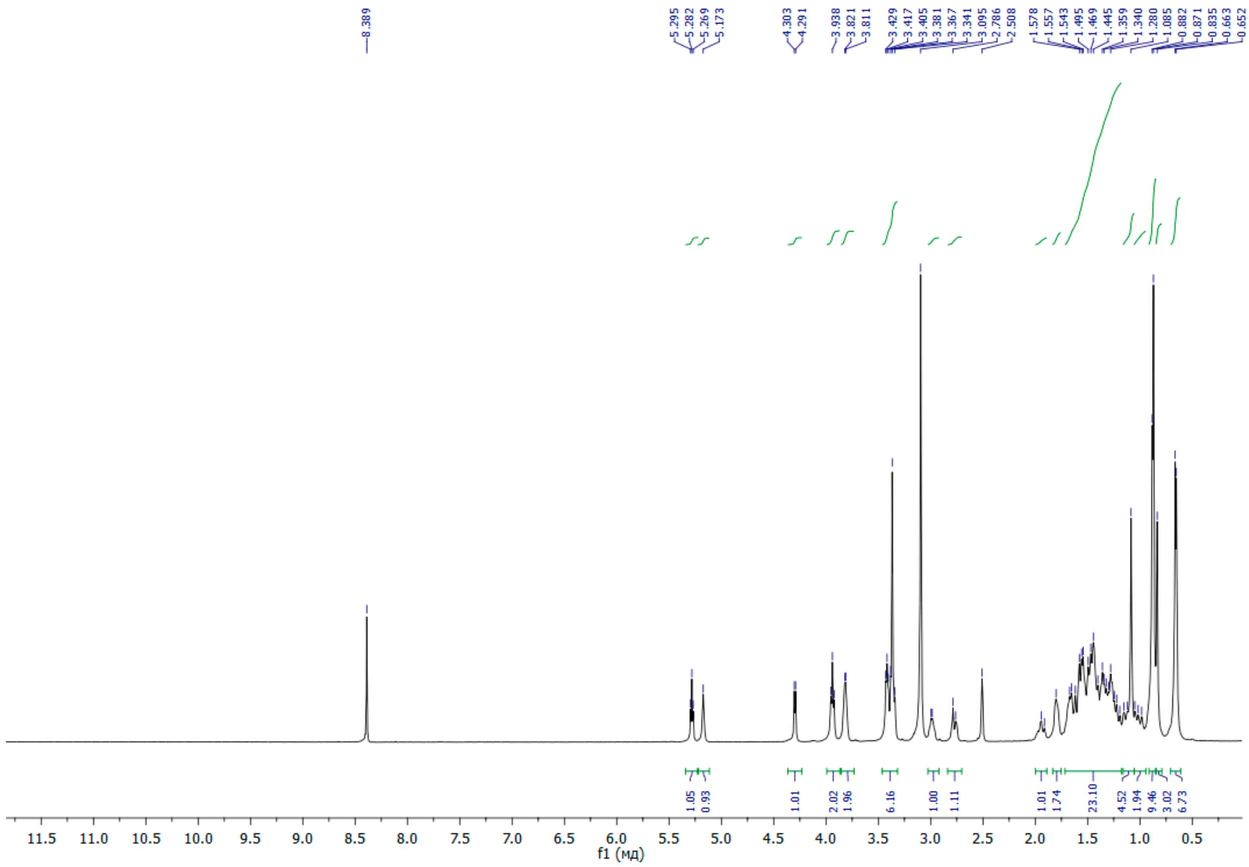

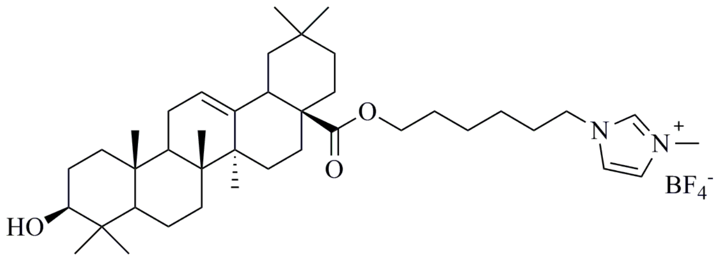

Figure S59. ^{13}C NMR Spectrum of compound **13a** (125 MHz, DMSO-d$_6$)

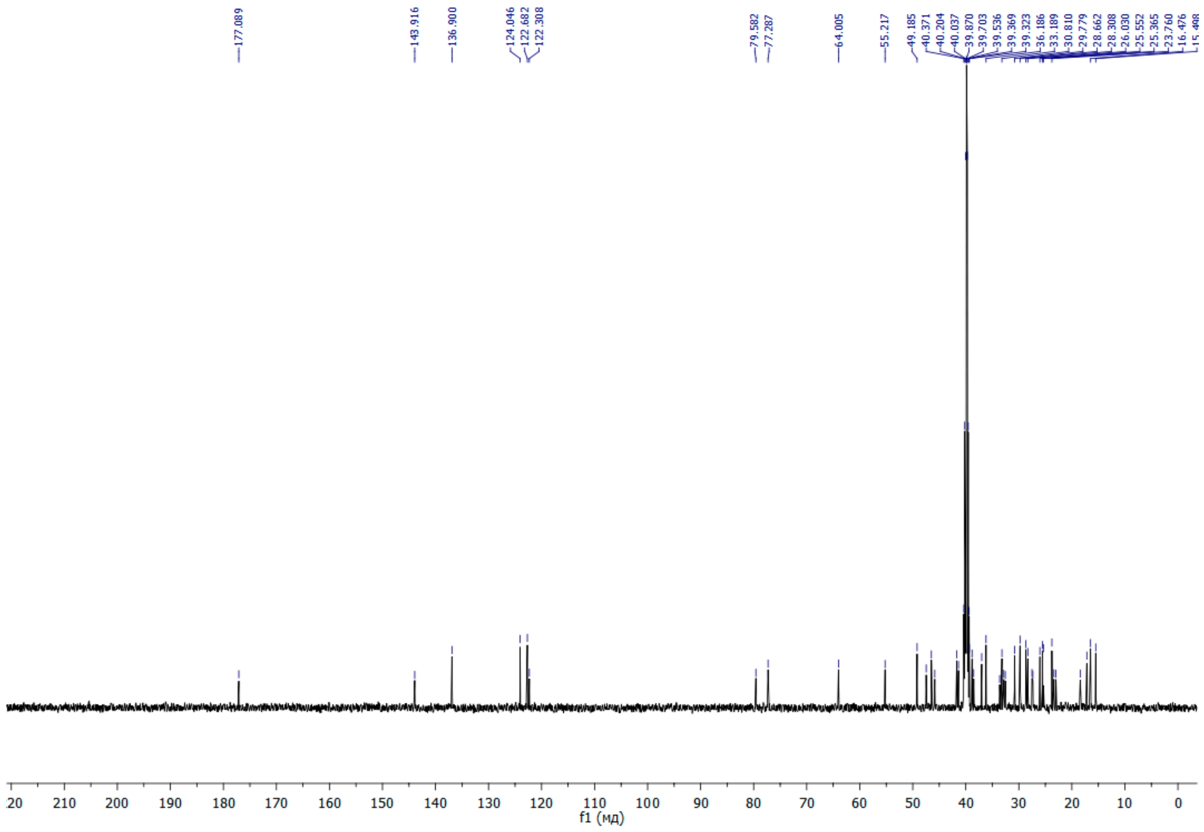

Figure S60. ^{1}H NMR Spectrum of compound **13a** (500 MHz, DMSO-d6)

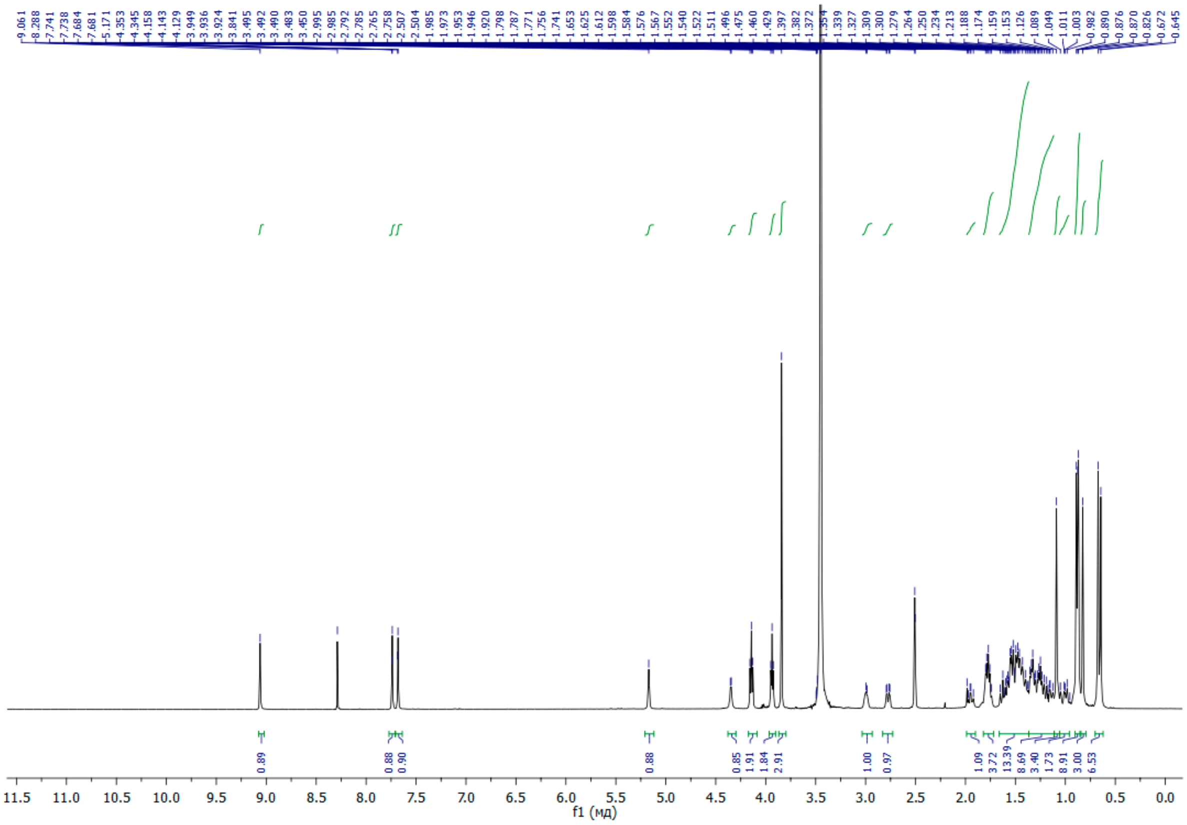

Figure S61. ^{19}F NMR Spectrum of compound **13a** (376 MHz, DMSO-d6)

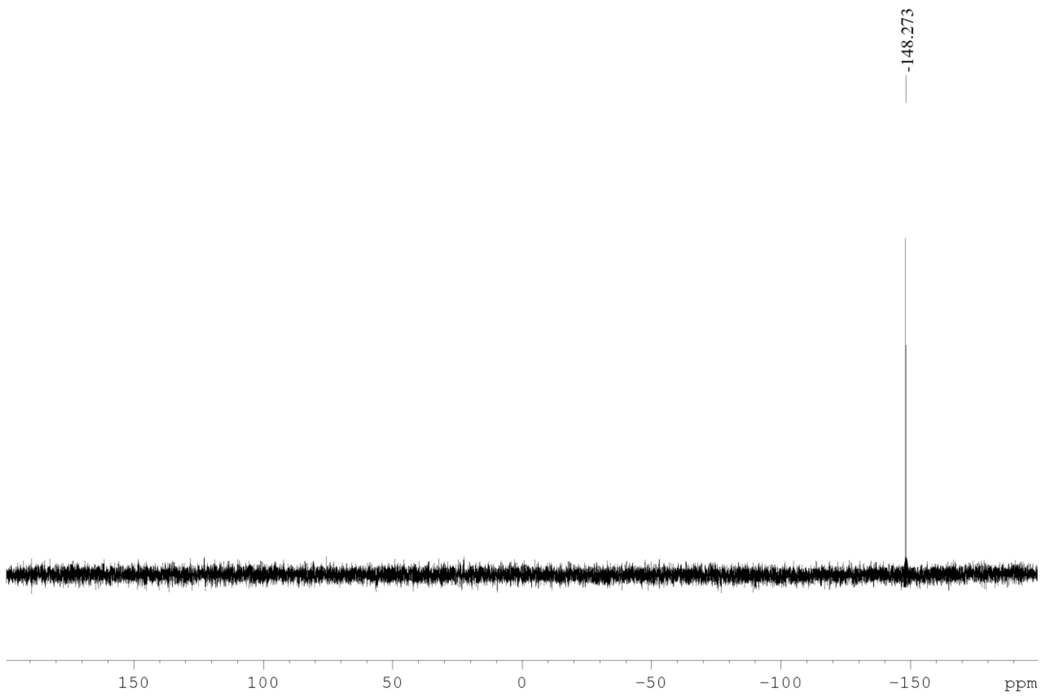

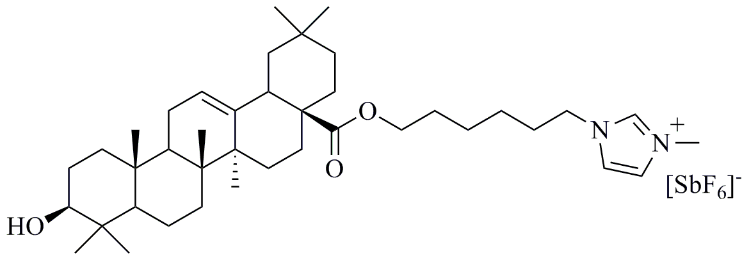

Figure S62. ^{13}C NMR Spectrum of compound **13b** (100 MHz, DMSO-d$_6$)

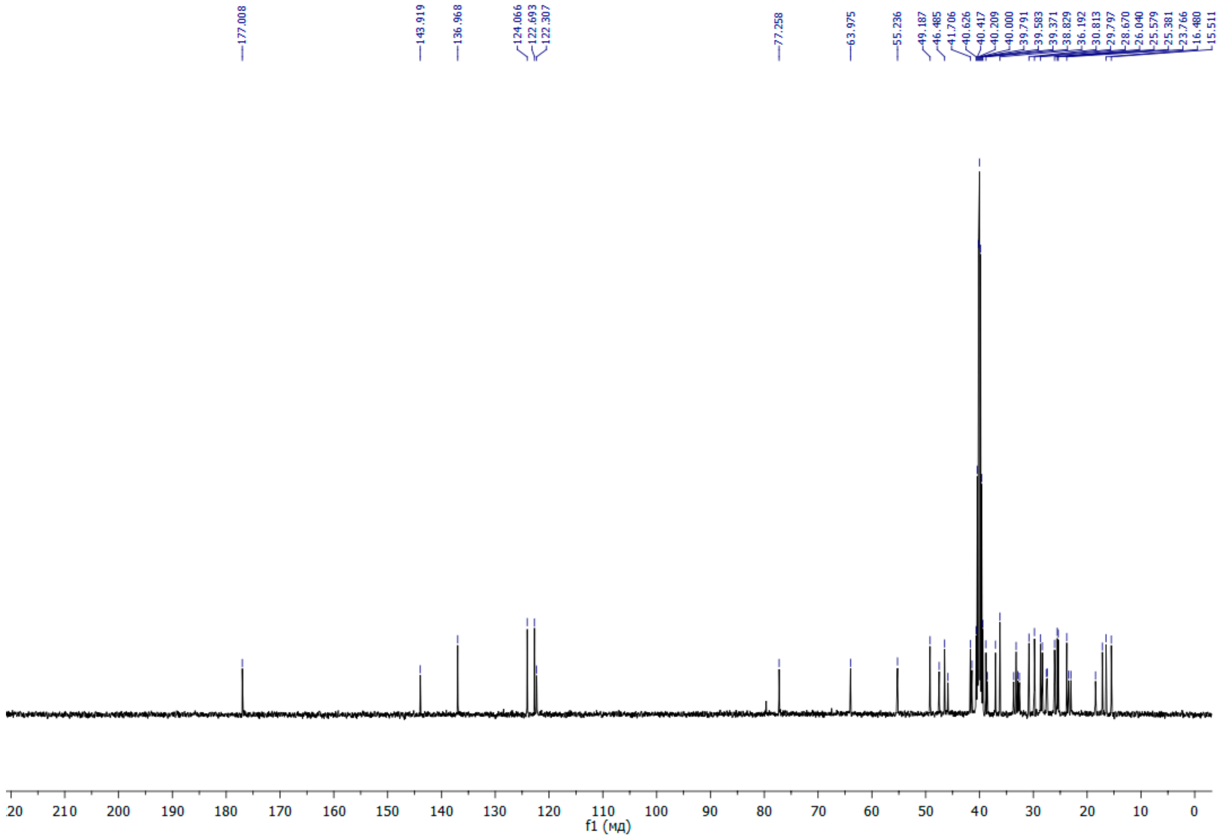

Figure S63. ^{1}H NMR Spectrum of compound **13b** (400 MHz, DMSO-d$_6$)

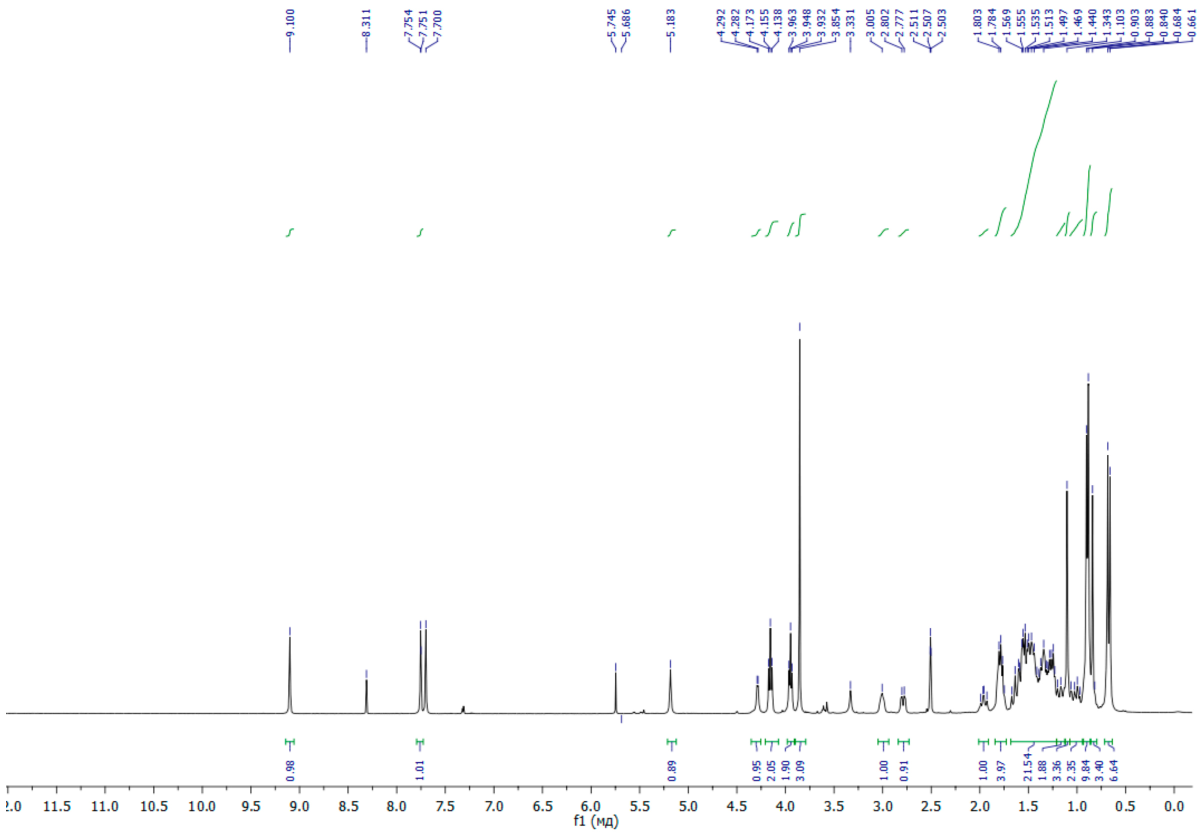

Figure S64. ^{19}F NMR Spectrum of compound **13b** (376 MHz, DMSO-d_6)

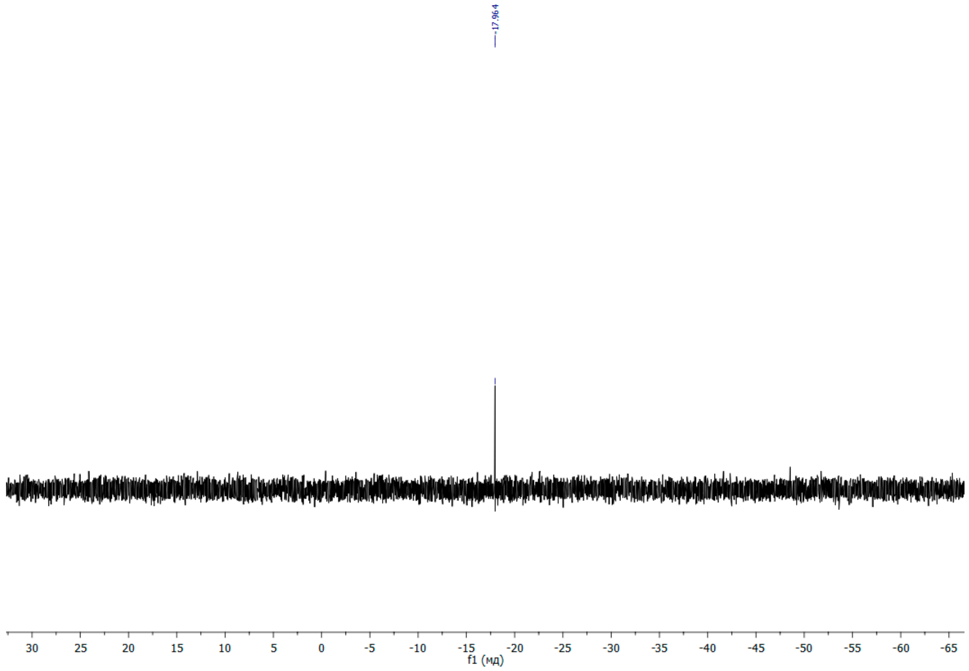

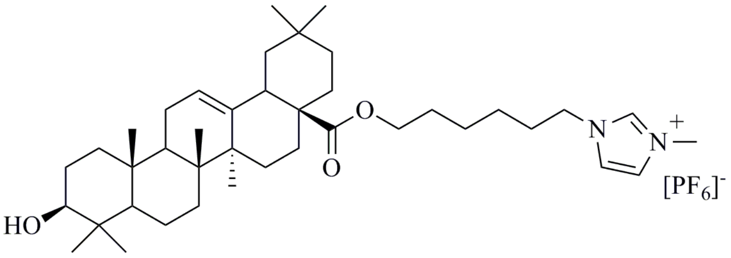

Figure S65. ^{13}C NMR Spectrum of compound **13c** (100 MHz, DMSO-d6)

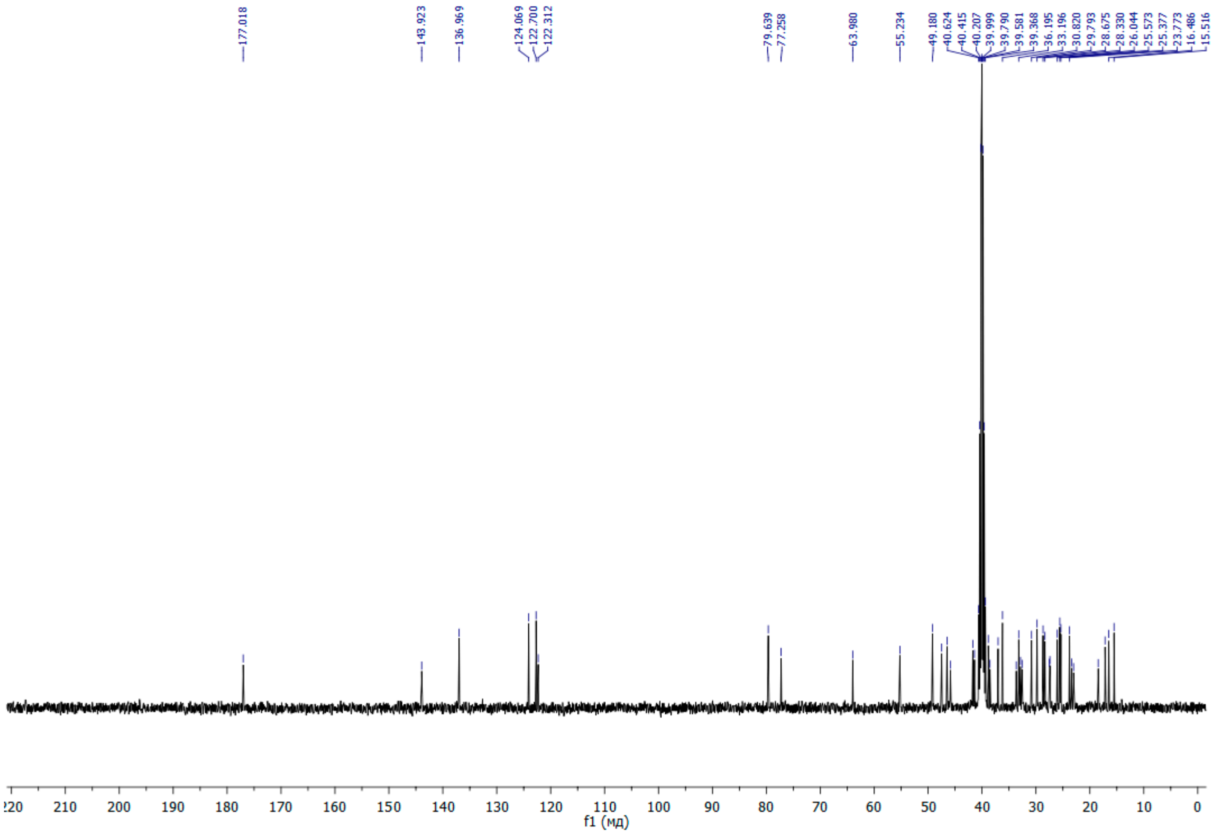

Figure S66. ^{1}H NMR Spectrum of compound **13c** (400 MHz, DMSO-d$_6$)

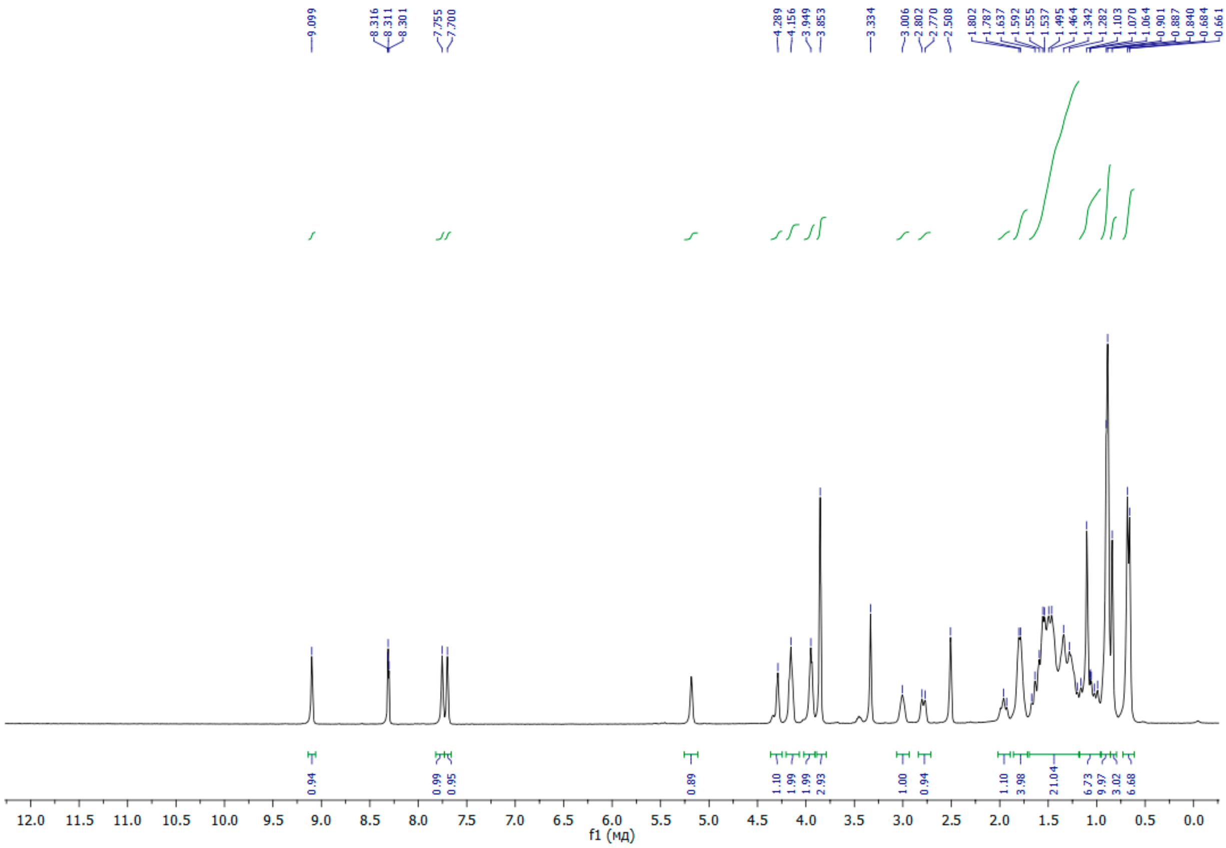

Figure S67. ^{19}F NMR Spectrum of compound **13c** (376 MHz, DMSO-d$_6$)

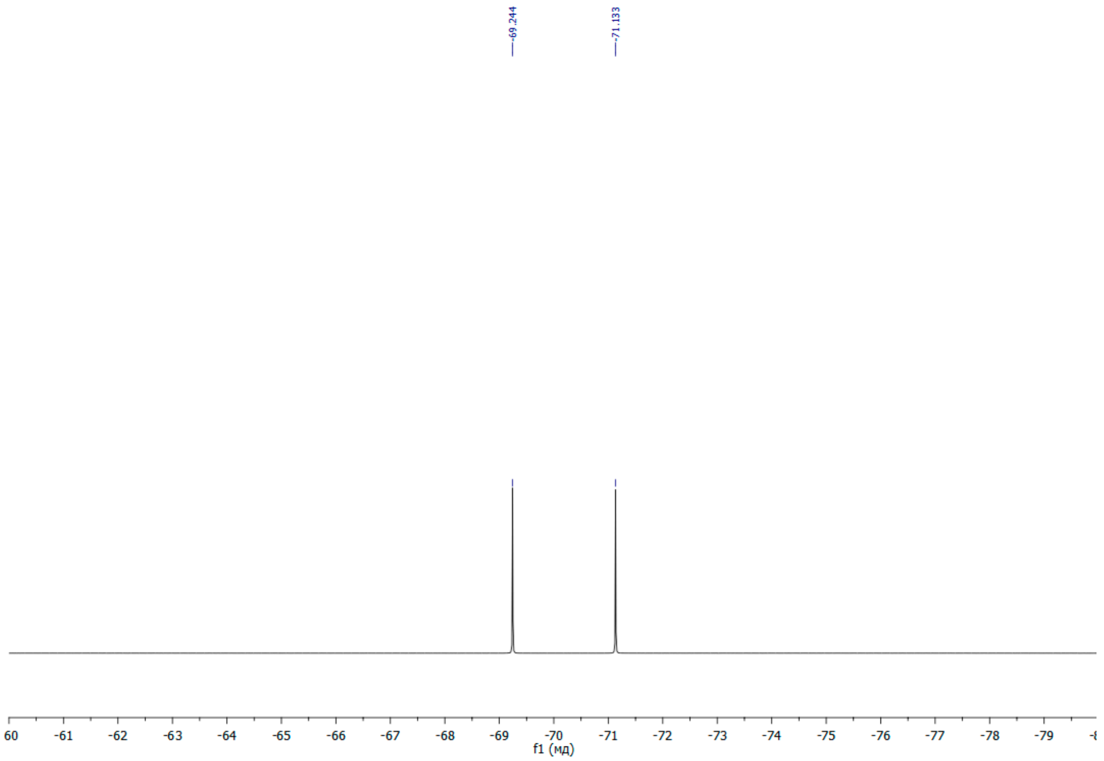

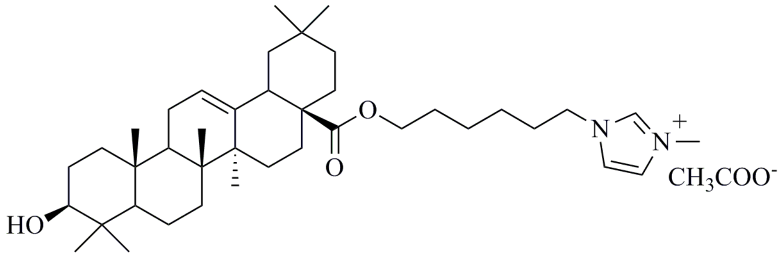

Figure S68. ^{13}C NMR Spectrum of compound **13d** (100 MHz, DMSO-d6)

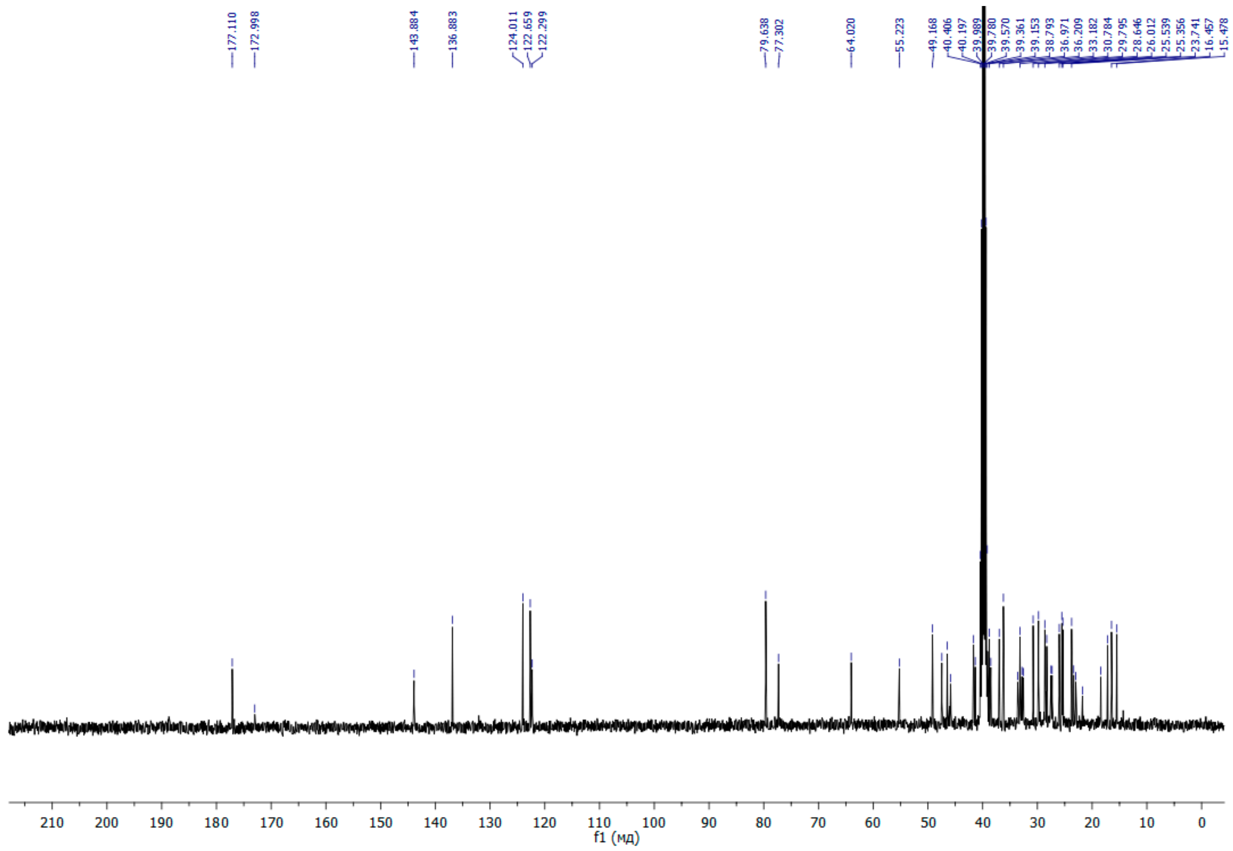

Figure S69. ^{1}H NMR Spectrum of compound **13d** (400 MHz, DMSO-d6)

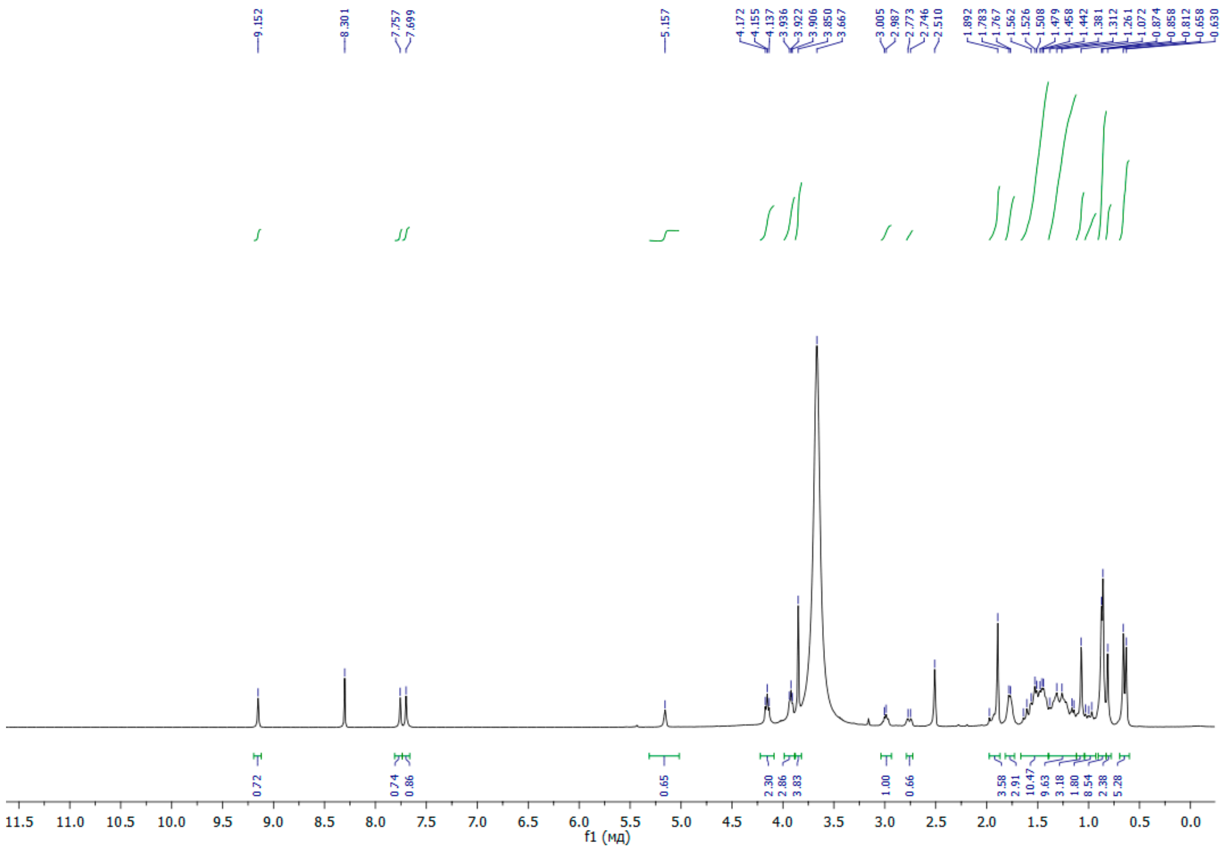

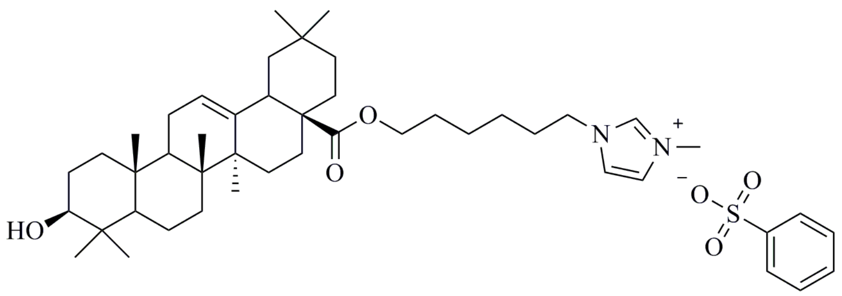

Figure S70. ^{13}C NMR Spectrum of compound **13e** (100 MHz, DMSO-d$_6$)

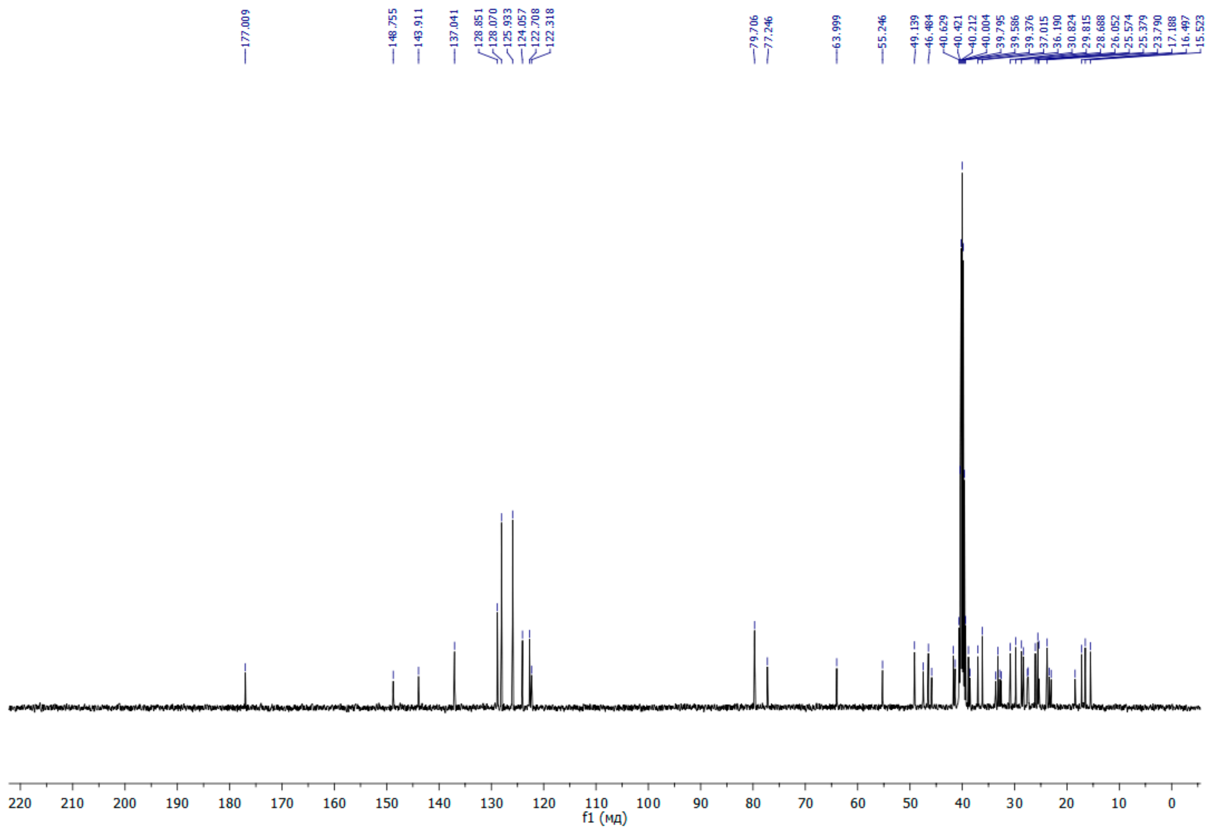

Figure S71. ^{1}H NMR Spectrum of compound **13e** (400 MHz, DMSO-d_6)

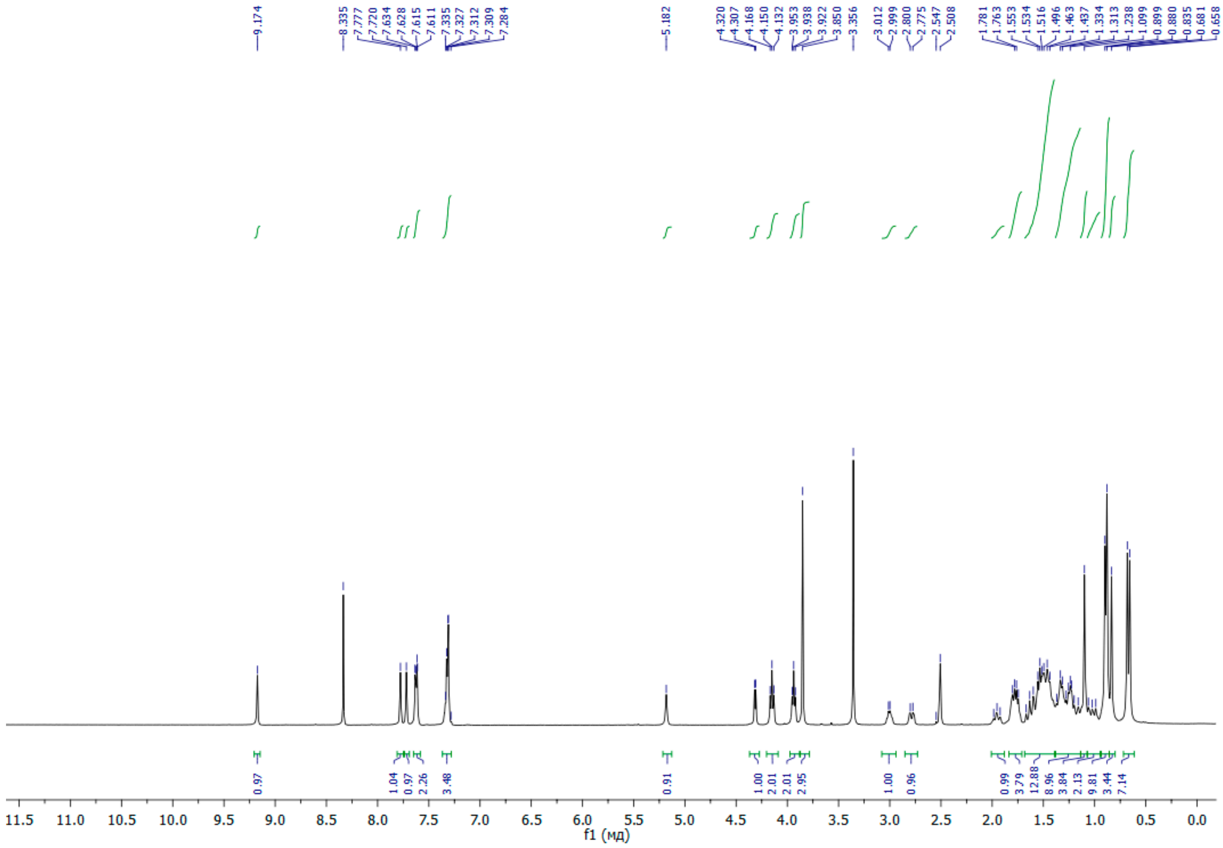

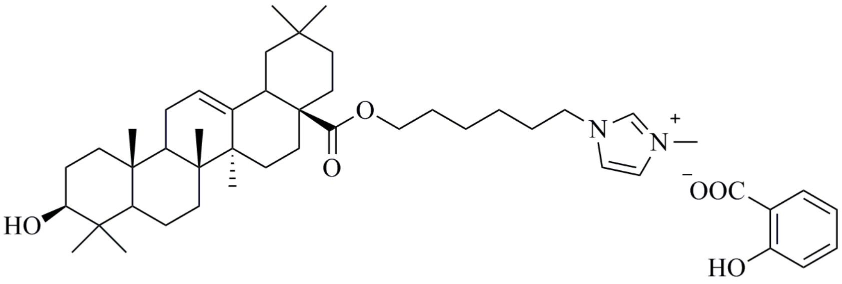

Figure S72. ^{13}C NMR Spectrum of compound **13f** (100 MHz, DMSO-d_6)

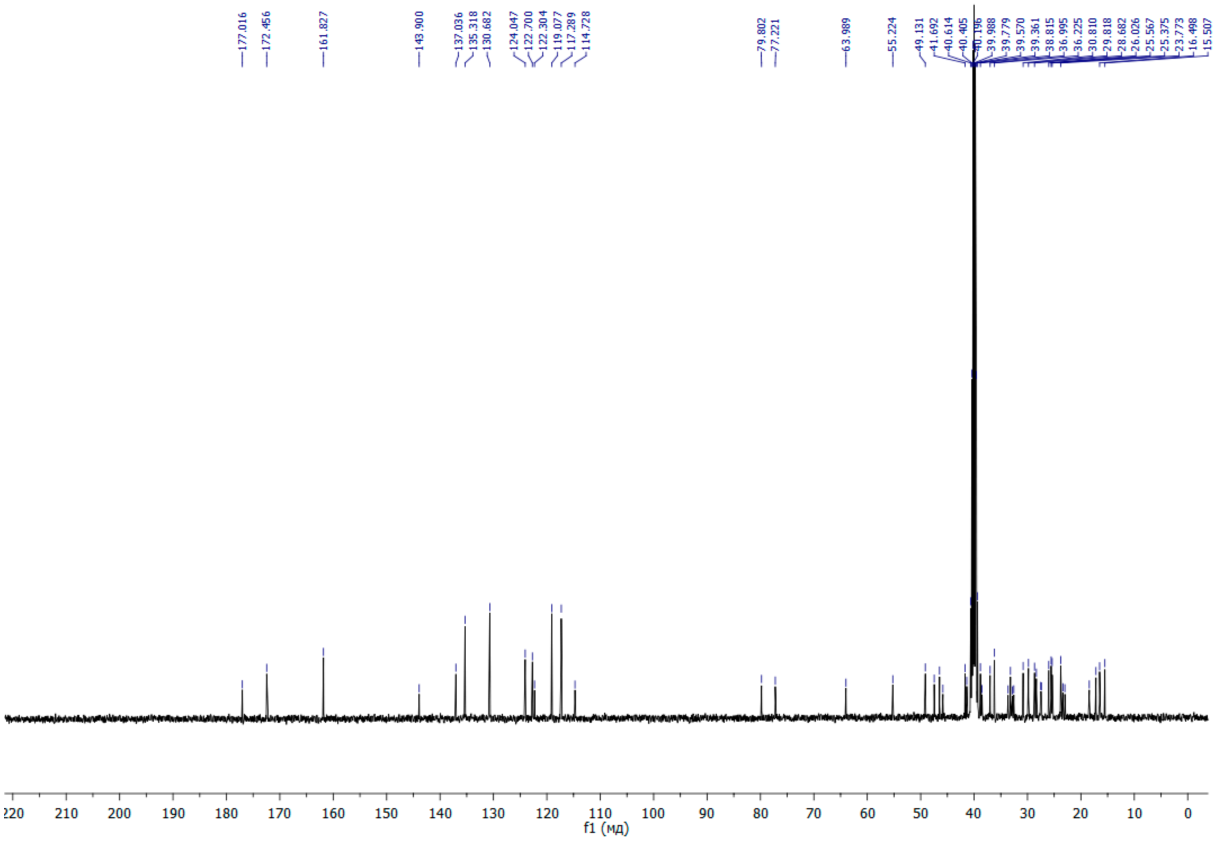

Figure S73. ^{1}H NMR Spectrum of compound **13f** (400 MHz, DMSO-d$_6$)

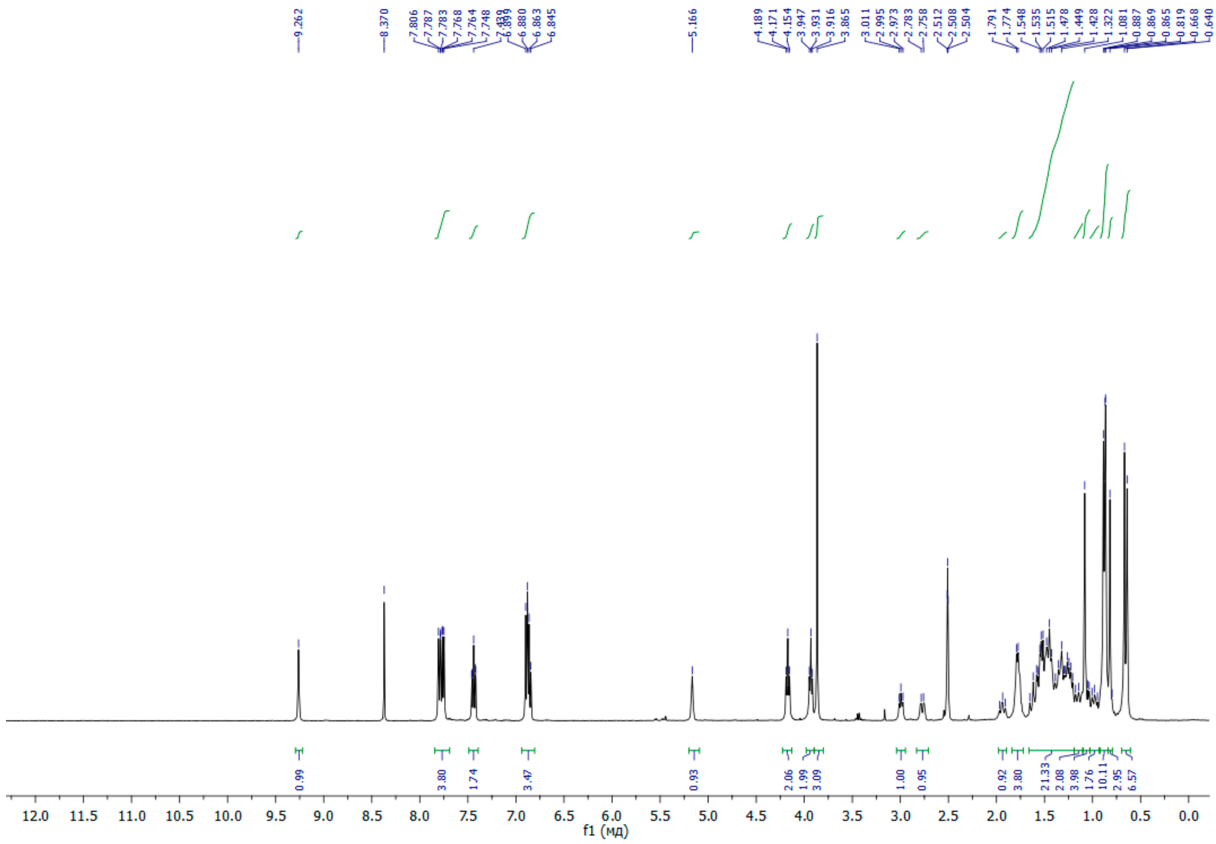

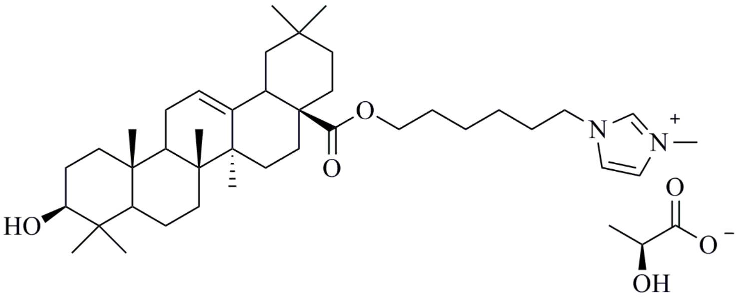

Figure S74. ^{13}C NMR Spectrum of compound **13g** (100 MHz, DMSO-d_6)

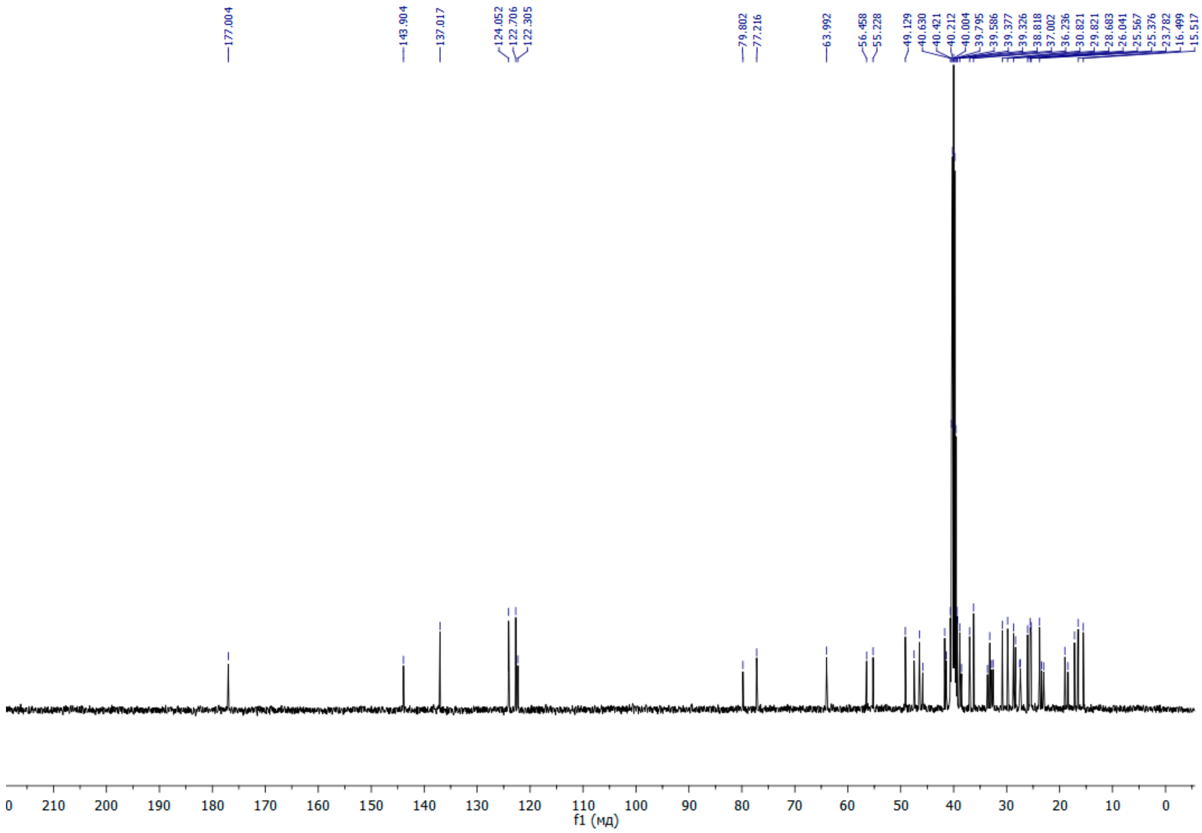

Figure S75. ^{1}H NMR Spectrum of compound **13g** (400 MHz, DMSO-d$_6$)

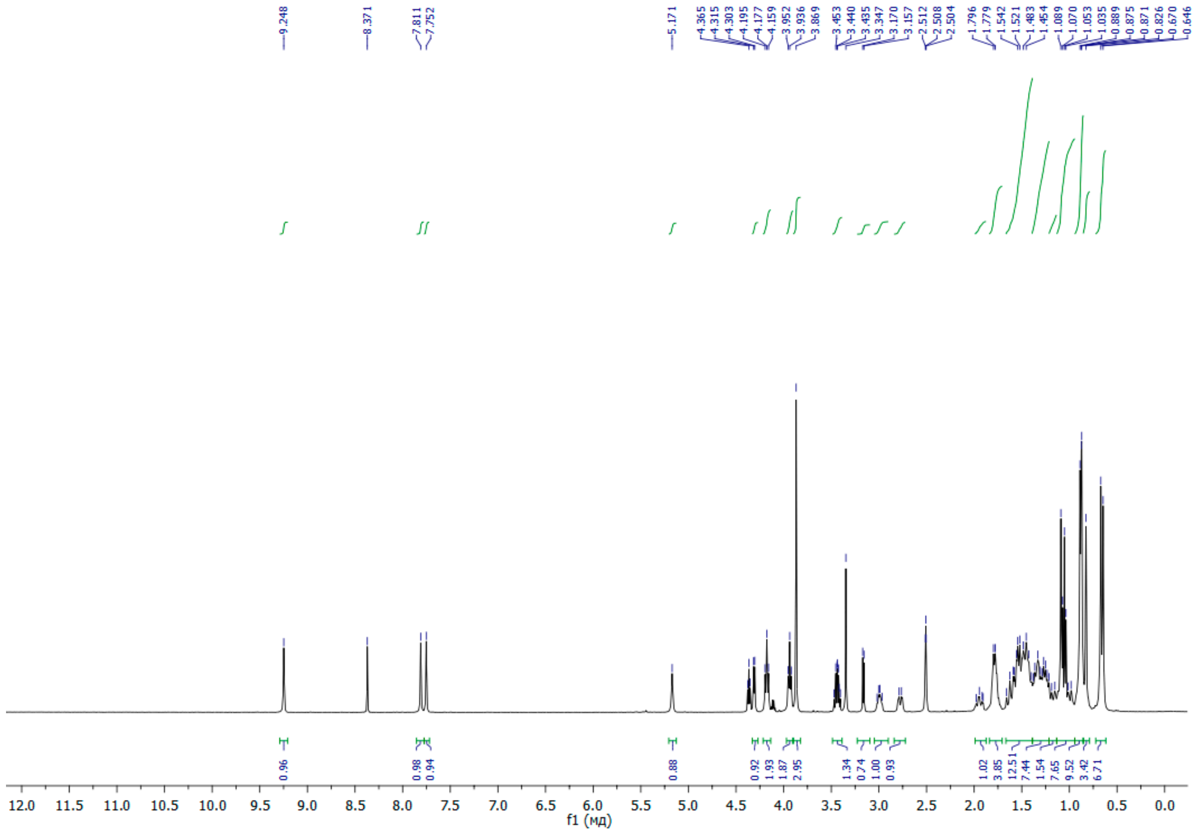

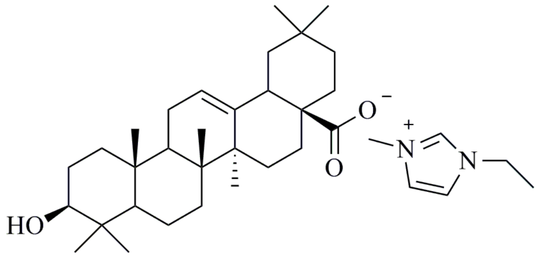

Figure S76. ^{13}C NMR Spectrum of compound **15a** (100 MHz, DMSO-d_6)

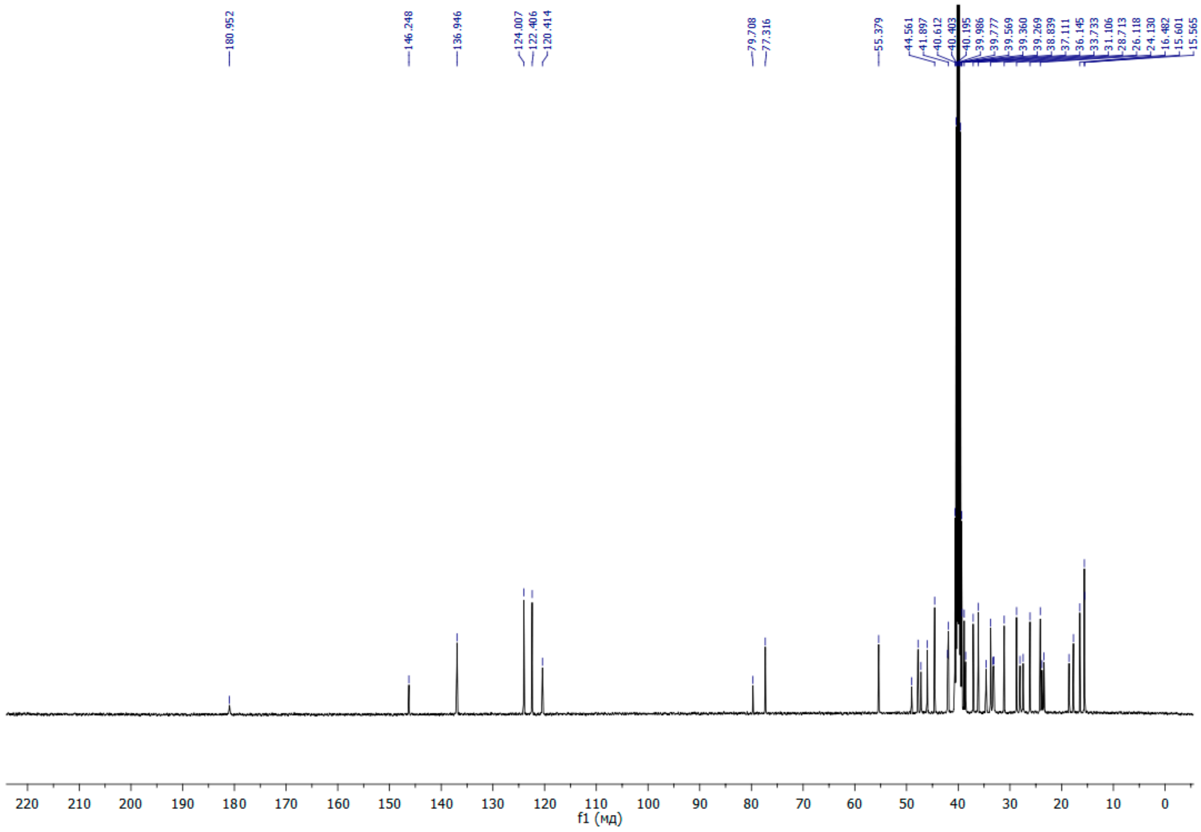

Figure S77. ^{1}H NMR Spectrum of compound **15a** (400 MHz, DMSO-d$_6$)

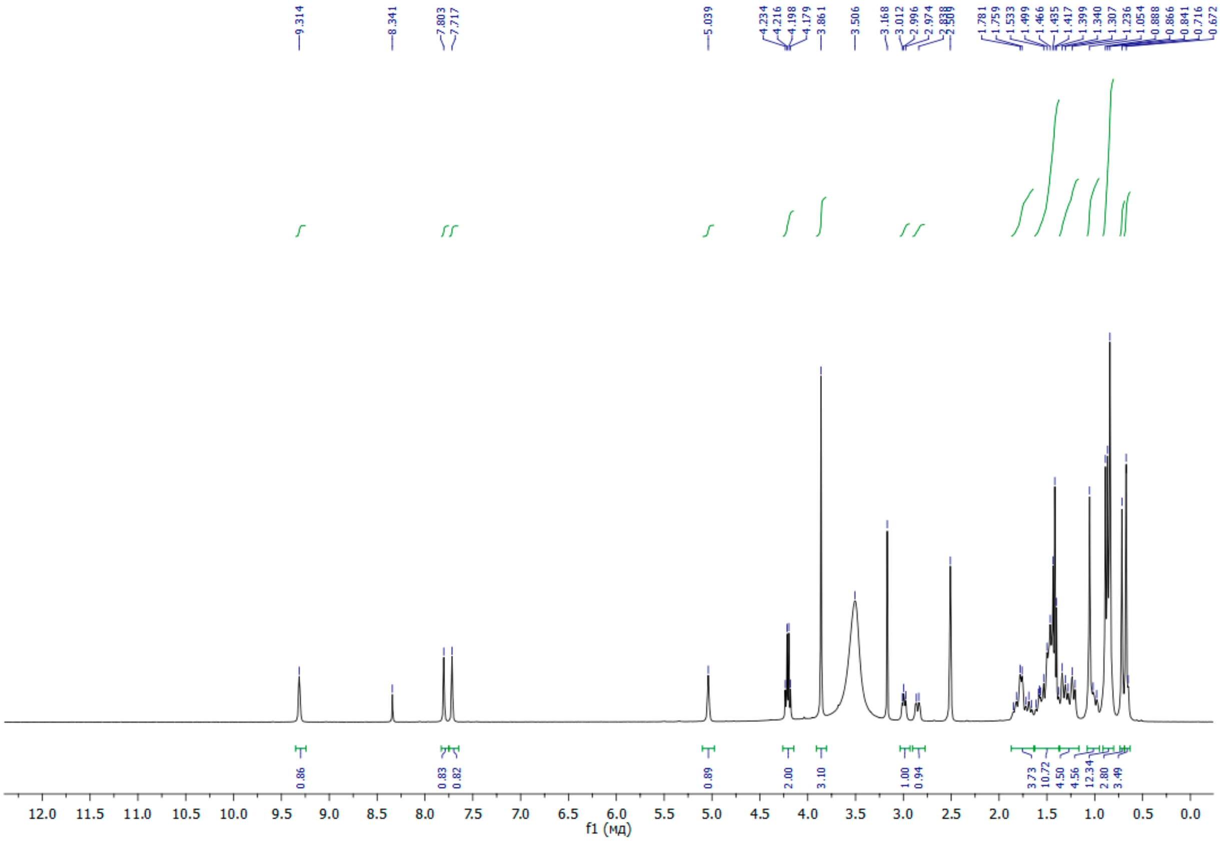

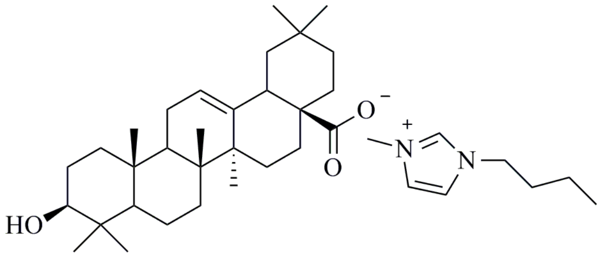

Figure S78. ^{13}C NMR Spectrum of compound **15b** (100 MHz, DMSO-d_6)

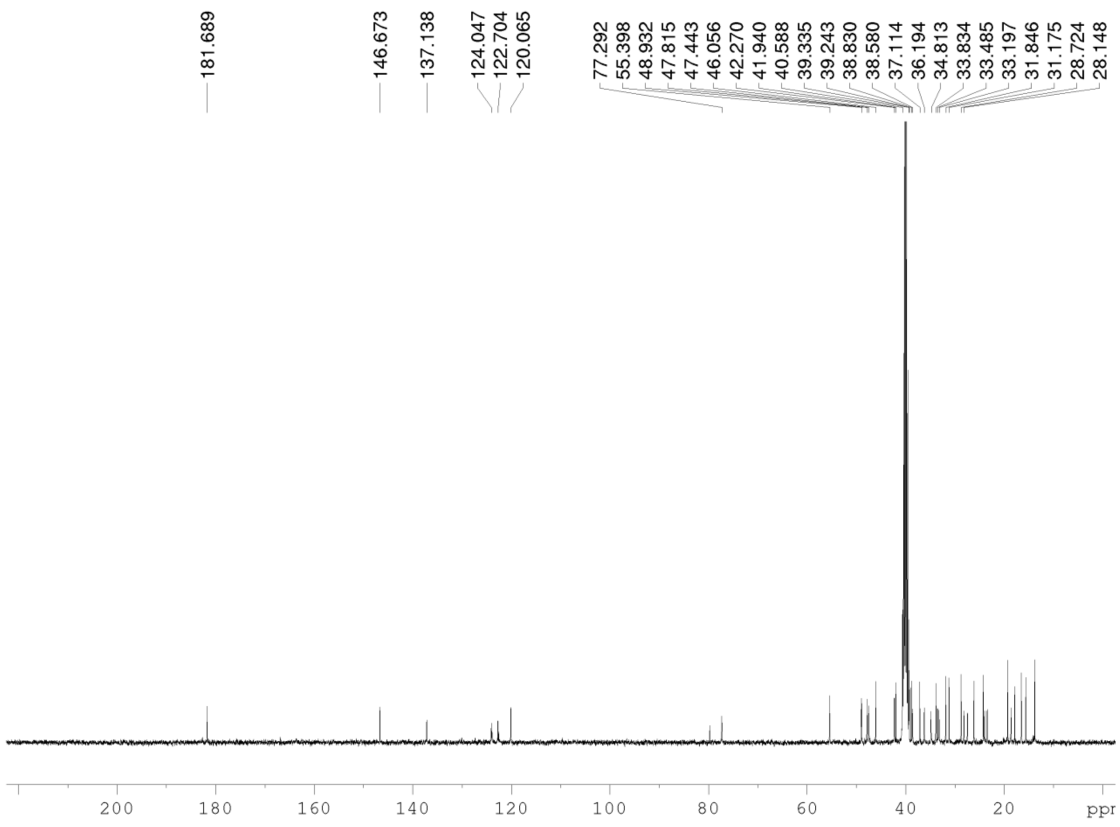

Figure S79. ^{1}H NMR Spectrum of compound **15b** (400 MHz, DMSO-d_6)

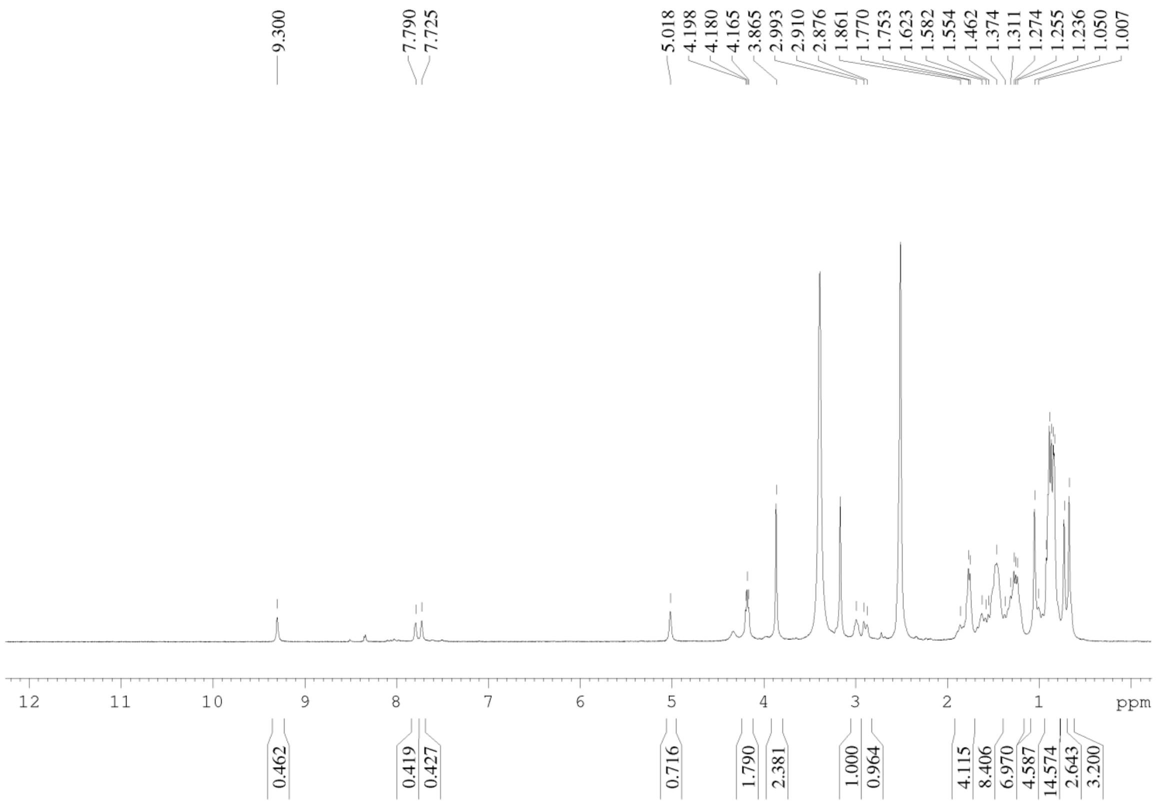

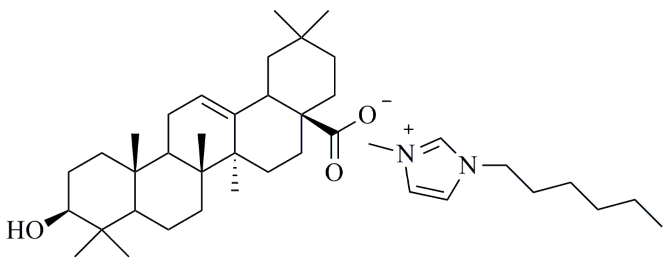

Figure S80. ^{13}C NMR Spectrum of compound **15c** (100 MHz, DMSO-d$_6$)

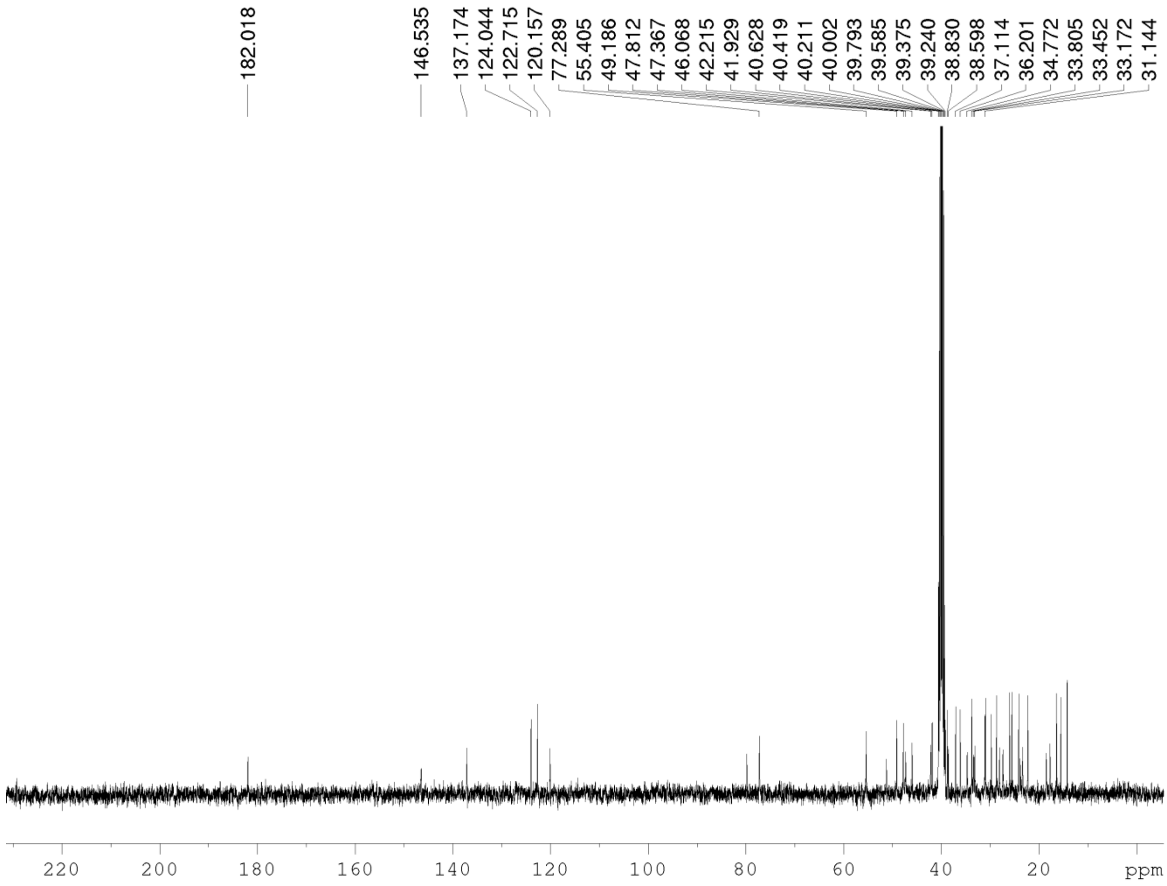

Figure S81. ^1H NMR Spectrum of compound **15c** (400 MHz, DMSO-d_6)

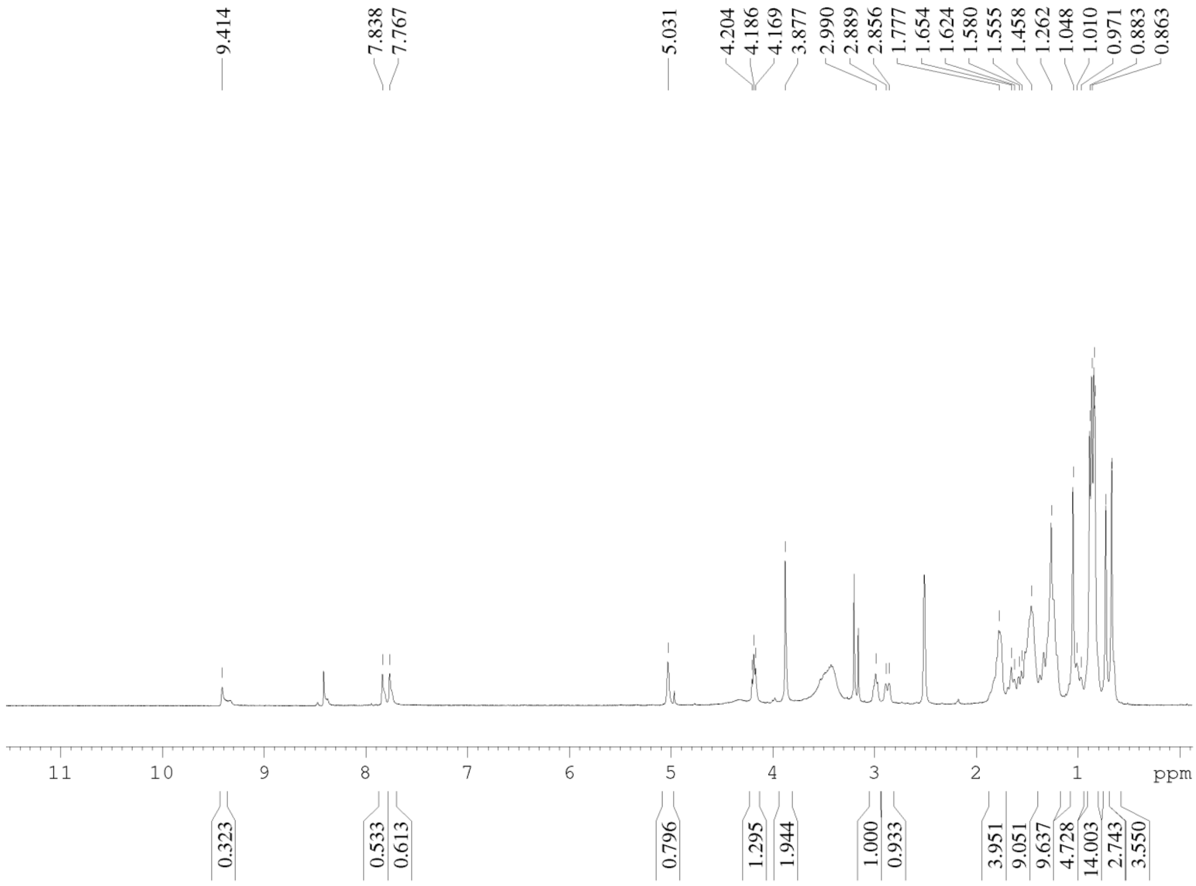

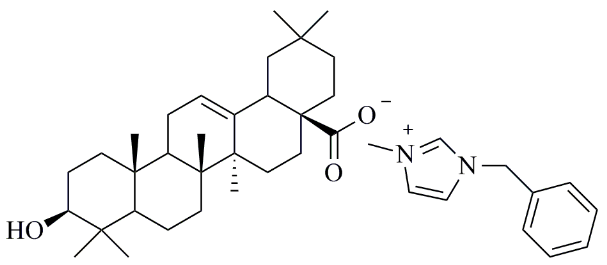

Figure S82. ^{13}C NMR Spectrum of compound **15d** (100 MHz, DMSO-d$_6$)

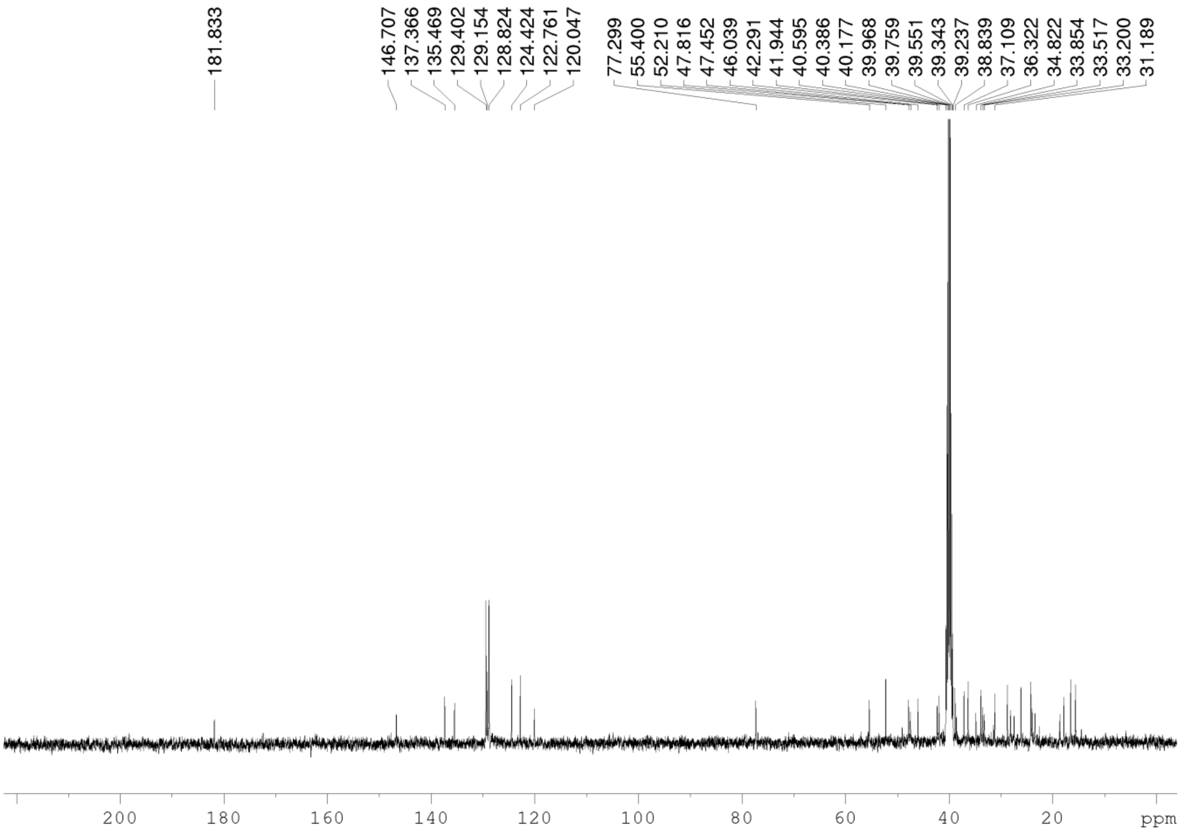

Figure S83. ^{1}H NMR Spectrum of compound **15d** (400 MHz, DMSO-d6)

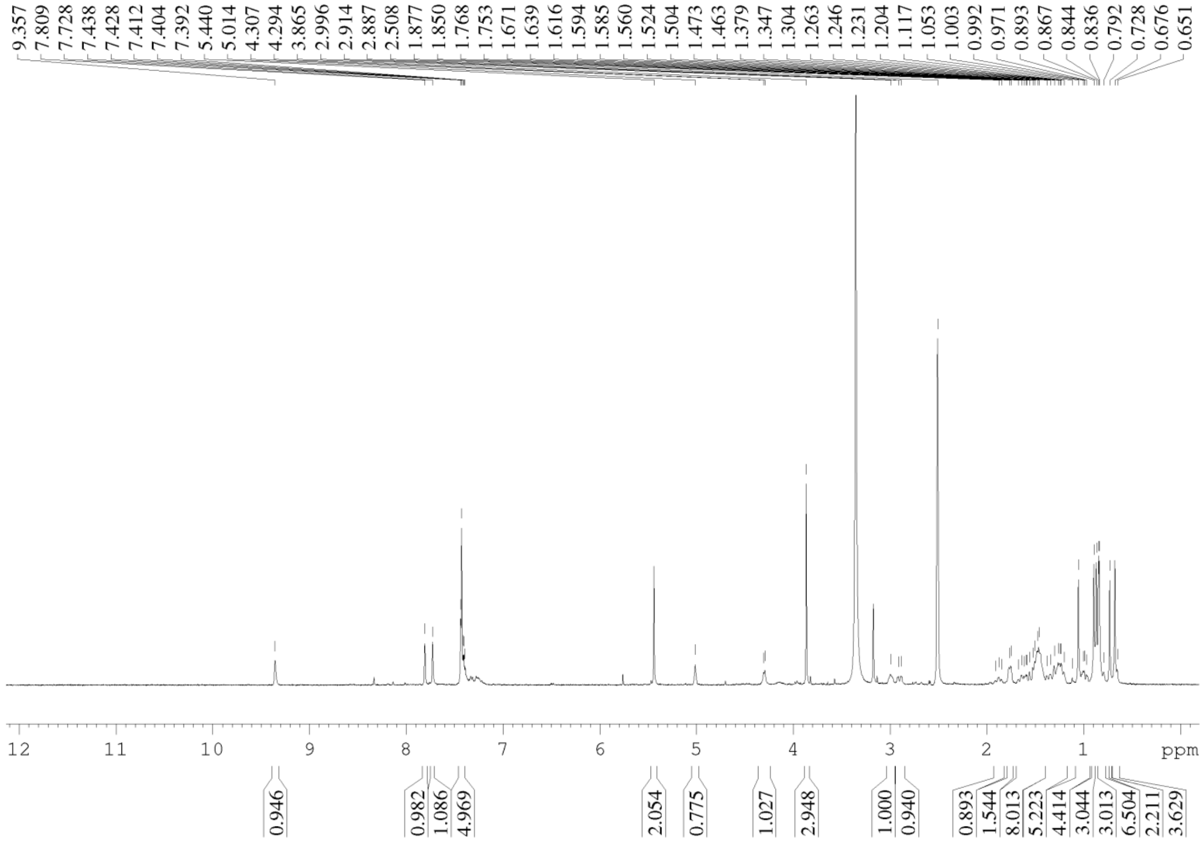

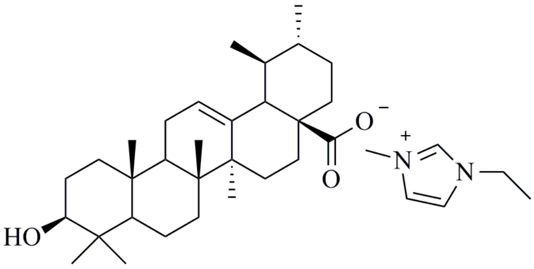

Figure S84. ^{13}C NMR Spectrum of compound **16a** (100 MHz, DMSO-d_6)

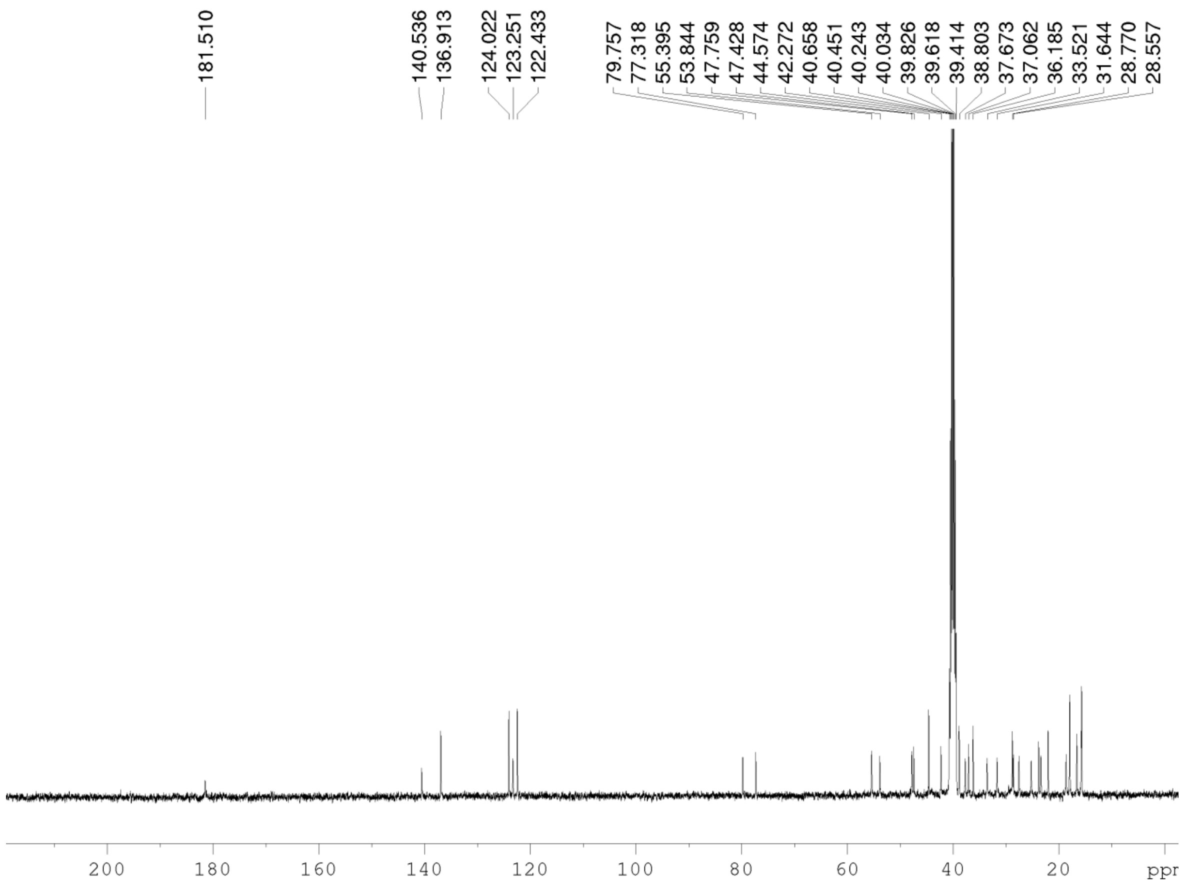

Figure S85. ^{1}H NMR Spectrum of compound **16a** (400 MHz, DMSO-d$_6$)

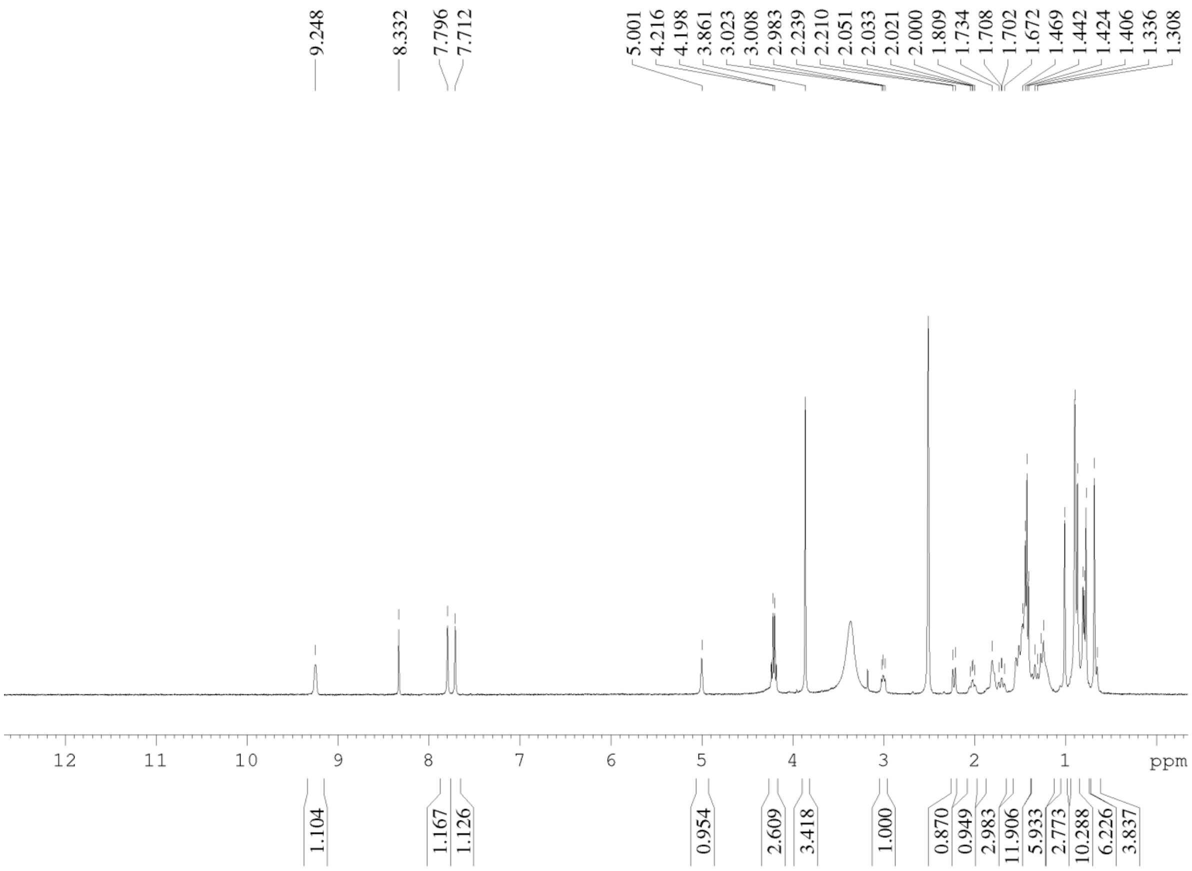

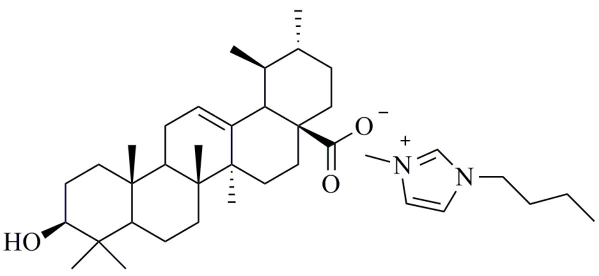

Figure S86. ^{13}C NMR Spectrum of compound **16b** (100 MHz, DMSO-d_6)

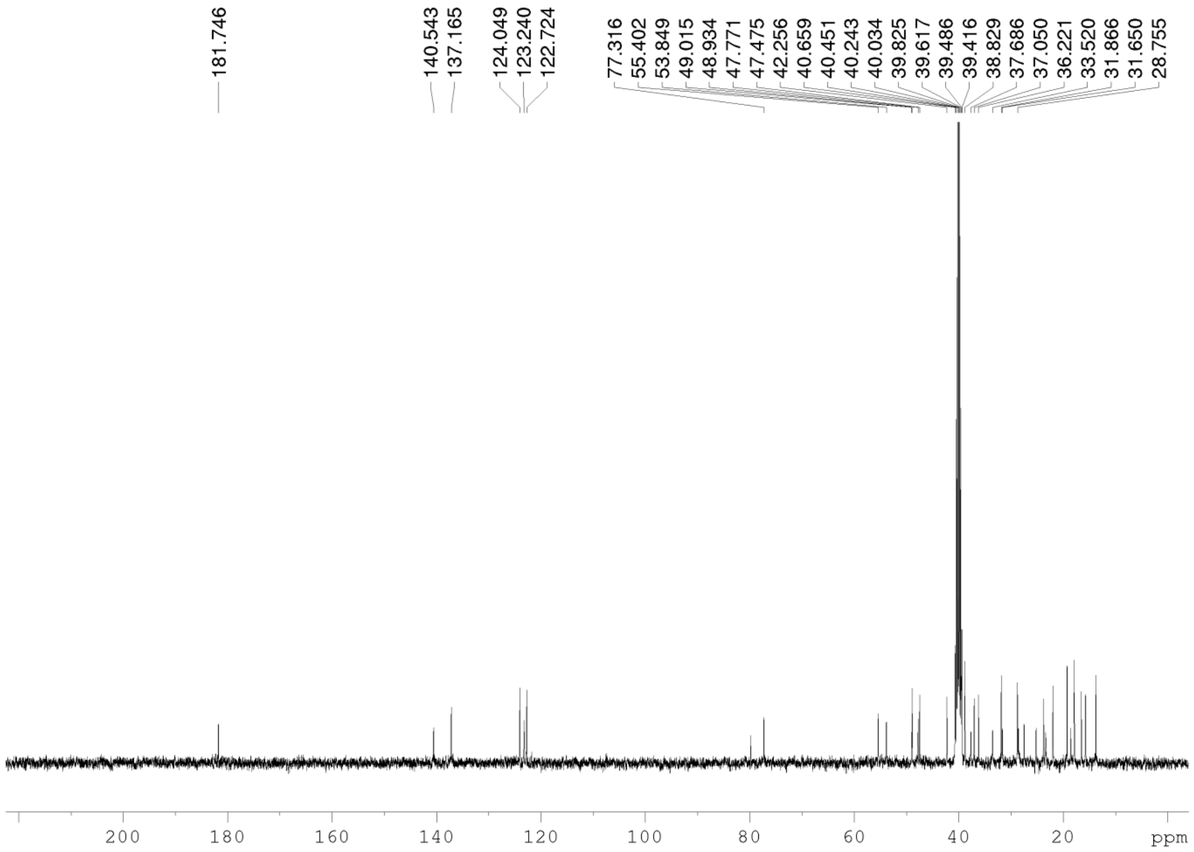

Figure S87. ^{1}H NMR Spectrum of compound **16b** (400 MHz, DMSO-d_6)

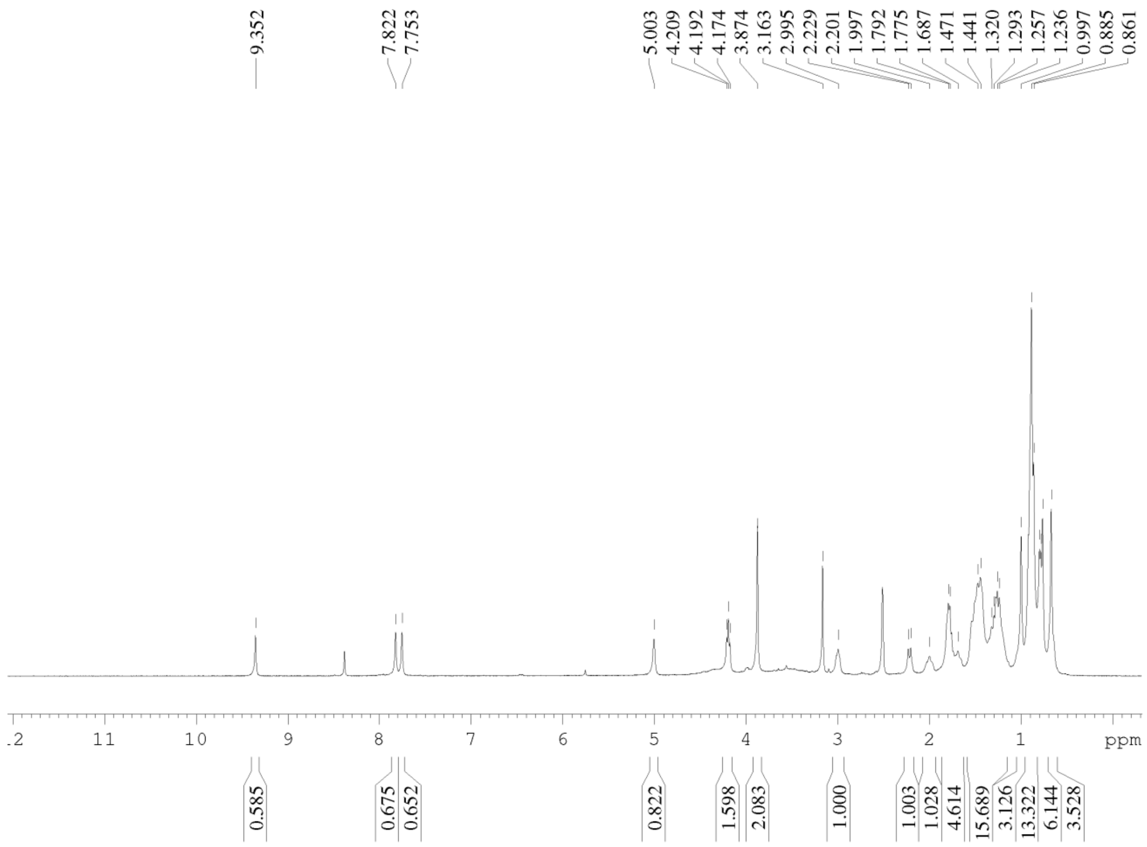

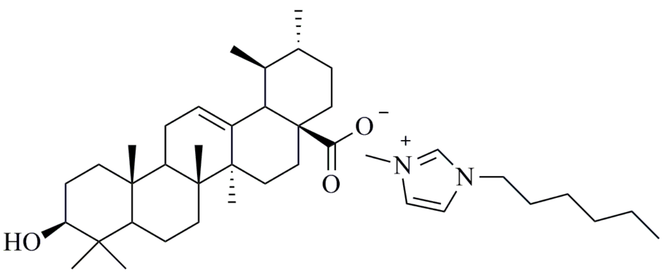

Figure S88. ^{13}C NMR Spectrum of compound **16c** (100 MHz, DMSO-d$_6$)

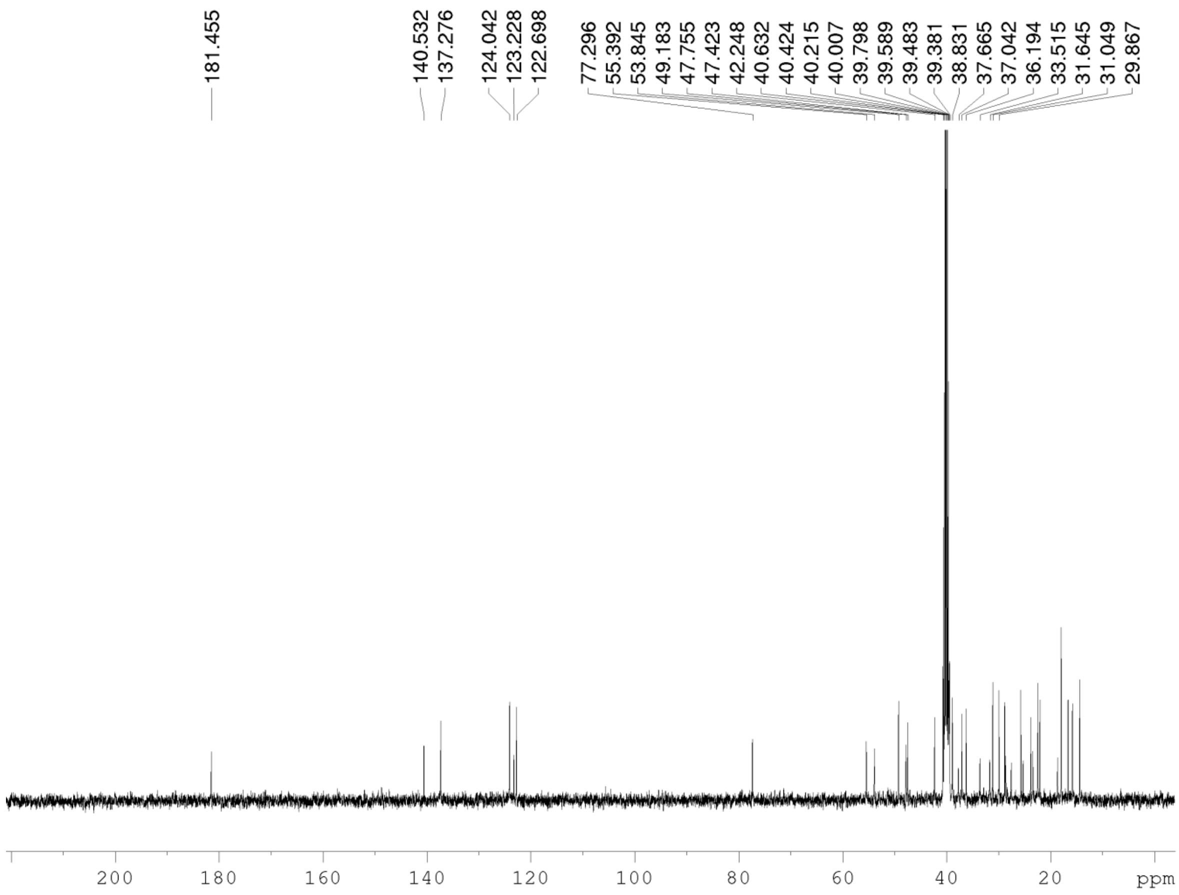

Figure S89. ^{1}H NMR Spectrum of compound **16c** (400 MHz, DMSO-d6)

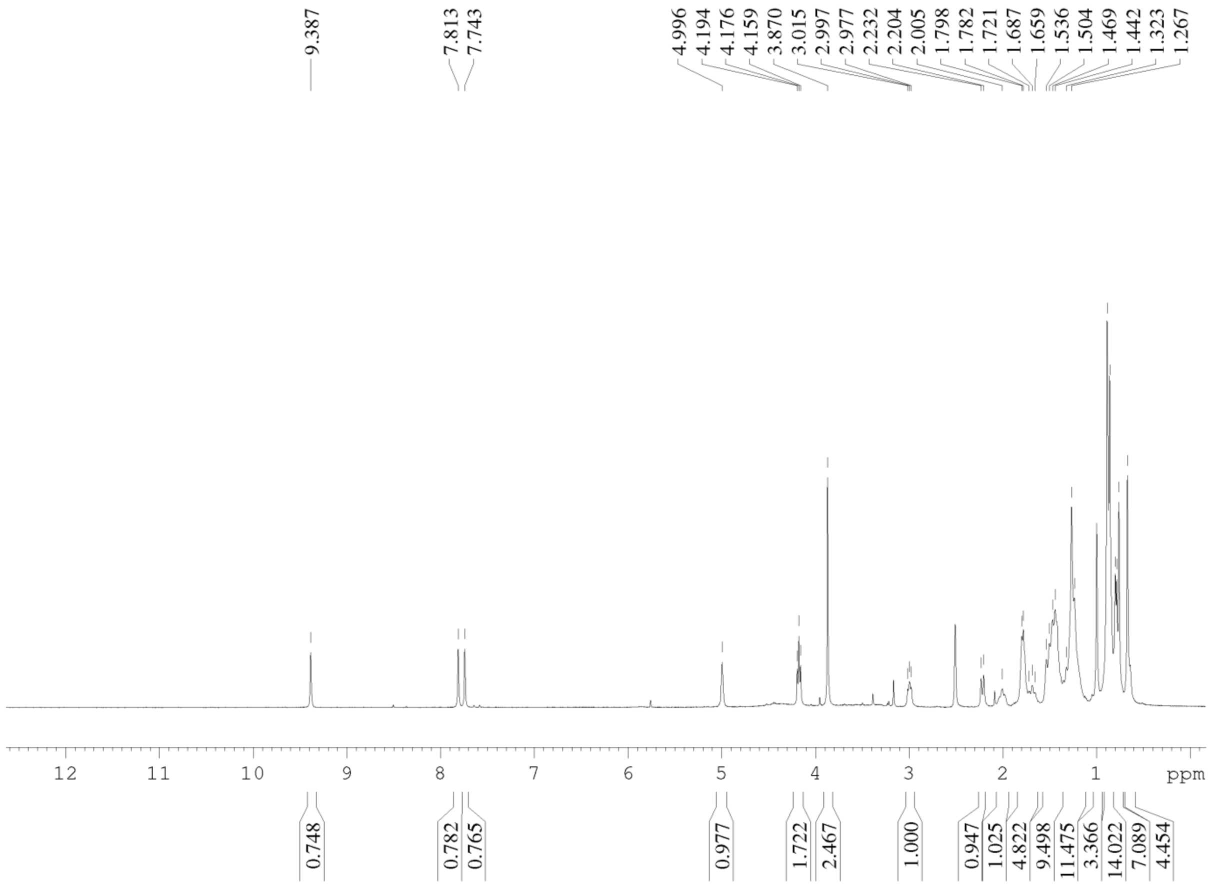

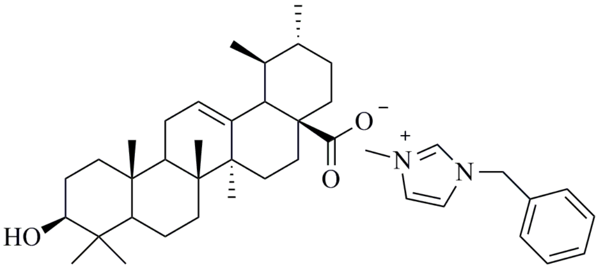

Figure S90. ^{13}C NMR Spectrum of compound **16d** (100 MHz, DMSO-d_6)

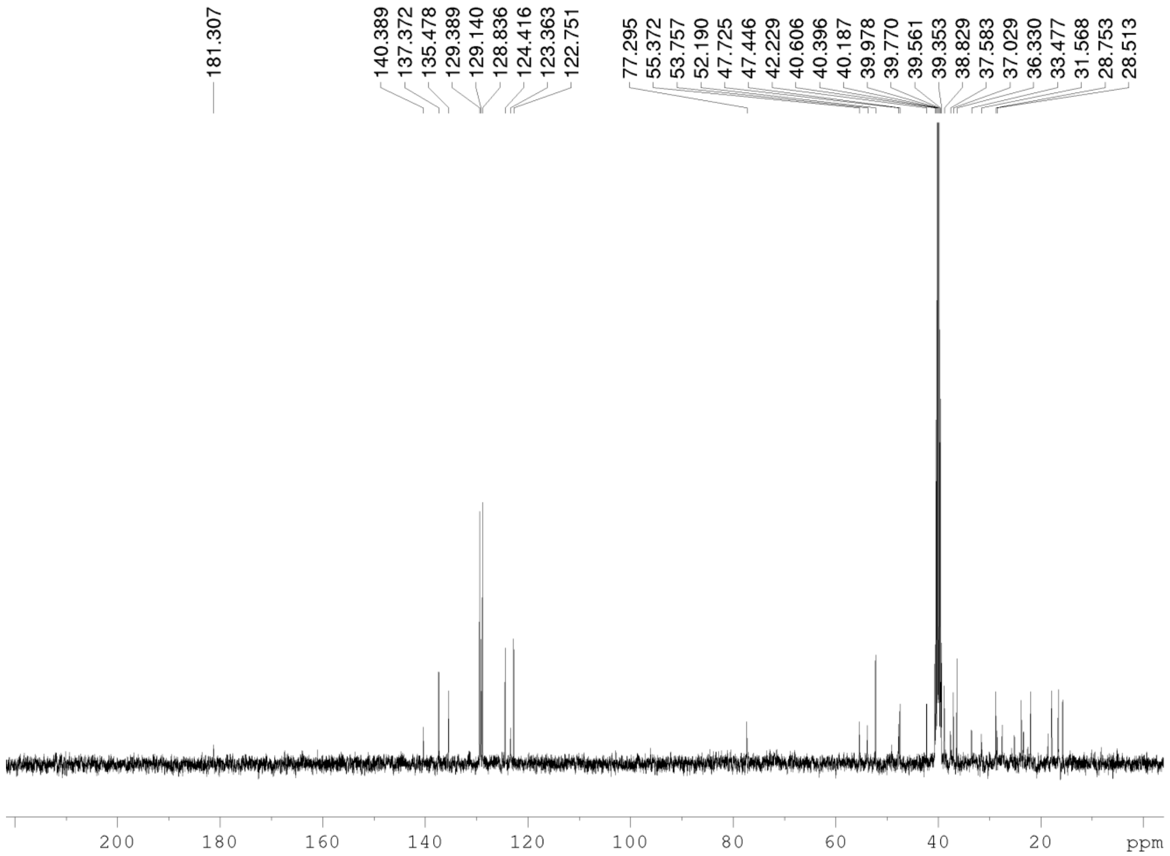

Figure S91. ^{1}H NMR Spectrum of compound **16d** (400 MHz, DMSO-d6)

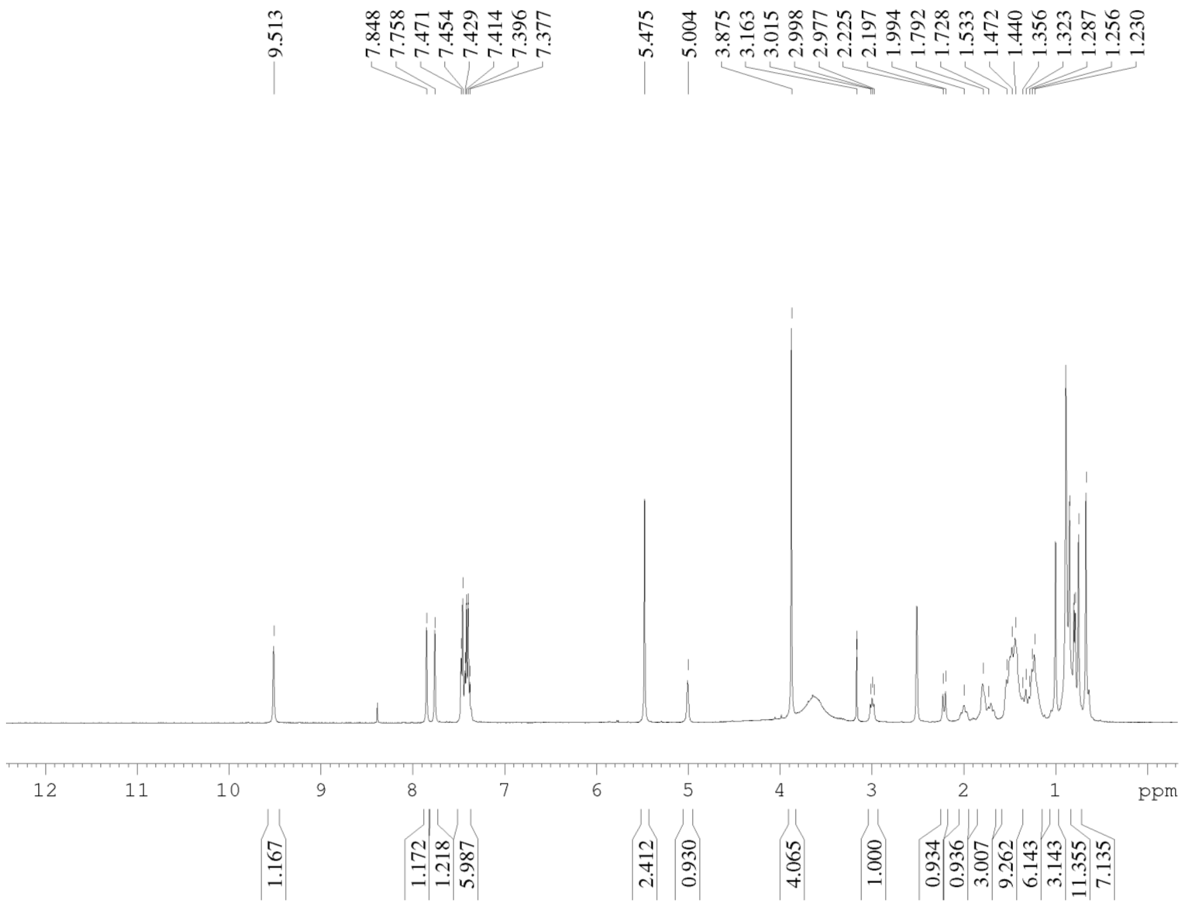

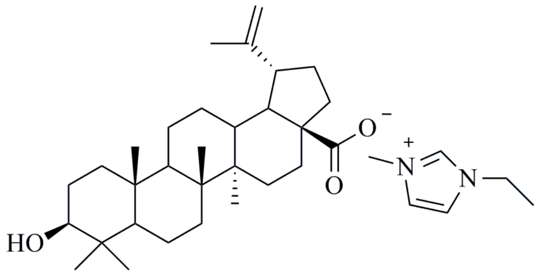

Figure S92. ^{13}C NMR Spectrum of compound **17a** (100 MHz, DMSO-d$_6$)

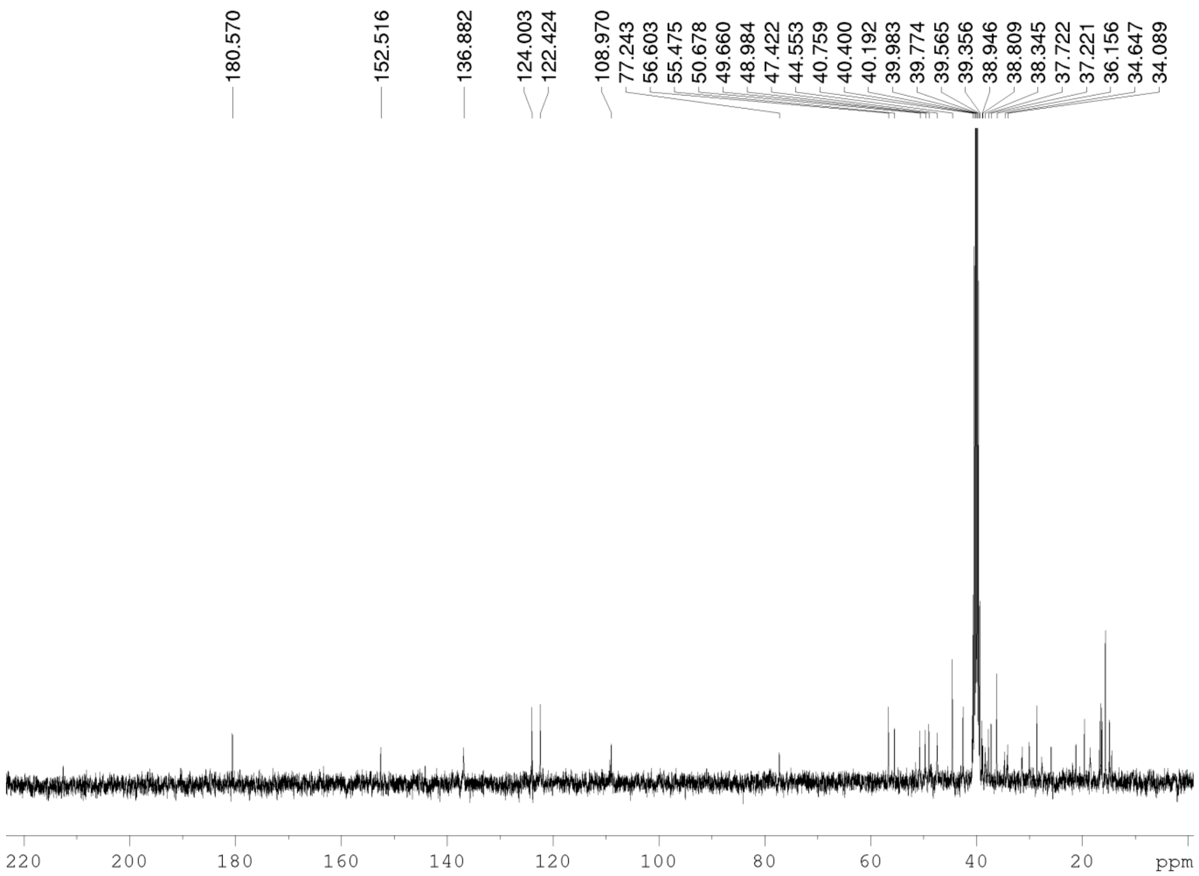

Figure S93. ^{1}H NMR Spectrum of compound **17a** (400 MHz, DMSO-d$_6$)

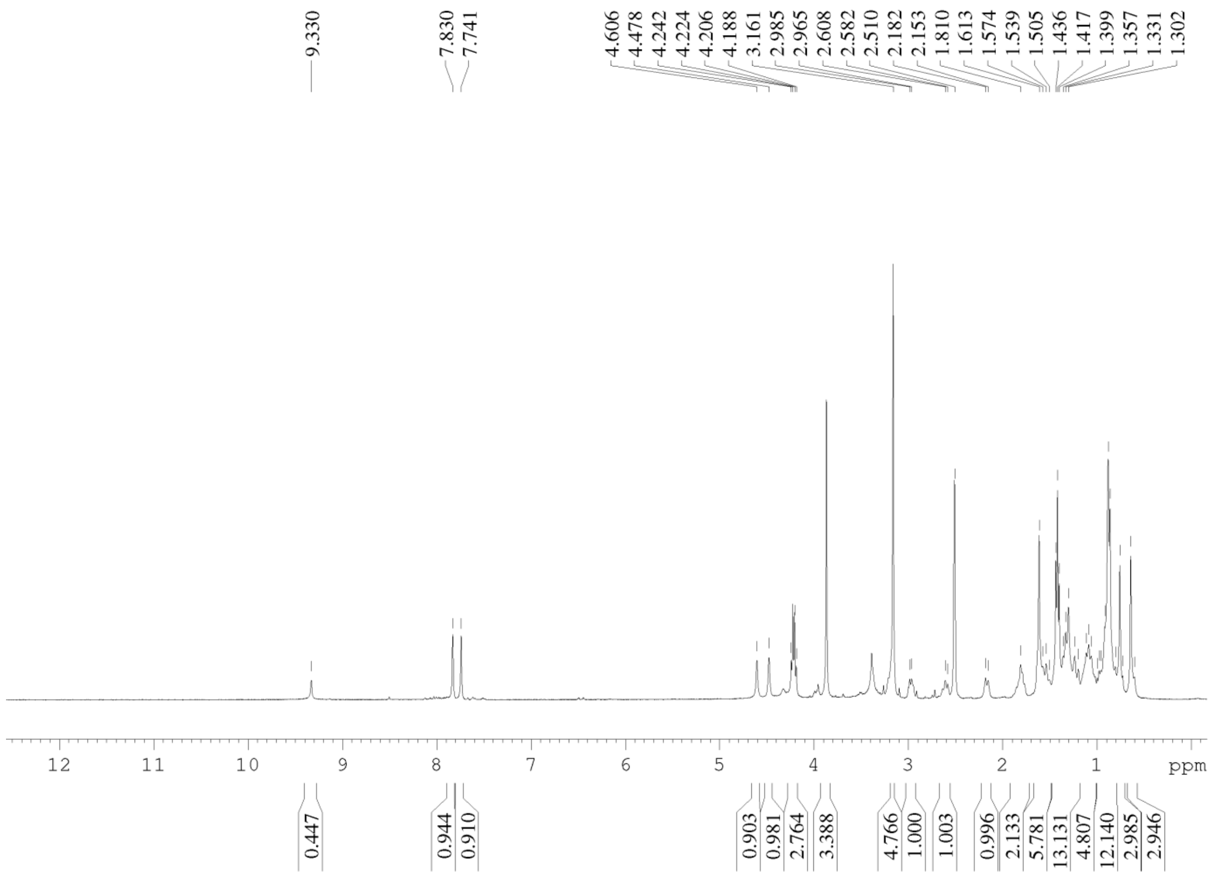

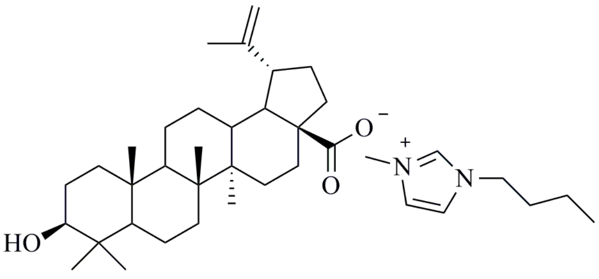

Figure S94. ^{13}C NMR Spectrum of compound **17b** (100 MHz, DMSO-d$_6$)

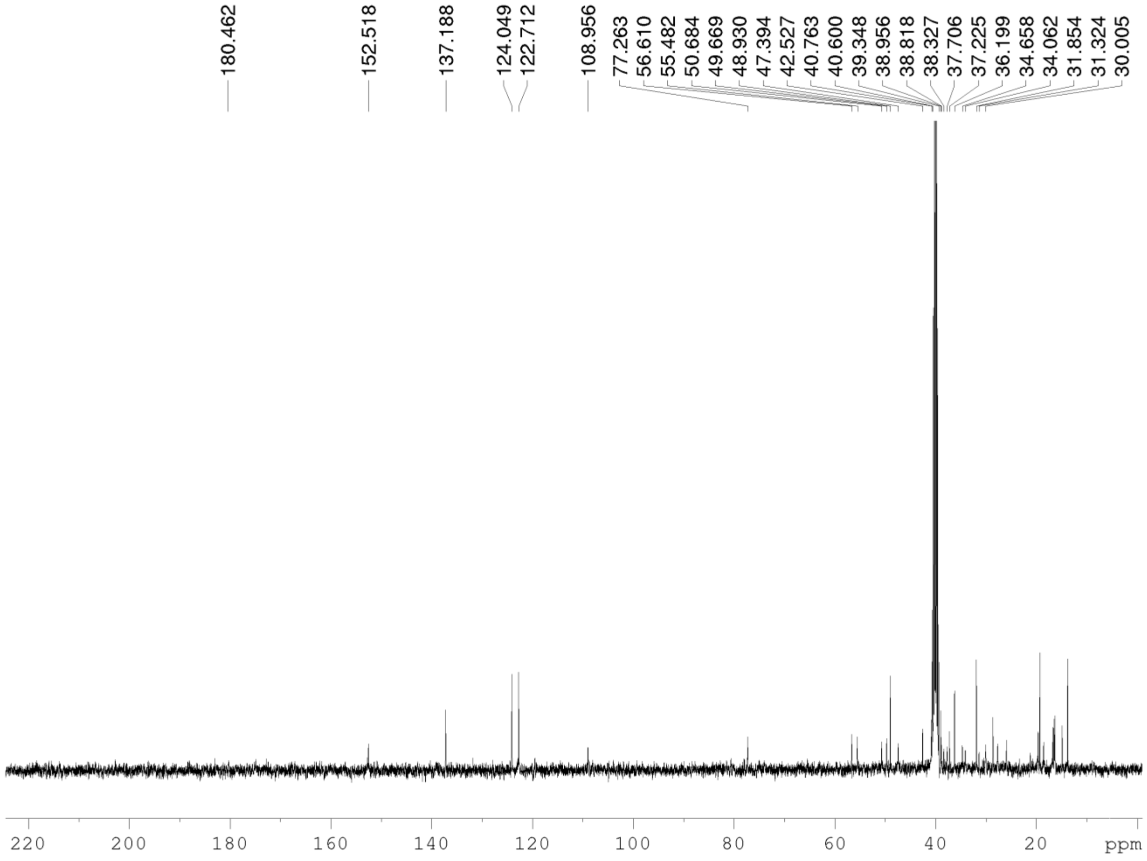

Figure S95. ^{1}H NMR Spectrum of compound **17b** (400 MHz, DMSO-d$_6$)

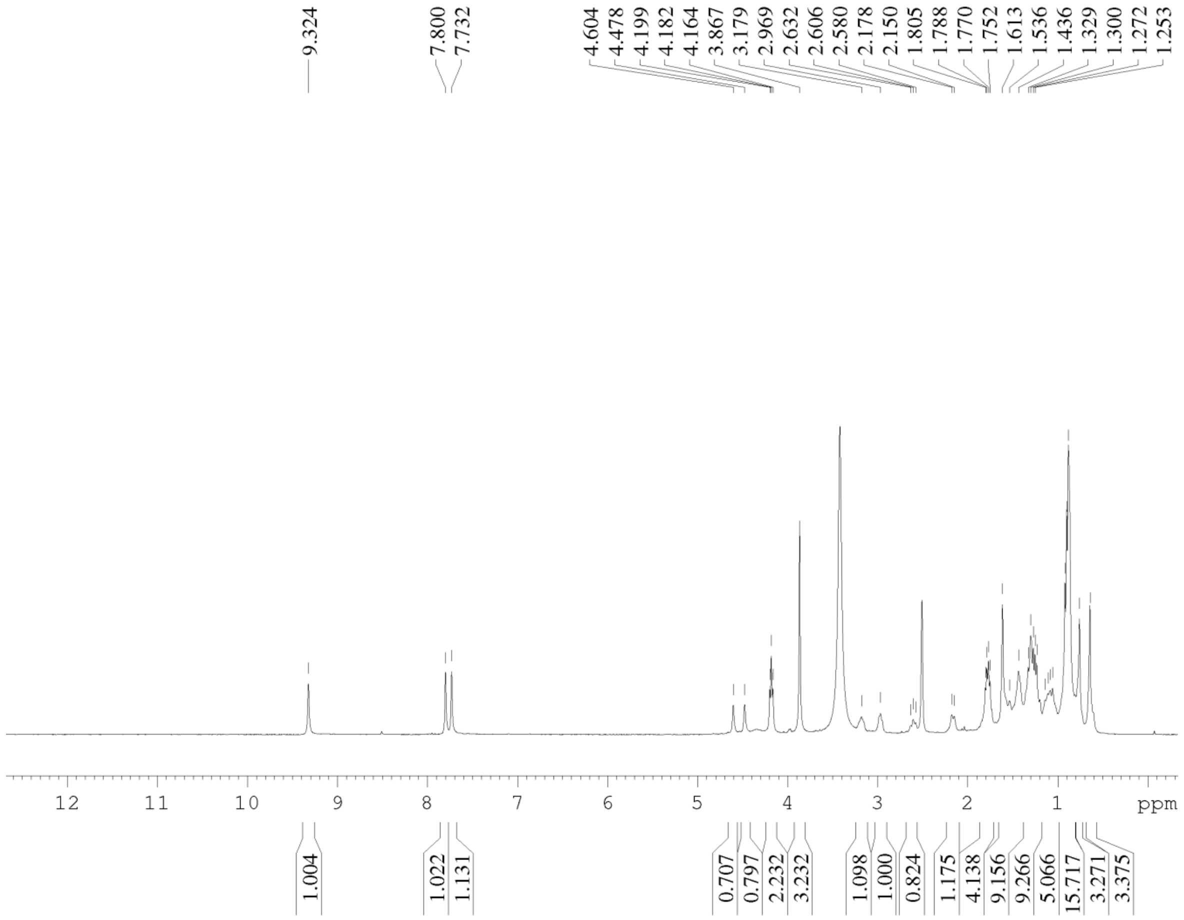

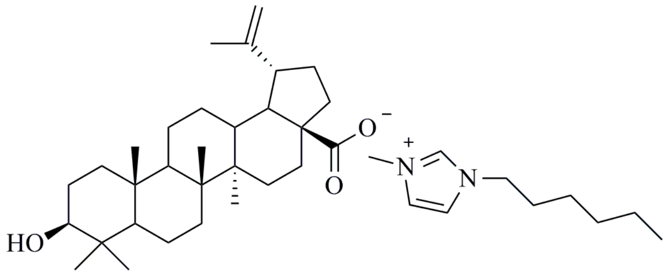

Figure S96. ^{13}C NMR Spectrum of compound **17c** (100 MHz, DMSO-d$_6$)

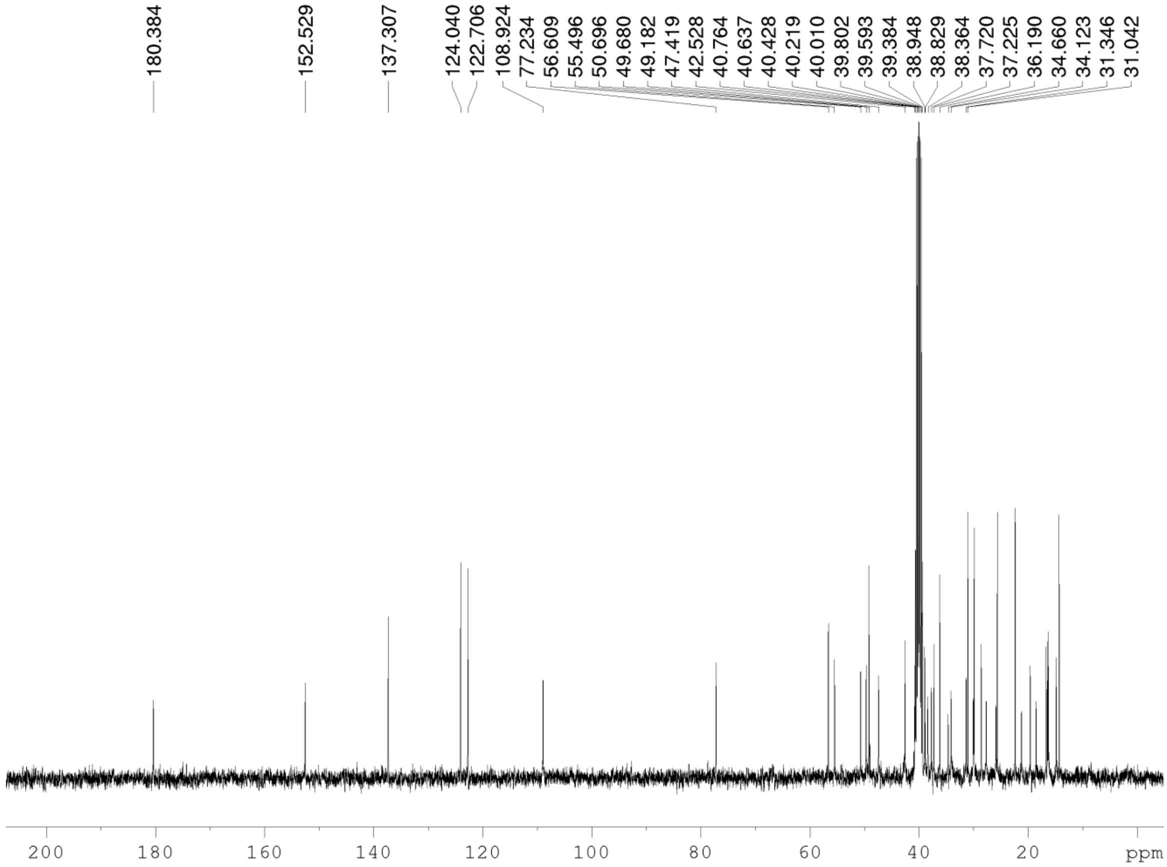

Figure S97. ^{1}H NMR Spectrum of compound **17c** (400 MHz, DMSO-d6)

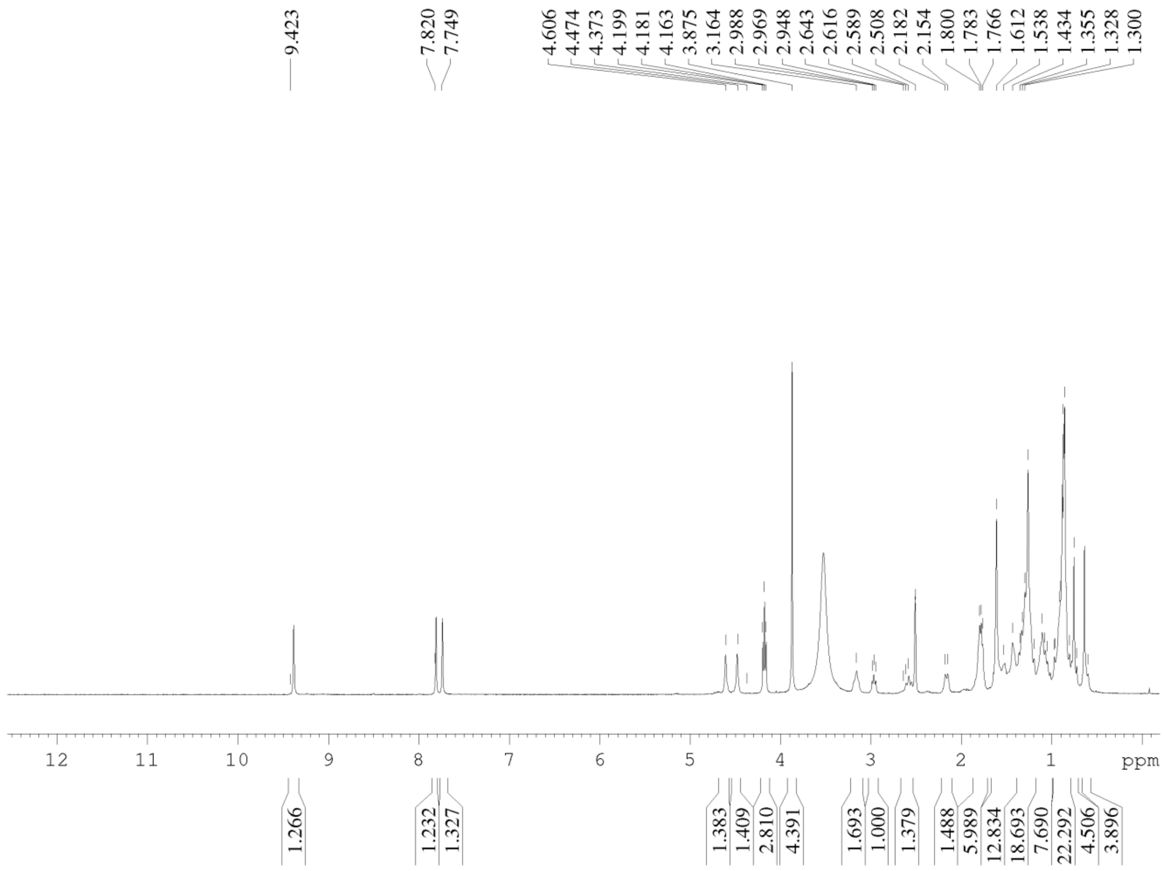

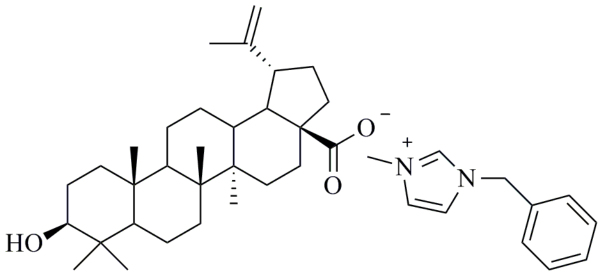

Figure S98. ^{13}C NMR Spectrum of compound **17d** (100 MHz, DMSO-d_6)

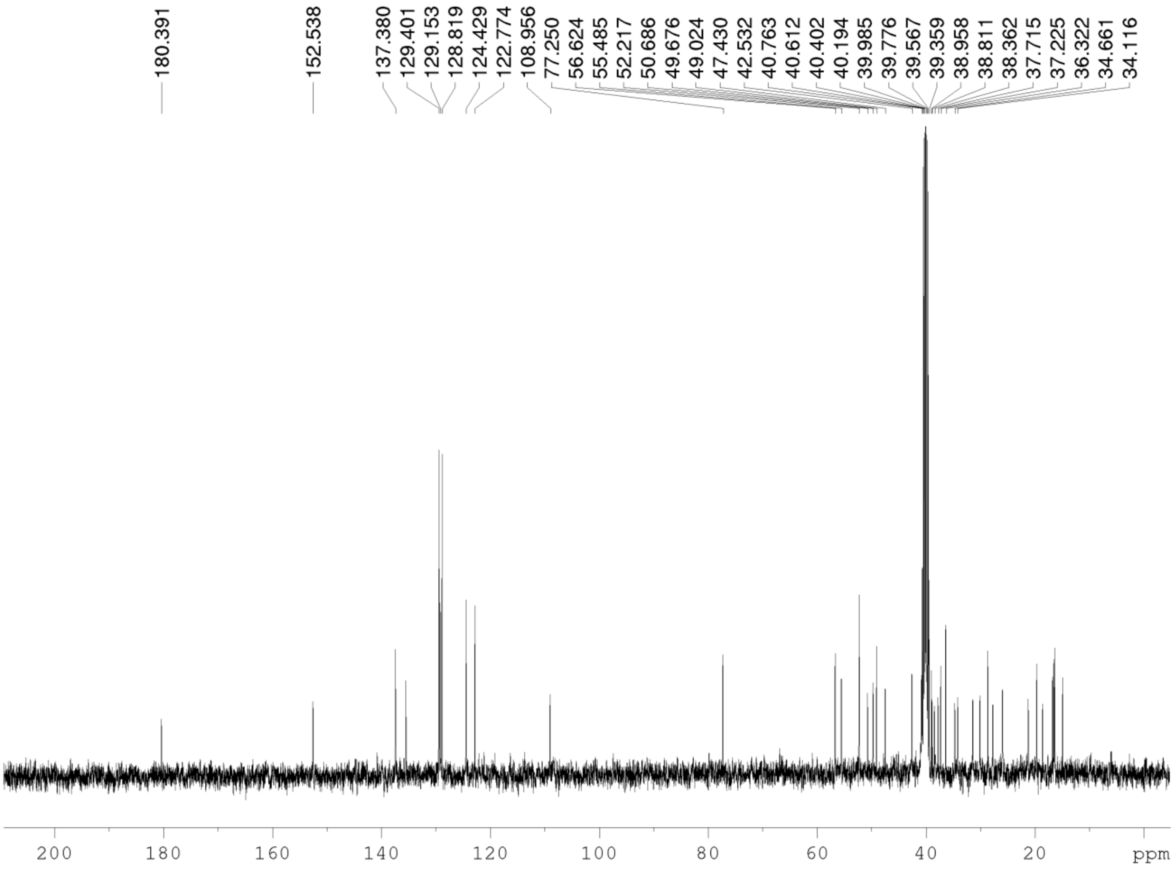

Figure S99. ^{1}H NMR Spectrum of compound **17d** (400 MHz, DMSO-d_6)

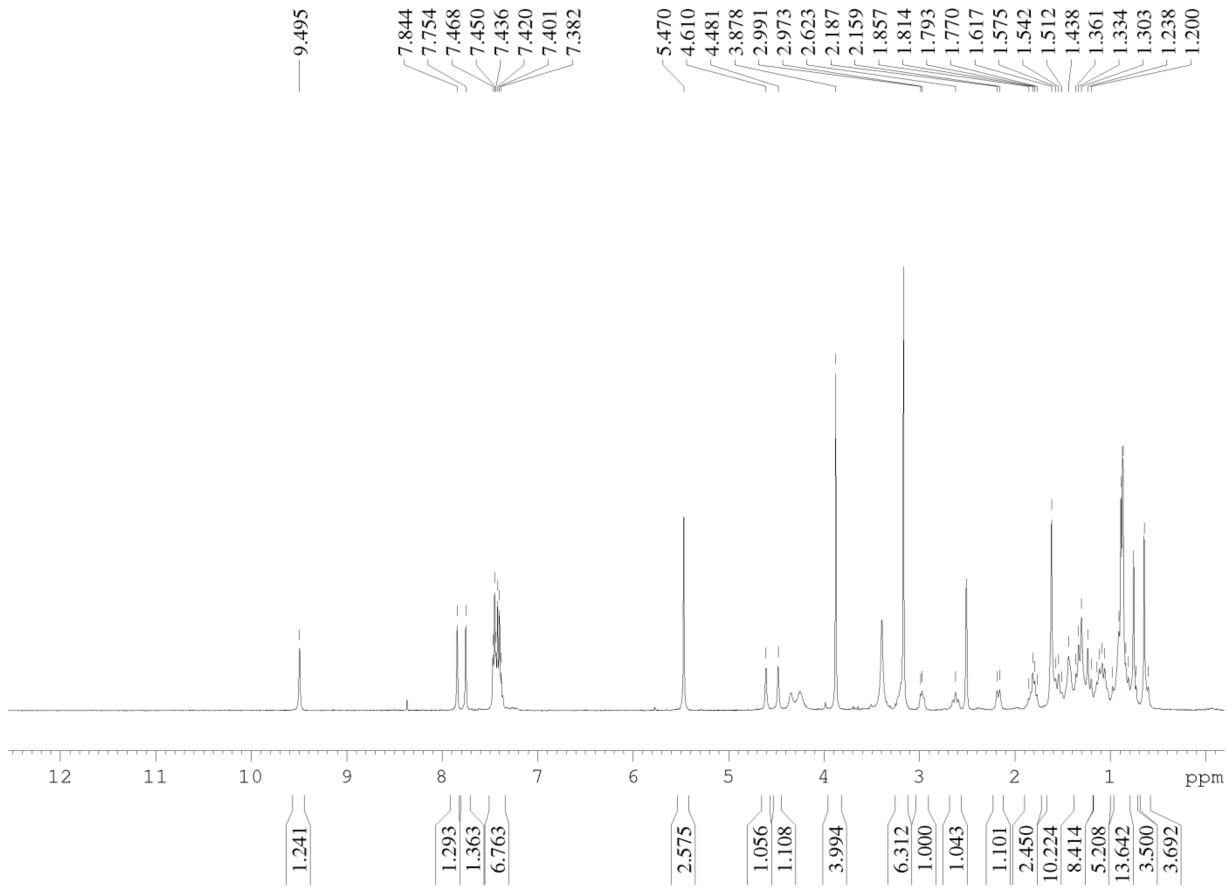

Supplement: Supplementary file 1 [file cancers-15-00756-s001.zip › cancers-2136434-supplementary.pdf]
